# Bright triplet and bright charge-separated singlet excitons in organic diradicals enable optical read-out and writing of spin states

**DOI:** 10.1038/s41557-025-01875-z

**Published:** 2025-07-29

**Authors:** Rituparno Chowdhury, Petri Murto, Naitik A. Panjwani, Yan Sun, Pratyush Ghosh, Yorrick Boeije, Chiara Delpiano Cordeiro, Vadim Derkach, Seung-Je Woo, Oliver Millington, Daniel G. Congrave, Yao Fu, Tarig B. E. Mustafa, Miguel Monteverde, Jesús Cerdá, Giacomo Londi, Jan Behrends, Akshay Rao, David Beljonne, Alexei Chepelianskii, Hugo Bronstein, Richard H. Friend

**Affiliations:** 1 The Cavendish Laboratory, University of Cambridge, Cambridge, UK; 2 Yusuf Hamied Department of Chemistry, University of Cambridge, Cambridge, UK; 3 Berlin Joint EPR Lab, Fachbereich Physik, Freie Universität Berlin, Berlin, Germany; 4 LPS, Université Paris-Saclay, CNRS, UMR 8502, Orsay, France; 5 O. Ya. Usikov Institute for Radiophysics and Electronics, National Academy of Sciences of Ukraine, Kharkiv, Ukraine; 6 Department of Chemistry, University of Oxford, Oxford, UK; 7 Laboratory for Chemistry of Novel Materials, University of Mons, Mons, Belgium; 8 Department of Chemistry and Industrial Chemistry, University of Pisa, Pisa, Italy

**Keywords:** Materials chemistry, Excited states

## Abstract

Optical control of electron spin states is important for quantum sensing and computing applications, as developed with the diamond nitrogen vacancy centre. This requires electronic excitations, excitons, with net spin. Here we report a molecular diradical where two trityl radical groups are coupled via a meta-linked fluorene bridge. The singlet exciton is at lower energy than the triplet because electron transfer from one of the radical non-bonding orbitals to the other is spin allowed, set by the charging energy for the double occupancy of the non-bonding level, the Hubbard *U*. Both excitons give efficient photoluminescence at 640 and 700 nm with near unity efficiency. The ground state exchange energy is low, 60 µeV, allowing control of ground state spin populations. We demonstrate spin-selective intersystem crossing and show coherent microwave control. We report up to 8% photoluminescence contrast at microwave resonance. This tuning of the singlet Mott–Hubbard exciton against the ‘bandgap’ exciton provides a new design platform for spin–optical materials.

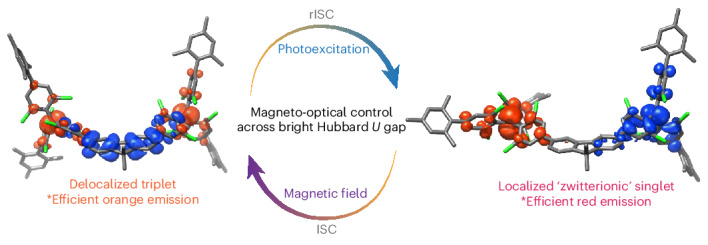

## Abstract (web summary)

Controlling electron spin states with light is vital for quantum technologies but requires electronic excitations with net spin. Now a molecular diradical with two trityl radical groups coupled via a meta-linked fluorene bridge has been developed that features luminescent singlet and triplet excitons. The spins in the ground state can be written and read using photons, giving rise to broad magnetic and microwave modulation of the photoluminescence.

## Main

Coupled correlated spin systems form the basis of a wide range of electronic phenomena, from cuprate superconductors^1^ to materials developed for quantum information and sensing^2^. Molecular semiconductors, as used in organic light emitting displays, use closed shell electronic structures, where strong Coulomb interactions give rise to large spin exchange energies separating singlet and triplet excitons. Here, we explore a parallel opportunity to engineer correlated molecular semiconductors with ‘open shell’ spin radical building units that are brought into contact through covalent intramolecular coupling. With coupling selected to keep spins localized to their parent molecular unit, such extended structures behave as Mott–Hubbard insulators^3^. As we develop below, this structure has spin-specific excitations, and for our materials, these are optically controllable.

Optical initialization and read-out of spin states has been of growing interest to the emerging field of quantum technologies with implications in quantum computing^4,5^, communication^6^, teleportation^7^ and sensing^8^. This spin–optical interface and its applications have been demonstrated through solid-state defects^9–11^. In molecular organo-metallic systems, it is possible to create an open-shell, non-zero net spin, ground state, and this has been explored to successfully create spin–optical interfaces^12,13^. Fully organic systems offer geometric and spatial control through readily accessible synthetic chemistry methods. There are reports of coherent control over photoexcited high spin states^14–17^, spin-singlet-based quantum optics^18^ and long coherence times^19^, but these are from weakly luminescent systems so that optical read-out is limited. The materials we report here show spin dependent luminescence, critically with near unity luminescence yields. We note recent reports of trityl diradical systems^20–22^ that show emission from coupled spin states that we discuss later in this study and which are compared in Supplementary Table 2.

Carbon-centred chlorinated trityl monoradicals (TTMs), coupled to electron donors such as carbazole, provide a family of efficient luminescent radicals^23–28^ that show promise as light emitters in red and near-IR LEDs. These show emission from excitons in the doublet manifold, and this avoids dark triplet states found in closed shell systems currently used in organic light emitting displays. High photoluminescence quantum yield (PLQE) is essential for any practical optical read-out or writing of a quantum system. Generally, PLQE for molecular semiconductors is much reduced with intermolecular interactions. However, we have found that high PLQE can be maintained in TTM radical systems brought into intramolecular contact. This allows bright multispin excitons and unlocks new opportunities for spin–optical control.

Our starting point is the finding of luminescence-preserving intermolecular energy exchange between emissive doublet excitons and triplet excitons supported in a molecular semiconductor host^29,30^, and we have used this to engineer LED performance. Moving to intramolecular extended structures, we reported high-spin photogenerated excitons for structures where the TTM radical molecular unit is covalently attached to an anthracene^31^. In the ground state, there is no spin interaction with the anthracene, and when a second TTM is attached to the other side of the anthracene, only a doublet electron spin resonance (ESR) response is seen, demonstrating that there is no spin interaction between the two radicals. However, on photoexcitation, high-spin quartet (monoradical) and quintet spin state (biradical) excitons are photogenerated due to coupling to a triplet exciton on the anthracene (selected because it is energy degenerate with the doublet exciton). Spin manipulation is possible in the excited state, and optical read-out via the doublet emission is achieved by reverse intersystem crossing (rISC) to the doublet, which is the only emissive exciton. Note that this does not provide opportunity to manipulate the overall ground state spin nor to read out the ground state from photoluminescence (PL)^31^.

**Fig. 1:**
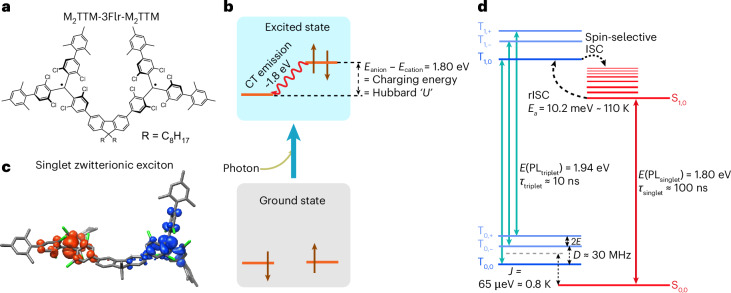
Molecular design of luminescent diradical spin–optical units. **a**, The molecular structure of the M_2_TTM-3Flr-M_2_TTM diradical. The two TTM groups are protected by mesityl groups, and the meta-linkage is through the 3- and 6-positions on the fluorene bridge. **b**, A scheme showing charge transfer from one non-bonding orbital to the other, to set up a spin zero zwitterionic exciton. **c**, Modelled zwitterionic-singlet exciton with anionic doubly occupied non-bonding (blue) and cationic unoccupied non-bonding (red) levels. **d**, An energy level diagram for ground and excited states of the singlet and triplets, shown at close to zero magnetic field. The energy values as determined from experiment are indicated. **d** and **e** are the axial and rhombic components of the zero-field splitting tensor. Source data

There is current interest in diradical systems, ranging from weak coupling^20,21^ through to systems with strong conjugation where the ground state is closed shell (termed diradicaloid)^32–34^. We report here organic diradicals using two TTM radicals coupled with a molecular bridge. The coupling of adjacent localized spin sites, through a transfer energy *t*, is usually described by the Mott–Hubbard model, where the excitation gap is the electrostatic charging energy for double occupancy of a spin site, termed the Hubbard *U*. The coupling *t* sets up antiferromagnetic exchange, *J* = 2*t*^2^/*U* (refs. ^35,36^). Although the Hubbard *U* is generally considered the energy barrier for carrier transport, we show here that it also sets the energy for the spin singlet photoexcitation. The strength of *t* can be controlled by the nature of the bridging unit, and we have recently reported strong coupling using cyclopentadithiophene units to give strong infrared absorption^37^. Here, we have tuned the interradical transfer energy to a low value, of order a few millielectron volt, using a meta-linked fluorene unit that maintains alternant symmetry, as shown in Fig. 1a.

## Results

Photoexcitation in the spin triplet manifold is constrained to similar non-bonding to π/π* ‘bandgap’ excitations as for the monoradicals, but in the singlet manifold, the lowest energy singlet exciton involves electron transfer from one of the radical non-bonding orbitals to the other, to form a zwitterionic exciton, as illustrated in Fig. 1b,c. It is the Hubbard *U* that sets the energy of the singlet exciton, and for this molecule, its energy is red-shifted from the triplet exciton. This differentiation of zwitterionic singlet from covalent triplet is critical.

Measurements presented here were carried out in dilute solutions (100 µM solutions of toluene) or in spin-coated thin films of polystyrene where the diradical concentration was ≤0.1 wt%. Polystyrene serves as a non-polar host in which the diradical can be dispersed uniformly. We find it necessary to keep these diradical molecules well separated through dilution to prevent intermolecular energy and spin transfers. At these concentrations, the aggregation effects are unimportant (Supplementary Information Section 12). The samples for PL-detected magnetic resonance (PLDMR) were prepared by doping diradicals at 1 nM concentration into 1,3,5-trichlorobenzene crystals.

**Fig. 2:**
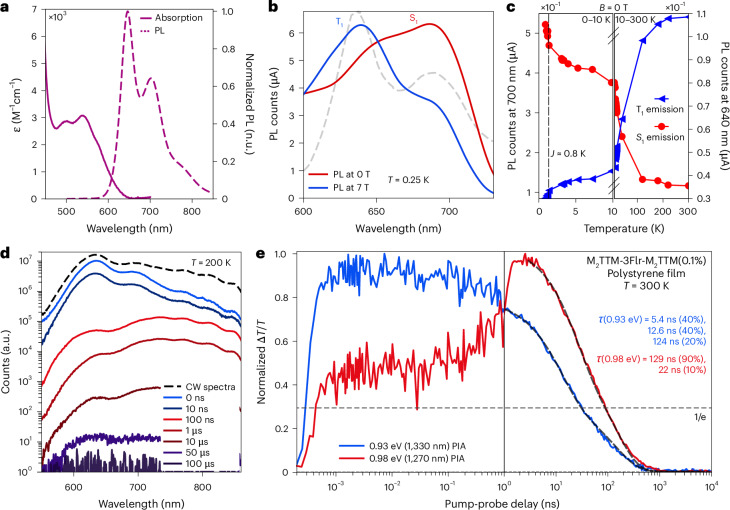
Photophysics of the luminescent diradical. **a**, The absorption and PL spectra for M_2_TTM-3Flr-M_2_TTM. The spectra were acquired under ambient conditions following a 532 nm excitation in a 100 μM toluene solution. n.u., normalized units. **b**, The magnetic-field-dependent PL spectrum of the M_2_TTM-3Flr-M_2_TTM diradical recorded at 0 (red) and 7 T (blue) at 0.25 K. The steady-state PL spectrum at 300 K is also show for reference (grey, dashed) **c**, Zero-field temperature-dependent PL at 640 (blue triangles) and 700 nm (red circles) from 300–0.6 K. The break point in the graph is 10 K. **d**, The time-resolved and continuous-wave PL for M_2_TTM-3Flr-M_2_TTM at 200 K. CW, continuous wave. **e**, The kinetic traces of the PIA features at 520/1,330 nm (blue) and at 610/1270 nm (red) from the transient absorption spectra and the associated fits (dashed lines) for a multiexponential decay model. The exponential decay constants and weights are quoted in the figure. The PL data for all plots were obtained from a M_2_TTM-3Flr-M_2_TTM(0.1%):polystyrene doped polystyrene film following a 532 nm excitation pulse at a fluence of 6 μJ cm^−2^. Source data

Figure 2a shows the absorption and PL spectrum of 100 mM toluene solution of M_2_TTM-3Flr-M_2_TTM. We note that this diradical has approximate alternant symmetry that imposes electron-hole symmetry^38,39^ so that the lowest absorption band has similar but opposing contributions from π → non-bonding and non-bonding → π* transitions. The diradical shows efficient PL, red-shifted from the monoradical (Supplementary Fig. 6), with a peak emission at 640 nm and PLQE above 90%. We note that there is a second peak in the PL near 700 nm, which, very importantly, is not just a vibronic overtone but is due to a spin-singlet excited state, while the 640 nm feature is due to a spin triplet excited state. The distinction between these two emission bands is most easily seen in magnetic-field-dependent PL, measurements at low temperatures, as shown in Fig. 2b. In brief, at 0.25 K, the ground state is a spin singlet (with emission at 700 nm) but with an applied magnetic field (above 0.6 T), the ground state switches to the triplet and the emission at 640 nm is from the triplet exciton.

The differentiation between the singlet and triplet excitons is at the core of our results. The triplet exciton is red-shifted from the monoradical by 0.1 eV, (Supplementary Fig. 4b), but the energy of the zwitterionic singlet exciton is given by a Hubbard *U* and is further red-shifted. We observe that the energy of the singlet PL (700 nm, 1.8 eV) matches the difference in energy between the electrochemical potentials for reduction at −1.2 V (versus Fc/Fc^+^) to the anion and for oxidation at 0.6 V to the cation, shown in Supplementary Fig. 4. The origin of high PLQEs in some of the radical donor–acceptor systems has been explored by Ghosh et al.^40^. In this model, electron-hole separation to vibrationally decoupled molecular fragments in the zwitterionic singlet exciton is responsible for the high PL yield in spite of the long radiative lifetime.

Figure 2c shows the temperature-dependent PL intensity in 0.1 wt% spin-coated thin films of the 640 and 700 nm peaks in the 300–0.6 K regime. The 700 nm PL increases while the 640 nm PL decreases at lower temperatures, although the integrated-PL intensity does not change (Supplementary Fig. 13). The slope of the curves changes twice: first at ~120 K and second at ~1.0 K. We first assign the initial slope-change event to the thermal energy of the system falling below the activation energy for rISC and second when it falls below the antiferromagnetic exchange energy of the diradical.

Figure 2d shows time-resolved PL measurements at 200 K on the 0.1 wt% doped polystyrene films. The M_2_TTM-3Flr-M_2_TTM diradical shows emission from the triplet, peaked at 640 nm, with a fast lifetime of 10 ns and slower emission from the singlet, peaked near 700 nm, with a delayed lifetime of 100 ns and beyond. Analysis of emission before and after 50 ns shows about equal contributions from the triplet and singlet spectra (Supplementary Fig. 8). Although the initial decay of the 640 nm feature is fast it shows a temperature-dependent long-lived component, and this slow component has an activation energy around 10 meV (Supplementary Fig. 12). We consider the slow component of the 640 nm emission to be due to rISC. We note that we are fortunate to observe efficient singlet emission even though it is slow, but nevertheless, we are not able to measure absorption from the spin-singlet ground state, S_0_ to the first spin-singlet excited state, S_1_ because the oscillator strength is low.

Figure 2e shows kinetic traces of the main peaks obtained from transient optical absorption spectroscopy. A detailed report and analysis of transient optical absorption studies is provided in Supplementary Information Section 6, and associated quantum chemical modelling is provided in Supplementary Information Section 10.6. In summary, the photoinduced absorption (PIA) bands near 520 nm (2.05 eV) and 1,330 nm (0.93 eV) are associated with the triplet exciton before charge separation^26^. The PIA bands centred at 610 nm (2.28 eV) and 1,270 nm (0.98 eV) build up over the first 3 ns and are associated to the absorption of the zwitterionic singlet exciton formed after charge separation. The kinetics of the PIA bands associated with the exciton before and after charge separation are in agreement with the kinetics of the 640 and 700 nm PL peaks, respectively. The triplet and singlet PIA features cross over before 1 ns; this can be attributed to the intersystem crossing (ISC) process.

**Fig. 3:**
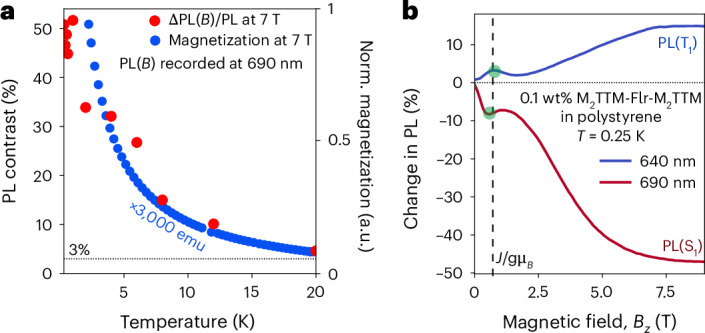
Magneto-optic response. **a**, The temperature dependence of the MPL contrast at 7 T (red) for the 700 nm emission compared with the temperature dependence of the magnetization at 7 T (blue), measured using SQUID magnetometry. Norm., mormalized; emu, electromagnetic unit. **b**, The PL intensity versus magnetic field at 0.25 K, measured on a M_2_TTM-3Flr-M_2_TTM(0.1%):polystyrene doped polystyrene film with 400 nm laser excitation at <1 μJ cm^−2^. Source data

Figures 2b and 3a,b show magneto-optical studies. We find that the 640 and 700 nm emission features are strongly dependent on applied magnetic field (Fig. 2b). In the ground state, the two spins, localized on each of the M_2_TTMs, are weakly antiferromagnetically coupled, as shown in the temperature-dependent magnetization curves in Fig. 3a, with an antiferromagnetic exchange energy near 1 K. This is also captured using PL through the rise in 700 nm and fall in 640 nm emission below 1.2 K, shown in Fig. 2c. In summary, the 700 nm emission dominates at zero field, where only the singlet state is occupied at low temperatures, and as the field is raised above 0.6 T where the Zeeman energy exceeds the exchange energy, the triplet becomes predominantly occupied, and the 640 nm emission dominates. We associate this with a magnetic spin triplet ground state. Figure 3b shows evidence for a resonance where the Zeeman energy equals the ground state exchange energy. In the region close to exchange, we obtain PL spectra that are linear combinations of the singlet and triplet PL which are proportional their relative populations (Supplementary Figs. 17 and 18).

We note that the field and temperature-dependent PL matches the magnetization measurements shown in Fig. 3a. This establishes that the two emissions are associated with the magnetization of the sample arising from the two possible overall spin states, namely singlet at 700 nm and triplet at 640 nm.

We have assembled a photophysical model with the available experimental information in Fig. 1d. We can assume that the initial photoexcitation occurs via fluorene π/π* to non-bonding transitions due to the similarity of the absorption spectrum of the diradical to the monoradical (Supplementary Fig. 6). These new states can be of either singlet or triplet multiplicity, denoted as S_1_ and T_1_ respectively. Extension of the exciton to both radical units will be dependent on molecular geometry, and Fig. 1b illustrates the singlet with zwitterionic character. We have explored this by running extensive time-dependent density functional theory calculations on the M_2_TTM-3Flr-M_2_TTM diradical paying particular attention to the role of dielectric and geometric relaxation (Supplementary Information Section X. The approximate electron-hole symmetry yields a T_1_ triplet exciton with finite oscillator strength (the state involves dominant CT excitation between the fluorene and the TTM) translating into a radiative lifetime of ~20 ns, irrespective of the dielectric constant and in reasonable agreement with experiment. By contrast, the S_1_ singlet state radiative decay is very sensitive to both dielectric and geometric (mostly conformational) relaxation effects. This is because the zwitterionic singlet state acquires finite oscillator strength only through wave function mixing with the singlet counterpart of the ^3^CT excitation, and such mixing varies strongly with their energy separation. Thus, the quasipure zwitterionic state, as formed after full dielectric (at *e* = 2.37 or above) relaxation, has a vanishingly small oscillator strength and a radiative lifetime larger than 1 μs. By relaxing only the optical part of the dielectric response at *n*^2^ ≈ 2 (where *n* is the refractive index of the medium), we instead obtain a hybrid zwitterionic-^1^CT state with radiative lifetime of ~50 ns in the Franck–Condon region and of ~200 ns when relaxing only the high-frequency contribution of the lattice relaxation (thus freezing the conformation of the molecule to that of the ground state), rates bracketing the measured value. Full conformational relaxation brings the S_1_ state further down in energy, resulting again in a radiative lifetime in the microsecond range. We consider this is either at least partly alleviated by steric effects in the solid phase or that thermal fluctuations allow populating higher-energy conformations and/or local dielectric environments where finite zwitterionic-CT mixing occurs.

This zwitterionic singlet exciton is a very unusual excitation for a molecular semiconductor, but its excitation energy, the Hubbard *U*, is a very well recognized quantity for many inorganic oxides. There is extensive literature on Mott–Hubbard models for transition metal oxides, including superconducting cuprates and nickelates^1^. In general, the Hubbard *U* is typically above 2 eV and is larger than other electronic excitations, such as metal to oxygen charge transfer. There has been little consideration about optical absorption and emission to and from the Mott–Hubbard charge transfer state, but there is a recent report on ‘Hubbard excitons’ in SrIrO_4_ (ref. ^41^). For our organic radical systems we are able to bring the Hubbard *U* below the lowest interband exciton, and it is this that allows us to observe the Mott–Hubbard exciton through its PL. The special feature of our studied material is the efficient dual fluorescence from both the triplet and singlet excited states with near unity quantum yield. Matsuoka et al.^21^ report evidence for singlet emission associated with magneto-PL (MPL) in a weakly luminescent diradical system but do not identify the singlet exciton as zwitterionic. We have explored other systems with different bridging units, including carbazole, presented in detail in the Supplementary Information. These show no evidence for PL from singlet excitons. The carbazole-linked system shown in Supplementary Fig. 5 is very similar to that reported by Mizuno et al.^20^ and shows only triplet emission. This is also the case for a recently reported phenyl-linked system^22^.

Since the ground state exchange energy is low (~0.8 K), we expect a 3:1 ratio of triplet:singlet ground states down to low temperatures. We observe however that the ratio of triplet to singlet PL near 300 K is closer to 1:1 (noting that the overall PLQE is >90%) and consider this must arise through ISC from triplet to singlet. The TA evolution shown in Fig. 2e shows this happens beyond 100 ps. We assign the thermally activated delayed emission at 640 nm to rISC, from the S_1_ to the T_1_ state (we note also that the energy of the emitted photon does not capture the full internal energy of the singlet exciton, and we consider there is a conformational energy contribution to the singlet exciton that is not available for photon emission). These observations of competing singlet and triplet excitation and de-excitation pathways, alongside evidence for ISC and rISC, sets up scope for photoexcited spin polarization in the ground state.

**Fig. 4:**
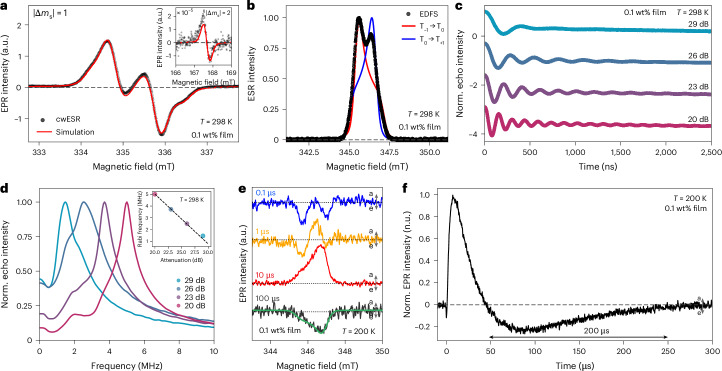
Photoinduced long-lived ground-state spin polarization. **a**, Half-field (left) and full-field (right), dark, continuous-wave *X*-band ESR spectra at 298 K which show the |Δ*m*_s_| = 2 transition in a 0.1 wt% doped polystyrene film (black dots) and the simulation (red line) of all features as a triplet species. **b**, The EDFS spectrum of the diradical. **c**, Rabi oscillations measured at the 345.62 mT transition using different microwave powers. **d**, The Rabi-frequency spectrum plotted for different microwave powers. The inset shows the shift in the peak frequency with decreasing microwave attenuation, and we observe an expected frequency decrease for every 3 dB increase in attenuation. **e**, trESR spectral slices derived at the beginning of each temporal decade (10^2^ to 10^5^ ns) of a 0.1 wt% doped polystyrene film of M_2_TTM-3Flr-M_2_TTM diradical at 200 K. The spectra are obtained at the quoted time point after a 532 nm laser excitation lasting for 5 ns, which repeats at a frequency of 100 Hz. The data highlight the long-lived photogenerated spin-polarized ESR signals persisting beyond 200 μs. The simulation of the 100 μs slice (green solid line) confirms polarization in T_+_. **f**, The kinetic traces of the trESR signal in the 345–348 mT region, which is the kinetics of light-induced spin polarization observed from the ESR signal, for the 0.1 wt% doped polystyrene film at 200 K (black solid lines) of the M_2_TTM-3Flr-M_2_TTM diradical. Norm., normalised; a, absorption; e, emission; n.u., normalized units. Source data

We carried out ground and excited-state ESR measurements, on 0.1 wt% doped polystyrene thin films containing M_2_TTM-3Flr-M_2_TTM (see the Supplementary Information for 50 µM toluene solutions). In the dark, continuous-wave ESR (cwESR) measurements at 298 K (Fig. 4a) shows clear evidence for triplets with full-field |Δ*m*_s_| = 1 transitions which could be simulated with *D* = 30.3 MHz and *E* = 1.6 MHz. Further confirmation of a ground state triplet state is given by the observation of a |Δ*m*_s_| = 2 transition at half field.

Ground state pulsed ESR was performed, and the resulting echo-detected field sweep (EDFS) shows two peaks, due to the T_−1_ → T_0_ and T_0_ → T_+1_ transitions (Fig. 4b). Echo decay measurements at the two peak positions behave similarly; an exponential decay fit (Supplementary Figs. 46 and 47) gives the phase memory time, T_*m*_, of 950 ns. The long T_*m*_ is comparable with that of a perchlorotriphenylmethyl monoradical^42^. Inversion recovery experiments show that the T_1_ (spin-lattice) relaxation time is 31.8 μs. As T_*m*_ is clearly not limited by T_1_, creating a nuclear-spin free environment could further increase the T_*m*_ (ref. ^43^).

One critical component for molecular systems to be utilized in quantum information applications is the ability coherently manipulate the spin state^44^. Rabi nutation experiments shown in Fig. 4c, show the ability of this system to be coherently driven between two states with multiple oscillations seen at room temperature under relatively low microwave powers. We also observe an expected linear shift in the Rabi-oscillation frequency with increasing microwave power, shown in Fig. 4d.

Transient ESR (trESR) was measured with a 532 nm, 5 ns pulsed photoexcitation. Figure 4e shows spectra measured at different times after excitation. At the timescales shown here, we consider the dominant response to arise from non-thermal spin sublevel populations in the electronic ground state formed by the decay of photogenerated excitons. We note here that the triplet luminescence response has an initial decay time of 10 ns. At 100 ns and 1 µs, two emissive features are observed, which we consider arise from sublevel transitions in the triplet state, with preferential population in the T_0_ and T_+_ sublevels. This indicates either preferential filling of the T_0_/T_+_ or depopulation of the T_−_ spin-levels, the latter via ISC in the excited state to the singlet with spin sublevel selective transition probabilities. At 10 µs, the spectra show a broad absorptive feature and beyond 50 µs this ESR feature reverses sign to become emissive and is measurable beyond 200 µs (Fig. 4f). A similar trESR response is found in frozen solution spectra at 150 K, with the only difference being the initial emissive feature to much more pronounced (see Supplementary Figs. 48 and 49 for two-dimensional maps). Note that the 5 ns pulse excitation (100 Hz repetition rate) is shorter than the PL lifetime and precludes spin pumping via re-excitation. A possible mechanism for the sign reversal (Fig. 4e) is spin sublevel selective excited-state depopulation^6,45^. The later time sign reversal shown in Fig. 4e,f is very unusual, and we consider requires spin repopulation from an excited-state reservoir^46^. A possible mechanism to provide this repopulation channel is by rISC from the reservoir of long-lived excited-state singlets which persists to these timescales at 200 K (Fig. 2c and Supplementary Fig. 11). We confirm the long-lived photogenerated spin polarization beyond 50 µs to be due to overpopulation of the ground state T_+_ sublevel by simulation of the late-time trESR spectra where we require a ground state triplet population, *p*(T_i_), distribution of *p*(T_+_) > *p*(T_0_) = *p*(T_−1_) but with the same *D* and *E* parameters as for the cwESR dark spectrum.

**Fig. 5:**
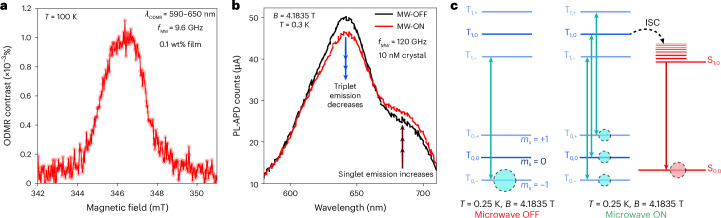
Spin–optical interface in diradicals. **a**, *X*-band ESR conditions (9.7 GHz, 0.346 T) ΔPL/PL >10^−3^% at 100 K. We probe the spin–optical interface using probed using PLDMR spectra. ODMR, optically detected magnetic resonance; fMW, frequency of the applied microwave. **b**, The spectrally resolved PLDMR under conditions at the PLDMR resonance point at 4.1835 T. All PL detected in **a**–**c** and utilized a long-pass filter (>420 nm) to cut-off the laser line. APD, avalanche photo-diode; MW-ON, microwave on; MW-OFF, microwave off. **c**, An illustration of the proposed spin-selective ISC mechanism for the observed PLDMR behaviour. At *B*_z_ > 0.7 T and *T* < 0.8 K the *m*_s_ = −1 sublevel in the ground state triplet is occupied, which has a small ISC probability. *B*z is the static magnetic field applied along the *z*-axis. When microwaves are switched on (right), a microwave-induced Δ*m*_s_ = 1 transition drives population from *m*_s_ = −1 to *m*_s_ = 0 within the ground state. This *m*_s_ = 0 sublevel has a higher probability for ISC in the excited state, which leads to more singlet PL and reduced triplet PL, manifest as a large PLDMR contrast. The samples for **a** were thin films made with 0.1 wt% M_2_TTM-3Flr-M_2_TTM doped into polystyrene, and in **b**, we doped 10 nM of M_2_TTM-3Flr-M_2_TTM into PhCl_3_ crystal. The crystals were prepared using slow evaporation of a solution, which were subsequently washed and polished, as described in Supplementary Information Section 13. Source data

Ground-state spin polarization mediated by a spin–optical interface can be revealed in PLDMR measurements. We explore a range of PLDMR conditions in Fig. 5. The Zeeman energy exceeds *D* above 2.8 mT, so at *X*-band (Fig. 5a) and *D*-band (Fig. 5b), the triplet levels are set by the applied *B*_||z_ fields (Fig. 5c). At 100 K and *X*-band ESR conditions, in Fig. 5a, we see that the PLDMR recorded in the range 590–650 nm shows a contrast >10^−3^%. We report low temperature wavelength-resolved PLDMR under *D-*band conditions in Fig. 5b, showing suppression of the triplet emission and enhanced singlet emission at magnetic resonance. The PLDMR transition is large with ΔPL/PL contrast ~10% and has a narrow 2 mT spectral width, shown in Supplementary Fig. 35, reminiscent of the dark-cwESR spectrum of the diradical. This behaviour arises due to preferential spin-selective ISC from the *m*_s_ = 0 sublevel of the triplet excited state to the S_1_ state, as shown in Fig. 5c, which is turned on by microwave pumping at resonance by the spin sublevel T_−1_ → T_0_ transition in the ground state before photoexcitation (see Supplementary Information Section 5 for more details). The mechanistic insights for the quantum mechanical origins of PLDMR responses from alternant diradicals has also been recently and independently explored by Poh et al.^47^. The size of the PLDMR response is relatively agnostic to microwave and laser excitation power (Supplementary Figs. 35 and 36) but scales with sample magnetization following a Curie-like law, as seen in Supplementary Fig. 34.

## Discussion

In summary, the special feature of our meta-fluorene-bridged diradical is its distinct optically accessible and optically distinguishable triplet and singlet excitons, both with near unity PLQE. This arises because the lowest energy singlet exciton is a bright zwitterionic excitation, with energy set by the onsite Hubbard *U*. This, along with other recent studies^20,21,48^, opens up a new set of molecular semiconductors with a large set of potential areas for application, particularly for quantum sensing and transport. We have demonstrated that we can ‘read’ the ground spin state: singlet or triplet. We have also shown that we can ‘write’—through the Zeeman energy with applied magnetic field and, more broadly, through spin-selective ISC and rISC following optical excitation.

This approach to spin control is relevant to the operational principles of optical qubits such as single-photon-emitting high spin defects in *c*-Si, diamond or *h-*BN. In contrast to such defects, where placement and thus large scale entanglement is difficult^49^ because there is no direct control of defect organization, there is real scope for designed open-shell, highly luminescent, carbon-based molecular spin–optical systems, as exemplified here for the M_2_TTM-3Flr-M_2_TTM diradical. We consider the advances reported here provide a strong and unexpected basis for further developments for optically controlled qubit platforms.

## Methods

### Steady-state optical absorption and PL measurements

Ultraviolet/visible/NIR spectra were measured with a commercially available Shimadzu UV-2550 spectrophotometer and a Shimadzu UV-1800 spectrophotometer. The PL was measured in a home-built setup by providing a continuous-wave excitation at 532 nm using a diode laser. PL is collected in a reflection mode setup after passing photons through a 550 long-pass filter (Thorlabs). The transmitted photons then are collected in a collimating two-lens apparatus and directed into an optical fibre, which supplies the photons into a calibrated grating spectrometer (Andor SR-303i) and finally into a Si-camera where it is recorded. Output spectra are corrected, taking into account the filter transmission and camera sensitivity. The excitation spectra were measured with a commercially available Edinburgh Instruments FS5 Spectrofluorometer system using a xenon lamp light source. When solution samples are studied, 100 mm pathlength Hellma quartz cuvettes were used.

### Cyclic voltammetry

Cyclic voltammetry measurements were carried out on a PalmSens EmStat4S potentiostat in a three-electrode setup using a glassy carbon electrode (3.0 mm diameter) as the working electrode, platinum wire as the counter electrode and freshly activated silver wire as the Ag/Ag^+^ reference electrode. The silver wire was activated by immersing in concentrated HCl solution to remove any silver oxides and other impurities, then rinsed with water and acetone and dried before each measurement. The reference electrode was calibrated against ferrocene/ferrocenium (Fc/Fc^+^) redox couple at the end of each measurement. The Fc/Fc^+^ half-wave potential, *E*_1/2_, was determined at 0.20 V versus Ag/Ag^+^ in THF electrolyte and at 0.50 V versus Ag/Ag^+^ in DCM electrolyte. The supporting electrolyte was 0.1 M solution of Bu_4_NPF_6_ in THF (anhydrous) and in DCM (anhydrous). The scan rate was 0.1 V s^−1^. The electrolyte was bubbled with Ar gas before each measurement to remove any dissolved oxygen. The sample concentration was in the order of 10^−5^ M.

### PLQE

Steady-state PLQE measurements were performed using an integrating sphere. A continuous-wave 405 nm excitation is provided by a 405 nm diode laser with excitation powers of 10–300 mW cm^−2^. A focussed beam of diameter 1,000 μm was used to excite the samples. The emission was directed using an optical fibre in a calibrated grating spectrometer (Andor SR-303i) onto a Si-camera.

### Time-correlated single-photon counting

The studied solution samples are irradiated using an electrically pulsed 405 nm laser using a function generator at a frequency of 20 MHz providing a time resolution of up to 100 ns. The photons emitted from the sample were passed through a 405 nm long-pass filter (Thorlabs) to remove the laser scatter. The subsequently transmitted photons are then collected by a Si-based single-photon avalanche photodiode. The instrument response function was found to be ~0.1 ns in this setup.

### Transient spectroscopy

Transient PL spectra at nanosecond-microsecond timescales were recorded using an electronically gated intensified charge-coupled device (CCD) camera (Andor iStar DH740 CCI-010) connected to a calibrated grating spectrometer (Andor SR-303i). A narrowband noncolinear optical parametric amplifier pumped with a frequency-doubled output of a 1 kHz 800 nm laser pulse from a Ti:sapphire amplifier was used to generate a tunable 250-fs excitation pulse. Suitable long-pass filters (Edmund Optics) were used to prevent scattered laser signals from entering the spectrometer. Temporal evolution of the PL emission was obtained by stepping the intensified CCD delay with respect to the excitation pulse, with a minimum gate width of 5 ns. The raw data were corrected to account for filter transmission and camera sensitivity.

In the same setup, we measured magnetic field effects on the PL and electroluminescence. where the sub-1T magnetic field was generated using an electromagnet from GMW Model 3470 with a 1 cm distance between cylindrical poles, and the field strength was calibrated with a Gaussmeter.

### Transient absorption spectroscopy

Transient absorption experiments were conducted on a setup pumped by a regenerative Ti:sapphire amplifier (Solstice Ace, Spectra-Physics) emitting 100-fs pulses centred at 800 nm at a rate of 1 kHz and a total output of 7 W. Depending on the probed spectral range and timescales, different combinations of optical systems were used.

To collect subnanosecond dynamics in the visible range, frequency-doubled output of the amplifier was used to seed a home-built broadband non-collinear optical parametric amplifier (NOPA) tuned to output 540–750 nm pulses with a beta barium borate mixing crystal (Eksma Optics). Following chirp-correction the white light output was split on a 50/50 beam splitter, focused to below 200 μm and used as the probe and reference beams. Wavelength-tuneable pump pulses were generated in a home-built visible narrowband NOPA. Alternatively, to probe the infrared range the output of the amplifier was used to seed a home-built NOPA tuned to output 1,250–1,700 nm pulses with a periodically poled stoichiometric lithium tantalate mixing crystal. The pump and probe beams were spatially overlapped at the focal point using a beam profiler, with the pump spot diameter at least fivefold larger than the probe. Time resolution was achieved by the introduction of a stepped optical delay (Thorlabs DDS300-E/M) between pump and probe pulses, with a computer-controlled delay stage allowing for maximum delay of 1.9 ns and beam wander of the probe due to changing beam pointing minimized to below 5 μm using a beam profiler. The pump pulses were chopped at 500 Hz to enable shot-to-shot referencing, which accounted for intensity fluctuations in the amplifier. After passing through the sample, the probe and reference beams were dispersed with a grating spectrometer (Shamrock SR-303i, Andor Technology) and simultaneously measured with CCD detector arrays (Entwicklungsbüro Stresing).

To collect subnanosecond dynamics in the ultraviolet range, the output of the amplifier was used to seed a home-built broadband NOPA tuned to output 350–650 nm pulses generated by focusing the 800 nm fundamental beam onto a CaF2 crystal (Eksma Optics, 5 mm) connected to a digital motion controller (Mercury C-863 DC Motor Controller), after passing through a mechanical delay stage. The transmitted pulses were collected with a single-line scan camera (JAI SW-4000M-PMCL) after passing through a spectrograph (Andor Shamrock SR-163).

### Quantum chemical modelling

The excited-state analysis on the diradical M_2_TTM-3Flr-M_2_TTM was carried out at the time-dependent density functional theory level within the Tamm–Dancoff approximation and by resorting to the screening range-separation hybrid approach, as described in earlier works^50^. In particular, the LC-ωhPBE functional^51^ was used in combination with the cc-pVDZ basis set. For such an approach, the *ω* parameter was optimally tuned, where *α* + *β* = 1/*ε*, with *α* and *β* being adjustable parameters and *ε* the dielectric constant of the chosen medium. All the calculations were performed by using the Gaussian16 suit of packages in its revision A.03 (ref. ^52^).

### Global analysis

Global analysis was performed using the sum of weighted components analysis where the spectrum is, at a first approximation, expressed as an *i*-term linear sum of spectral components *φ*_*i*_ (*λ*):

$$f(\lambda ,t)=\sum _{i}{\varphi }_{i}(\lambda ){{\mathrm{e}}}^{-t/{\tau }_{i}}.$$

Each *i*th spectral component is weighted by a time-varying weight prefactor computed from its associated lifetime *τ*_*i*_ given by ${{\mathrm{e}}}^{-t/{\tau }_{i}}$. This equation can be cast into a *n* × *m* matrix using the method described by Dorhliac et al.^53^. This is a matrix consisting of *n*-reduced weight singular vectors (represented by (US)*n*) that can completely reconstruct the experimental data matrix. The matrix formalism equation is then written as

$${\left({{\mathrm{US}}}\right)}_{n}={E(\vec{\tau })}_{n\times m}{X}_{m\times 1},$$

where the time-varying exponential weight matrix is given by $E(\vec{\tau })$ and *X* is the wavelength-dependent weight matrix; the dimensions of each term is given in the subscript. A simulated annealing algorithm^54^ used for global minimization routines is then used to determine which lifetimes *t*_*i*_ can best satisfy the matrix equation. The determined lifetimes from the optimization are then inserted into the matrix equation, and then, it is solved for X by solving the least-square problem^55^

$${\min }_{\tau }{\left|{\left({{\mathrm{US}}}\right)}_{n}-{E(\vec{\tau })}_{n\times m}{X}_{m\times 1}\right|}^{2},$$

### Magnetic susceptibility measurements

The magnetic susceptibility measurements were carried out in a Quantum Design SQUID MPMS3 Magnetometer. The samples were in the form of polycrystalline powders, about 8 mg, which were packed into a diamagnetic capsule.

### MPL measurements

The MPL measurements were carried out on Bluefors dry dilution refrigerator with a superconductive vector magnet from American Magnetics Model. The samples were installed on a gold-coated copper plate and cooled down to 250 mK. The excitation source was a 405 nm (3.06 eV) laser stabilized through a (proportional-integral-differential) PID loop, which was also used to provide power amplitude modulation at 1.2 kHz. A fibre was used to guide the excitation to the sample (excitation power of around 20 nW, 50 µW cm^−2^) and to collect the fluorescence signal. The magnetic field was swept from 0 to 9 T with a ramping speed of 0.1 T min^−1^. The MPL variation at a particular wavelength was collected using an avalanche photodiode (Thorlabs) placed behind a monochromator (HORIBA iHR320) with lock-in detection at the power amplitude modulation frequency.

### High-frequency PLDMR measurements

The measurements were conducted in a Bluefors dry dilution refrigerator with a superconductive vector magnet from American Magnetics Model. The optical setup was identical to the MPL measurement. A quartz whispering mode gallery resonator was used to enhance the AC magnetic field without increasing the power input into the system. The sample was mounted on top of a sapphire cold finger within 1 mm from the surface of the disc resonator near its the perimeter to maximize the AC magnetic field. The *Q*-factor of the resonator was >10^3^, with some reductions attributed to the holder sample and excitation/collection fibre assembly. The mm-wave setup was inspired by the design for the study of the microwave-response from rotons in superfluid helium^56^.

### cwESR

The cwESR spectra were recorded at *X*-band frequencies (∼9.4 GHz) using a laboratory-built ESR spectrometer. The magnetic field was regulated using a Bruker BH15 field controller and monitored with a Bruker ER 035 M NMR Gaussmeter while a Bruker ER 041 MR microwave bridge (with a ER 048 R microwave controller) was used for microwave generation and detection (diode detection). The static magnetic field was modulated at 100 kHz, and lock-in detection was carried out using a Stanford Research SR810 lock-in amplifier in combination with a Wangine WPA-120 audio amplifier. The lock-in detection leads to the derivative spectra. A Bruker ER 4122 SHQE microwave resonator was used. The *g*-factor calibration was additionally done using a N@C60 powder sample at room temperature, with a known *g*-factor (*g* = 2.00204). The inner wall film sample of M_2_TTM-3Flr-M_2_TTM was measured at room temperature with a modulation amplitude of 0.05 mT and a microwave power of 3.2 μW for the main transition and 12.6 mW for the half-field transition. Simulations of the cwESR spectrum were done using the EasySpin toolbox^57^, version 6.0.0-dev.53 and the pepper function. The half-field transition was scaled to account for the difference in microwave power used.

### Pulsed ESR

Pulse ESR was performed at *X*-band (9.7 GHz) using a Bruker ELEXSYS E580 spectrometer, with a Bruker MD5 resonator. All measurements were at room temperature in the dark.

The echo-detected field sweep was performed using the Hahn echo sequence $\frac{\pi }{2}-\tau -\pi -\tau -$ echo, with pulse lengths of *t*_*π*/2_ = 160 ns and *t*_*π*_ = 320 ns, an interpulse delay *τ* of 400 ns, a two-step phase cycle and an echo integration window of 518 ns. The longer pulses were used to supress ESEEM effects.

The Rabi nutation trace was recorded with a three-pulse sequence of $\theta -5\,\upmu {\mathrm{s}}-\frac{\pi }{2}-\tau -\pi -\tau -$ echo, where *θ* is a variable length pulse that is incremented. The nutation trace was recorded at the most intense peak in the EDFS at 345.62mT with different relative B_1_ strengths (attenuations of 29, 26 and 20 dB).

The phase memory time was measured with an echo decay measurement with a pulse sequence $\frac{\pi }{2}-\tau -\pi -\tau -$ echo, where *τ* was incremented from 400 ns to 4.4 μs in steps of 2 ns (*t*_*π*/2_ = 160 ns and *t*_*π*_ = 320 ns). We fit the echo decay with a mono-exponential curve to extract the T_*m*_ time.

The T_1_ (spin-lattice) relaxation time was measured with a saturation recovery sequence, where 20, 24-ns picket fence pulses were used to saturate the transition followed by a delay and a Hahn echo detection sequence with *t*_*π*/2_ = 16 ns and *t*_*π*_ = 32 ns and an integration window of 112 ns. We find the saturation recovery trace can be fit with a mono-exponential curve.

The field calibration was carried out using N@C60.

### trESR

The trESR experiments were performed on a laboratory-built *X*-band (9.7 GHz) continuous-wave spectrometer together with a Bruker MD5 dielectric ring resonator with optical access. Optical excitation at 532 nm was provided using a diode-pumped Nd:YAG laser (Atum Laser Titan AC compact 15 MM) equipped with a second harmonic generator, with an incident pulse energy of ∼400 μJ, a pulse length of 5 ns and operating at a 100 Hz repetition rate. Excitation also included a depolarizer (DPP25-A, Thorlabs) to avoid polarization effects. The temperature was controlled using a Lakeshore 332 temperature controller and a laboratory-built helium flow cryostat. The transients were recorded as the static magnetic field was swept, and continuous-wave microwave irradiation was applied (the samples were measured with a microwave power of 12.6 μW). trESR on M_2_TTM-3Flr-M_2_TTM was measured at 150 K for frozen solution of 0.1 wt% in PMMA in toluene, just below the melting point of toluene, and at 200 K for the inner wall films made of 0.1 wt% in PMMA.

### PLDMR at *X*-band

PLDMR was performed using a home-built optical resonator (based on a dielectric ring resonator), which allows for excitation of the sample and collection of PL in transmission mode). A 365 nm LED (M365L3 Thorlabs) was used for continuous excitation of the sample. The integrated PL from 400 to 650 nm was collected by a silicon detector. (The 365 nm excitation residual was removed via a 400 nm long-pass filter, while we used a 650 nm short pass filter to limit the range of wavelengths seen by the Si detector to be from 400–650 nm). The PLDMR was carried out at *X*-band, and the microwaves were square-wave modulated at a frequency of 967 Hz. The change in PL owing to microwave absorption was monitored at the microwave modulation frequency using lock-in detection (Stanford Research Systems SR830) as the static magnetic field was swept through resonance. The sample was measured at 100 K.

### Inclusion and ethics statement

All collaborators of this study have fulfilled the criteria for authorship required by Nature Portfolio journals have been included as authors, as their participation was essential for the design and implementation of the study. Roles and responsibilities were agreed among collaborators ahead of the research. This work includes findings that are locally relevant, which have been determined in collaboration with local partners. This research was not severely restricted or prohibited in the setting of the researchers and does not result in stigmatization, incrimination, discrimination or personal risk to participants.

## Online content

Any methods, additional references, Nature Portfolio reporting summaries, source data, extended data, supplementary information, acknowledgements, peer review information; details of author contributions and competing interests; and statements of data and code availability are available at 10.1038/s41557-025-01875-z.

## Supplementary information

Supplementary Information Supplementary Figs. 1–80, Tables 1–8 and Discussion.

## Source data

Source Data Fig. 1 Raw data for the molecular orbitals.

Source Data Fig. 2 Raw text files containing unprocessed data for absorption, PL, magneto-PL, transient absorption kinetics and transient PL spectra.

Source Data Fig. 3 Unprocessed data for the magnetic field dependence of the 640 and 690 nm PL features. The temperature dependence of the ratio between the 640 and 690 nm PL features is also provided in a separate file.

Source Data Fig. 4 Data files for cwESR, echo-detected field sweeps, Rabi oscillations, FFT of Rabi oscillations, transient EPR and simulations of the same.

Source Data Fig. 5 Raw data of optically detected magnetic resonance (ODMR) at 100 K and 0.2 K (high frequency, high field) is provided.

## Data Availability

The data that support the findings of this study are available in this Article and its Supplementary Information. All the data for the figures in the main text are also available via the Cambridge Apollo repository at 10.17863/CAM.118148. Source data are provided with this paper.
