# Supplementary material for: Bright triplet and bright charge-separated singlet excitons in organic diradicals enable optical read-out and writing of spin states
Source: Nat Chem. 2025 Jul 29;17(9):1410–7. doi: 10.1038/s41557-025-01875-z (PMC12411279; doi:10.1038/s41557-025-01875-z)
Supplement: Supplementary file 1 — Supplementary Figs. 1–80, Tables 1–8 and Discussion. [file 41557_2025_1875_MOESM1_ESM.pdf]

nature chemistry

Supplementary information

https://doi.org/10.1038/s41557-025-01875-z

Bright triplet and bright charge-separated singlet excitons in organic diradicals enable optical read-out and writing of spin states

In the format provided by the authors and unedited

Table of Contents

I. Molecules Synthesized: 3

I.1 Characterization 3

I.2 Materials. 3

3-Bromo-9H-fluoren-9-one. 4

3-Bromo-9H-fluorene. 4

3-Bromo-9,9-dioctyl-9H-fluorene. 4

3,6-Dibromo-9H-fluorene. 5

3,6-dibromo-9,9-dioctyl-9H-fluorene. 5

I.3 General procedure for the synthesis of αH precursors. 6

3,6-Bis(4-(bis(3,5-dichloro-2',4',6'-trimethyl-[1,1'-biphenyl]-4-yl)methyl)-3,5-dichlorophenyl)-9-(4-hexylphenyl)-9H-carbazole (S1). 7

3-(4-(Bis(3,5-dichloro-2',4',6'-trimethyl-[1,1'-biphenyl]-4-yl)methyl)-3,5-dichlorophenyl)-9,9-dioctyl-9H-fluorene (S2). 7

3,6-Bis(4-(bis(3,5-dichloro-2',4',6'-trimethyl-[1,1'-biphenyl]-4-yl)methyl)-3,5-dichlorophenyl)-9,9-dioctyl-9H-fluorene (S3). 8

I.4 General procedure for the synthesis of π-radicals. 8

3,6-Bis(4-(bis(3,5-dichloro-2',4',6'-trimethyl-[1,1'-biphenyl]-4-yl)methyl)-3,5-dichlorophenyl)-9-(4-hexylphenyl)-9H-carbazolyl radical (M2TTM-3PCz-M2TTM). 9

3-(4-(Bis(3,5-dichloro-2',4',6'-trimethyl-[1,1'-biphenyl]-4-yl)methyl)-3,5-dichlorophenyl)-9,9-dioctyl-9H-fluorenyl radical (M2TTM-3Flr). 10

3,6-Bis(4-(bis(3,5-dichloro-2',4',6'-trimethyl-[1,1'-biphenyl]-4-yl)methyl)-3,5-dichlorophenyl)-9,9-dioctyl-9H-fluorenyl radical (M2TTM-3Flr-M2TTM). 10

I.5 Cyclic Voltammetry 12

I.6 NMR Spectra 13

II. Steady State Optical Properties 21

III. Time Resolved Emission Spectroscopy 24

IV. Magneto-Optical Spectroscopy 30

IV.1 A phenomenological 4-state model: 34

IV.2 Magnetic Field dependent photoluminescence of M_2TTM-3PCz-M_2TTM diradical 37

V. High Frequency Photoluminescence Detected Magnetic Resonance (HF-PLDMR) 42

VI. Transient Absorption Spectroscopy 48

VII. SQUID Magnetometry 51
VIII. Pulsed Electron Spin Resonance 52
IX. Transient Electron Paramagnetic Resonance 54
X. Quantum Chemical Modelling 56
X.1 The monoradical. 56
X.2 The diradical. 57
X.3 Charge-transfer character. 65
X.4 Dielectric and conformational relaxation. 66
X.5 Radiative lifetimes. 71
X.6 Excited-state absorption spectra. 72
XI. Analytical treatment of the diradical 73
XII. Effect of concentration 73
XIII. 10nM diradical doped crystals 75
References 76

I. Molecules Synthesized:

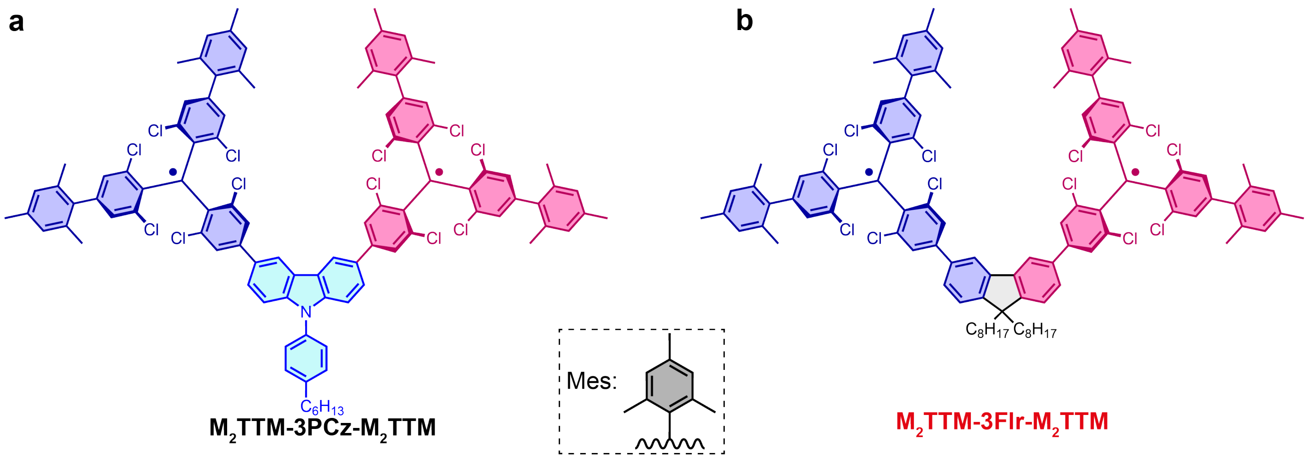

Figure 1: Molecular design of luminescent diradicals. *Diradicals are constructed by coupling mesitylated TTM radicals (M_2TTM) at 3,6-positions (meta) of N-phenylcarbazole (PCz) or dioctylfluorene (Flr). The meta positions lead to synthesis of* ***(a)*** *M_2TTM-3PCz-M_2TTM and* ***(b)*** *M_2TTM-3Flr-M_2TTM.*

I.1 Characterization.

NMR spectra were recorded on a 400 MHz Bruker Avance III HD spectrometer (^{1}H, 400 MHz; ^{13}C, 100 MHz) and a 500 MHz Bruker Avance III Smart Probe Spectrometer (^{13}C, 125 MHz). Chemical shifts are reported in δ (ppm) relative to the solvent peak: chloroform-*d* ($CDCl_3$: ^{1}H, 7.26 ppm; ^{13}C, 77.16 ppm) and dichloromethane-d_2 (CD_2Cl_2: ^{1}H, 5.32 ppm; ^{13}C, 53.84 ppm). High resolution mass spectra were obtained by the Mass Spectrometry service at Yusuf Hamied Department of Chemistry, University of Cambridge. Flash chromatography was carried out using Biotage® Isolera™ Four System and Biotage® SNAP/Sfär Silica flash cartridges.

I.2 Materials.

Preparation of 4',4'''-((2,4,6-trichlorophenyl)methyl)bis(3',5'-dichloro-2,4,6-trimethyl-1,1'-biphenyl) radical (M_2TTM), 2-(4-(bis(3,5-dichloro-2',4',6'-trimethyl-[1,1'-biphenyl]-4-yl)methyl)-3,5-dichlorophenyl)-4,4,5,5-tetramethyl-1,3,2-dioxaborolane (M_2TTM-Bpin), 3,6-dibromo-9-(4-hexylphenyl)-9*H*-carbazole, 3-(4-(Bis(3,5-dichloro-2',4',6'-trimethyl-[1,1'-biphenyl]-4-yl)methyl)-3,5-dichlorophenyl)-9-(4-hexylphenyl)-9*H*-carbazolyl radical (M_2TTM-3PCz) are described in our recent publications (*1*) (*2*). 2-Bromo-9,9-dioctyl-9*H*-fluorene was purchased from Merck, while other reagents, catalysts and (anhydrous) solvents were purchased from Merck, Fluorochem, Alfa Aesar and Acros Organics and used as received. Preparation of other materials are described below.

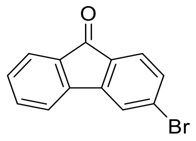

3-Bromo-9H-fluoren-9-one. Adapted from a literature procedure (*3, 4*), to a flask equipped with a dropping condenser open to air was added KOH (70 g) which was dissolved in water (180 mL). 3-Bromophenanthrene-9,10-dione (5.00 g, 17.4 mmol, 1 equiv.) was added to the mixture making a red suspension. $KMnO_4$ (12.38 g, 78.4 mmol, 4.5 equiv.) was added in 1 g portions during 2 h at room temperature. After the additions, the mixture was heated to 110 °C for 2 h, and then cooled to room temperature. The mixture was diluted with DCM and extracted three times with water. The organic phase was dried over anhydrous $MgSO_4$ and filtered through celite. After drying *in vacuo*, yellow solid was collected. The product was used directly in the next reaction without further purification (3.58 g, 79%).

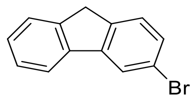

3-Bromo-9H-fluorene. Adapted from a literature procedure (*3, 4*), to a flask equipped with a dropping condenser open to air was added 3-bromo-9*H*-fluoren-9-one (3.00 g, 11.6 mmol, 1 equiv.) and diethylene glycol (35 mL). The mixture was heated at 80 °C and hydrazine monohydrate (2.5 mL, 52.1 mmol, 4.5 equiv.) was added slowly. After stirring at 80 °C for 1 h, temperature was raised to 100 °C and the mixture was stirred another 12 h. During this time, all starting material dissolved and the reaction mixture turned transparent. Solution of KOH (3.90 g, 69.5 mmol, 6 equiv.) in water (10 mL) was added slowly to the reaction and temperature was further raised to 130 °C. After 2 h, the mixture was cooled to room temperature and diluted with DCM. The mixture was extracted three times with water and the organic phase was dried over anhydrous $MgSO_4$. Solvent was removed and the crude product was purified with column chromatography over silica gel using hexane as eluent. After drying *in vacuo*, the target compound was collected as an off-white solid (0.65 g, 23%). ^1H NMR (400 MHz, CD_2Cl_2) δ 7.93 (s, 1H), 7.76 (d, J = 8.5 Hz, 1H), 7.57 (d, J = 5.3 Hz, 1H), 7.42–7.34 (m, 4H), 3.86 (s, 2H). ^{13}C NMR (100 MHz, CD_2Cl_2) δ 144.16, 143.76, 142.60, 142.01, 129.77, 127.87, 127.07, 126.95, 125.43, 123.39, 120.47, 120.17, 36.98.

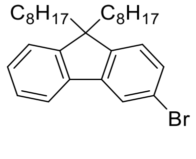

3-Bromo-9,9-dioctyl-9H-fluorene. Adapted from a literature procedure (*3, 4*), to an oven-dried flask equipped with a dropping condenser and under an Ar atmosphere was added 3-bromo-9*H*-fluorene (0.50 g, 2.0 mmol, 1 equiv.) and 1-bromooctane (1.4 mL, 8.2 mmol, 4 equiv.) and anhydrous THF (15 mL). After dissolving the starting material, the mixture was cooled to 0 °C on an ice bath and 1M solution of *t*-BuOK in

anhydrous THF (4.5 mL, 4.5 mmol, 2.2 equiv.) was added dropwise to the reaction. The mixture changed colour quickly from colourless to red, and then cloudy white. The mixture was allowed to warm to room temperature. After stirring at room temperature for 20 h, the mixture was diluted with hexane and extracted three times with water. The organic phase was dried over anhydrous $MgSO_4$ and solvent was removed. The crude product was purified with column chromatography over silica gel using hexane as eluent. After drying *in vacuo*, the target compound was collected as a colourless oil (0.84 g, 88%). ^{1}H NMR (400 MHz, CD_2Cl_2) δ 7.84 (d, J = 1.9 Hz, 1H), 7.70–7.65 (m, 1H), 7.42 (dd, J = 8.0, 1.9 Hz, 1H), 7.38–7.31 (m, 3H), 7.24 (d, J = 8.0 Hz, 1H), 2.00–1.91 (m, 4H), 1.23–1.02 (m, 20H), 0.82 (t, J = 7.1 Hz, 6H), 0.63–0.52 (m, 4H). ^{13}C NMR (100 MHz, CD_2Cl_2) δ 151.36, 150.04, 143.84, 140.17, 130.04, 128.20, 127.30, 124.93, 123.43, 123.20, 120.87, 120.28, 55.43, 40.50, 32.19, 30.34, 29.63, 29.58, 24.14, 23.01, 14.27, 14.25.

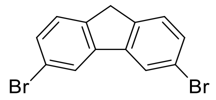

3,6-Dibromo-9H-fluorene. Adapted from a literature procedure (*5*), to an oven-dried flask equipped with a dropping condenser and under an Ar atmosphere was added 3,6-dibromo-9*H*-fluoren-9-one (1.25 g, 3.7 mmol, 1 equiv.) and diethylene glycol (50 mL). The suspension was degassed by bubbling with Ar gas for 30 min before hydrazine monohydrate (0.90 mL, 18.0 mmol, 5 equiv.) was added. The reaction mixture was heated to 100 °C and stirred for 15 h. A solution of potassium hydroxide (1.14 g, 20.0 mmol, 5.4 equiv.) in water (3 mL) was added dropwise and then the reaction temperature was raised to 130 °C. After stirring for 2 h, the reaction mixture was allowed to cool to room temperature and was diluted with water (100 mL) causing precipitation of the organic material. The suspension was filtered, and the precipitate was dissolved in DCM. Water was added, the layers separated, and the aqueous layer was extracted two times with DCM. The combined organic phases were washed with brine, dried over anhydrous $MgSO_4$ and solvent was removed. The crude product was purified with column chromatography over silica gel by gradually increasing the eluent polarity from petroleum ether to 40vol% DCM in petroleum ether. After drying *in vacuo*, the target compound was collected as a yellow solid (0.94 g, 77%). ^{1}H NMR (400 MHz, $CDCl_3$) δ 7.89 (d, J = 1.7 Hz, 2H), 7.47 (dd, J = 8.0, 1.7 Hz, 2H), 7.43 (d, J = 8.0 Hz, 2H), 3.82 (s, 2H). ^{13}C NMR (125 MHz, $CDCl_3$) δ 142.6, 142.2, 130.2, 126.5, 123.4, 121.0, 36.3. HRMS Calcd. for $[C_{13}H_9Br_2]^+$: 322.9071. Found: *m/z* = 322.9072.

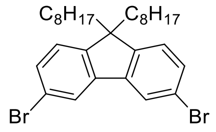

3,6-dibromo-9,9-dioctyl-9H-fluorene. Adapted from a literature procedure (*6*), to an oven-dried flask equipped with a dropping condenser and under an Ar atmosphere was added 3,6-dibromo-9*H*-fluorene (0.94 g, 2.9 mmol, 1 equiv.) and 1-

bromooctane (1.3 mL, 7.5 mmol, 2.6 equiv.). A solution of *t*-BuONa (1.10 g, 11.6 mmol, 4 equiv.) in anhydrous THF (50 mL) was added dropwise to the reaction mixture while stirring at 0 °C. The reaction mixture was allowed to warm to room temperature and stirred for 16 h. Solvent was removed and the mixture was diluted with ethyl acetate (100 mL) and water (100 mL), and the layers separated. The aqueous layer was extracted two times with ethyl acetate and the combined organic phases washed with brine, dried over anhydrous $MgSO_4$, and solvent was removed *in vacuo*. Kugelrohr distillation was carried out to remove excess 1-bromooctane and leave the crude product as a pale brown oil. Further purification was carried out via column chromatography over silica gel using petroleum ether as eluent to produce a colourless oil. The oil was dissolved in DCM and then the solvent removed *in vacuo* again to remove traces of alkane solvents. EtOH (50 mL) was added to the oil and the mixture sonicated. The layers separated naturally and were then left undisturbed for 72 h, during which crystallization occurred to produce white flaky crystals that were filtered from the ethanolic liquor and dried under suction. After drying *in vacuo*, the target compound was collected as a white solid (1.10 g, 69%). ^1H NMR (400 MHz, $CDCl_3$) δ 7.80 (d, J = 2.0 Hz, 2H), 7.46 (dd, J = 8.1, 1.8 Hz, 2H), 7.21 (d, J = 8.1 Hz, 2H), 1.95–1.91 (m, 4H), 1.27–1.03 (m, 20H), 0.85 (t, J = 8.1 Hz, 6H), 0.62–0.54 (m, 4H). ^{13}C NMR (125 MHz, $CDCl_3$) δ 149.7, 141.9, 130.5, 124.4, 123.2, 120.8, 55.0, 40.0, 31.8, 29.9, 29.2, 23.7, 22.6, 14.1. HRMS Calcd. for $[C_{29}H_{41}Br_2]^+$: 547.1575. Found: *m/z* = 547.1581.

I.3 General procedure for the synthesis of αH precursors.

Adapted from our literature procedure (*1*), to an oven-dried microwave vial was added either 3-bromo-, 2-bromo-, 3,6-dibromo- or 2,7-dibromo-functionalized monomer and M_2TTM-Bpin, $Pd(OAc)_2$, SPhos and K_3PO_4, and the vial was subjected to three vacuum/Ar gas refill cycles. Anhydrous 1,4-dioxane was added (10 mL/mmol of bromo- or dibromo-functionalized monomer) and the mixture was degassed by bubbling with Ar gas for 15 min and then heated to 80 °C in an oil bath for 24 h. After cooling to room temperature, the mixture was diluted with hexane and extracted three times with water. The organic phase was dried over anhydrous $MgSO_4$. Solvent was removed and the crude product was purified with column chromatography over silica gel by gradually increasing the eluent polarity from hexane to 5–15vol% DCM in hexane. Finally the solvent was removed and the solids were dried *in vacuo*. Details of individual reactions are provided below.

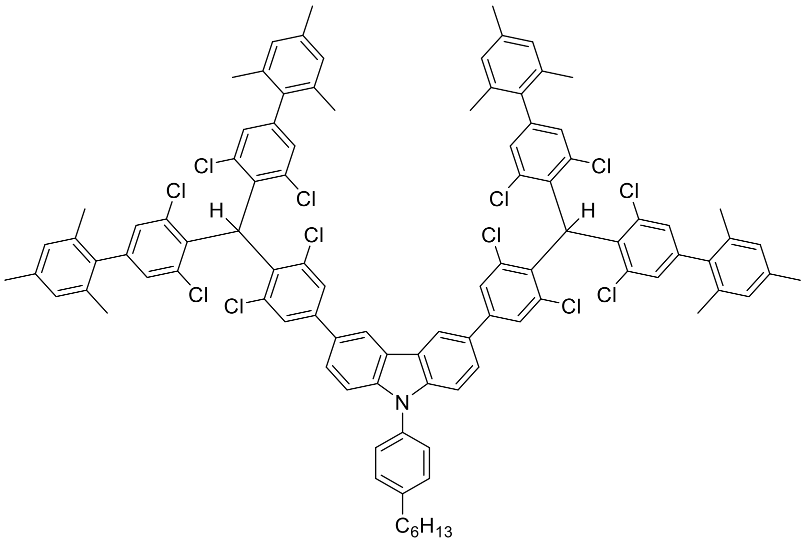

3,6-Bis(4-(bis(3,5-dichloro-2',4',6'-trimethyl-[1,1'-biphenyl]-4-yl)methyl)-3,5-dichlorophenyl)-9-(4-hexylphenyl)-9H-carbazole (S1). 3,6-Dibromo-9-(4-hexylphenyl)-9*H*-carbazole (0.150 g, 0.31 mmol, 1 equiv.), M_2TTM-Bpin (0.603 g, 0.74 mmol, 2.4 equiv.), $Pd(OAc)_2$ (0.0014 g, 0.006 mmol, 0.02 equiv.), SPhos (0.0063 g, 0.015 mmol, 0.05 equiv.), K_3PO_4 (0.252 g, 1.19 mmol, 3.8 equiv.). The product was collected as a white solid (0.418 g, 80%). A smaller scale reaction yielded 0.176 g (84%). ^{1}H NMR (400 MHz, $CDCl_3$) δ 8.43 (d, J = 2.0 Hz, 2H), 7.76 (d, J = 2.0 Hz, 2H), 7.68 (dd, J = 8.6, 1.9 Hz, 2H), 7.62 (d, J = 2.0 Hz, 2H), 7.53–7.42 (m, 6H), 7.19 (dd, J = 3.4, 1.8 Hz, 4H), 7.05 (t, J = 1.8 Hz, 4H), 7.03 (s, 2H), 6.95 (s, 8H), 2.76 (t, J = 7.8 Hz, 2H), 2.34 (d, J = 2.4 Hz, 12H), 2.08 (d, J = 4.0 Hz, 24H), 1.75 (p, J = 7.5 Hz, 2H), 1.50–1.33 (m, 6H), 0.94 (t, J = 7.0 Hz, 3H). ^{13}C NMR (100 MHz, $CDCl_3$) δ 143.07, 142.34, 142.11, 141.66, 137.99, 137.79, 137.67, 137.51, 137.36, 137.00, 136.24, 135.92, 135.86, 135.84, 135.81, 134.76, 134.40, 134.28, 134.06, 131.16, 130.38, 130.10, 129.46, 129.35, 128.67, 128.30, 126.90, 125.33, 123.99, 119.10, 110.74, 50.66, 35.90, 31.90, 31.59, 29.24, 22.79, 21.20, 20.75, 20.72, 20.70, 14.28. HRMS Calcd. for $[C_{98}H_{81}Cl_{12}N]^+$: 1691.26. Found: m/z = 1691.21.

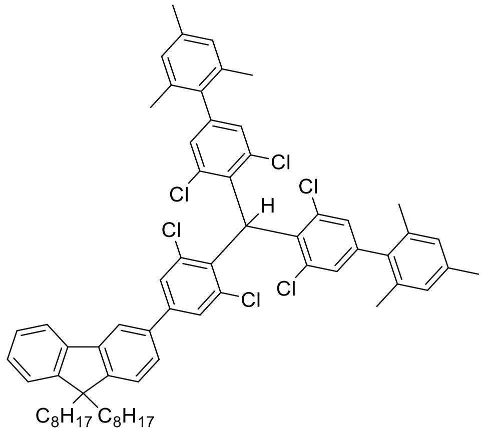

3-(4-(Bis(3,5-dichloro-2',4',6'-trimethyl-[1,1'-biphenyl]-4-yl)methyl)-3,5-dichlorophenyl)-9,9-dioctyl-9H-fluorene (S2). 3-Bromo-9,9-dioctyl-9*H*-

fluorene (0.087 g, 0.18 mmol, 1.5 equiv.), M_2TTM-Bpin (0.100 g, 0.12 mmol, 1 equiv.), $Pd(OAc)_2$ (0.0006 g, 0.002 mmol, 0.02 equiv.), SPhos (0.0025 g, 0.006 mmol, 0.05 equiv.), K_3PO_4 (0.042 g, 0.20 mmol, 1.6 equiv.). The product was collected as white solid (0.090 g, 68%). ^{1}H NMR (400 MHz, CD_2Cl_2) δ 7.95 (d, J = 1.8 Hz, 1H), 7.82–7.74 (m, 2H), 7.62 (d, J = 2.0 Hz, 1H), 7.56 (dd, J = 7.9, 1.8 Hz, 1H), 7.45 (d, J = 7.8 Hz, 1H), 7.41–7.31 (m, 3H), 7.20 (dd, J = 3.3, 1.8 Hz, 2H), 7.06 (dd, J = 4.9, 1.7 Hz, 2H), 7.02 (s, 1H), 6.94 (s, 4H), 2.31 (s, 6H), 2.05 (d, J = 4.4 Hz, 12H), 2.04–1.97 (m, 4H), 1.25–0.99 (m, 20H), 0.81 (t, J = 7.1 Hz, 6H), 0.68–0.55 (m, 4H). ^{13}C NMR (100 MHz, CD_2Cl_2) δ 151.61, 151.44, 142.65, 142.56, 142.51, 140.98, 138.19, 137.90, 137.88, 137.81, 137.55, 137.16, 136.99, 136.39, 136.00, 135.97, 135.92, 134.90, 134.51, 134.45, 131.64, 131.55, 129.84, 129.02, 128.52, 128.50, 127.87, 127.27, 127.23, 126.20, 123.89, 123.42, 120.15, 118.44, 55.48, 51.01, 40.64, 32.18, 30.37, 29.64, 29.58, 24.23, 23.00, 21.17, 20.72, 20.68, 14.24.

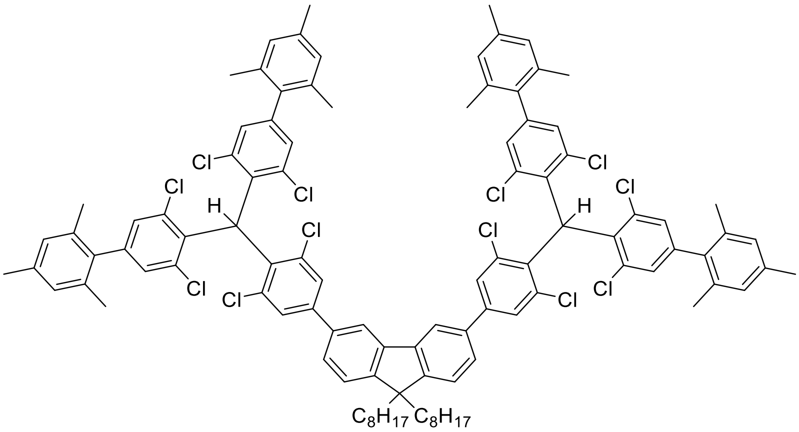

3,6-Bis(4-(bis(3,5-dichloro-2',4',6'-trimethyl-[1,1'-biphenyl]-4-yl)methyl)-3,5-dichlorophenyl)-9,9-dioctyl-9H-fluorene (S3). 3,6-dibromo-9,9-dioctyl-9*H*-fluorene (0.070 g, 0.13 mmol, 1 equiv.), M_2TTM-Bpin (0.228 g, 0.28 mmol, 2.2 equiv.), $Pd(OAc)_2$ (0.0006 g, 0.003 mmol, 0.02 equiv.), SPhos (0.0026 g, 0.006 mmol, 0.05 equiv.), K_3PO_4 (0.095 g, 0.45 mmol, 3.5 equiv.). The product was collected as a white solid (0.217 g, 97%). ^{1}H NMR (400 MHz, $CDCl_3$) δ 7.99 (d, J = 1.7 Hz, 2H), 7.73 (d, J = 2.1 Hz, 2H), 7.62–7.54 (m, 4H), 7.43 (d, J = 7.9 Hz, 2H), 7.19 (d, J = 5.0 Hz, 4H), 7.05 (dd, J = 4.0, 1.8 Hz, 4H), 7.02 (s, 2H), 6.95 (s, 8H), 2.34 (d, J = 2.9 Hz, 12H), 2.08 (d, J = 3.8 Hz, 24H), 2.05–1.98 (m, 4H), 1.25–1.04 (m, 20H), 0.82 (t, J = 7.1 Hz, 6H), 0.73–0.58 (m, 4H). ^{13}C NMR (100 MHz, $CDCl_3$) δ 151.39, 142.14, 142.02, 141.65, 137.99, 137.75, 137.66, 137.52, 137.35, 137.10, 137.00, 136.96, 136.23, 135.91, 135.86, 135.82, 134.75, 134.33, 134.22, 131.16, 129.47, 129.36, 128.83, 128.31, 127.09, 126.33, 123.53, 118.58, 55.32, 50.70, 40.49, 31.92, 30.16, 29.38, 29.34, 24.00, 22.73, 21.21, 20.75, 20.71, 14.22. HRMS Calcd. for $[C_{103}H_{98}Cl_{12}]^{(-H)-}$: 1761.38. Found: m/z = 1761.37.

I.4 General procedure for the synthesis of π-radicals.

Adapted from our literature procedure (*1*), to a microwave vial was added αH precursor (1 equiv.) and the vial was subjected to three vacuum/Ar gas refill cycles. Anhydrous

THF was added to dissolve all starting material followed by addition of anhydrous DMSO in 1:3 (v/v) THF/DMSO ratio making a 0.5 wt% solution of the αH precursor. The mixture was degassed by bubbling with Ar gas for 15 min and the vial was covered from light. In the darkness, 40 wt% Bu_4NOH (aq) (2 equiv. per αH) (bubbled with Ar gas for 15 min prior to use) was added and the mixture was stirred at room temperature for 12 h. *p*-Chloranil (2.5 equiv. per αH) was added and the mixture was stirred another 1 h. The mixture was diluted with hexane, extracted three times with water and the organic phase was dried over anhydrous $MgSO_4$. Solvent was removed and the crude product was purified with column chromatography over silica gel by gradually increasing the eluent polarity from hexane to 5–15vol% DCM in hexane. Finally, the solvent was removed and the solids were dried *in vacuo*. All radicals were stable under ambient air and light in both solution and solid state, but they were stored under inert gas in the dark. Details of individual reactions and deviations from this procedure are provided below.

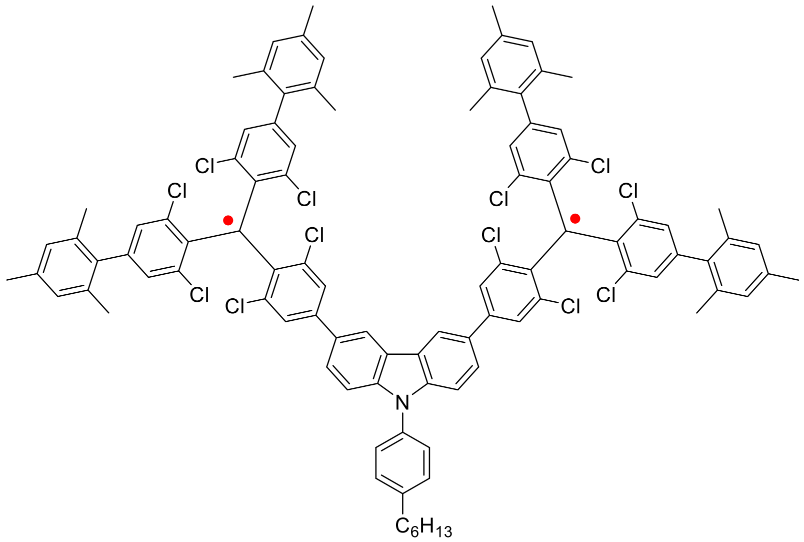

3,6-Bis(4-(bis(3,5-dichloro-2',4',6'-trimethyl-[1,1'-biphenyl]-4-yl)methyl)-3,5-dichlorophenyl)-9-(4-hexylphenyl)-9H-carbazolyl radical (M2TTM-3PCz-M2TTM). Compound S1 (0.360 g, 0.21 mmol, 1 equiv.), 40% Bu_4NOH (aq) (0.57 mL, 0.85 mmol, 4 equiv.), *p*-chloranil (0.261 g, 1.06 mmol, 5 equiv.), THF (18 mL), DMSO (54 mL). The product was collected as a green solid (0.323 g, 90%). A smaller scale reaction yielded 0.091 g (91%). ^{1}H NMR (400 MHz, $CDCl_3$) δ 3.32 (s, 2H), 1.73 (s, 2H), 1.42 (d, J = 27.2 Hz, 6H), 0.99–0.89 (m, 3H). Aromatic protons and mesityl group protons not resolved. HRMS Calcd. for $[C_{98}H_{79}Cl_{12}N]^+$: 1689.25. Found: m/z = 1689.23.

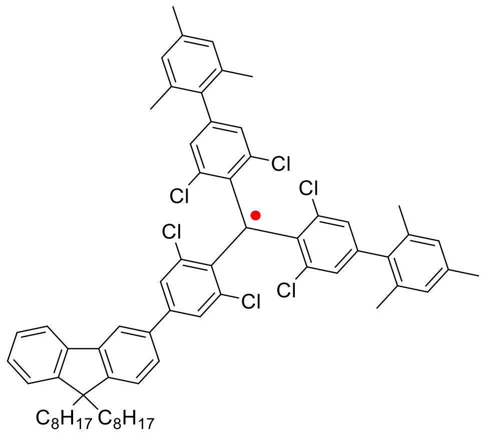

3-(4-(Bis(3,5-dichloro-2',4',6'-trimethyl-[1,1'-biphenyl]-4-yl)methyl)-3,5-dichlorophenyl)-9,9-dioctyl-9H-fluorenyl radical (M2TTM-3Flr). Compound S2 (0.050 g, 0.05 mmol, 1 equiv.), 40% Bu_4NOH (aq) (0.06 mL, 0.09 mmol, 2 equiv.), *p*-chloranil (0.029 g, 0.12 mmol, 2.5 equiv.), THF (2.5 mL), DMSO (7.5 mL). The product was collected as a red solid (0.038 g, 76%). ^{1}H NMR (400 MHz, CD_2Cl_2) δ 1.26–1.03 (m, 20H), 0.81 (t, J = 6.8 Hz, 6H), 0.78–0.61 (m, 4H). Aromatic protons, mesityl group protons and four octyl side chain protons (2 × 2H) not resolved.

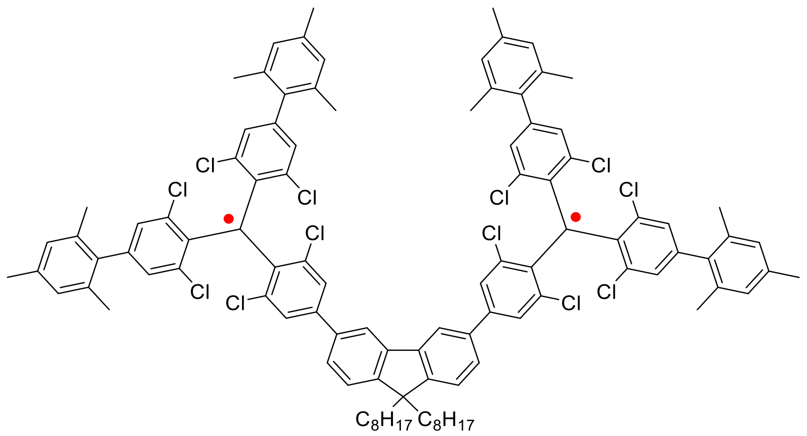

3,6-Bis(4-(bis(3,5-dichloro-2',4',6'-trimethyl-[1,1'-biphenyl]-4-yl)methyl)-3,5-dichlorophenyl)-9,9-dioctyl-9H-fluorenyl radical (M2TTM-3Flr-M2TTM). Compound S3 (0.150 g, 0.09 mmol, 1 equiv.), 40% Bu_4NOH (aq) (0.23 mL, 0.34 mmol, 4 equiv.), *p*-chloranil (0.105 g, 0.43 mmol, 5 equiv.), THF (7.5 mL), DMSO (22.5 mL). The product was collected as a brown-red solid (0.121 g, 81%). ^{1}H NMR (400 MHz, $CDCl_3$) δ 1.25–0.99 (m, 20H), 0.89–0.78 (m, 10H). Aromatic protons, mesityl group protons and four octyl side chain protons (2 × 2H) not resolved. HRMS Calcd. for $[C_{103}H_{96}Cl_{12}]^{(+H)+}$: 1757.38. Found: *m/z* = 1757.38.

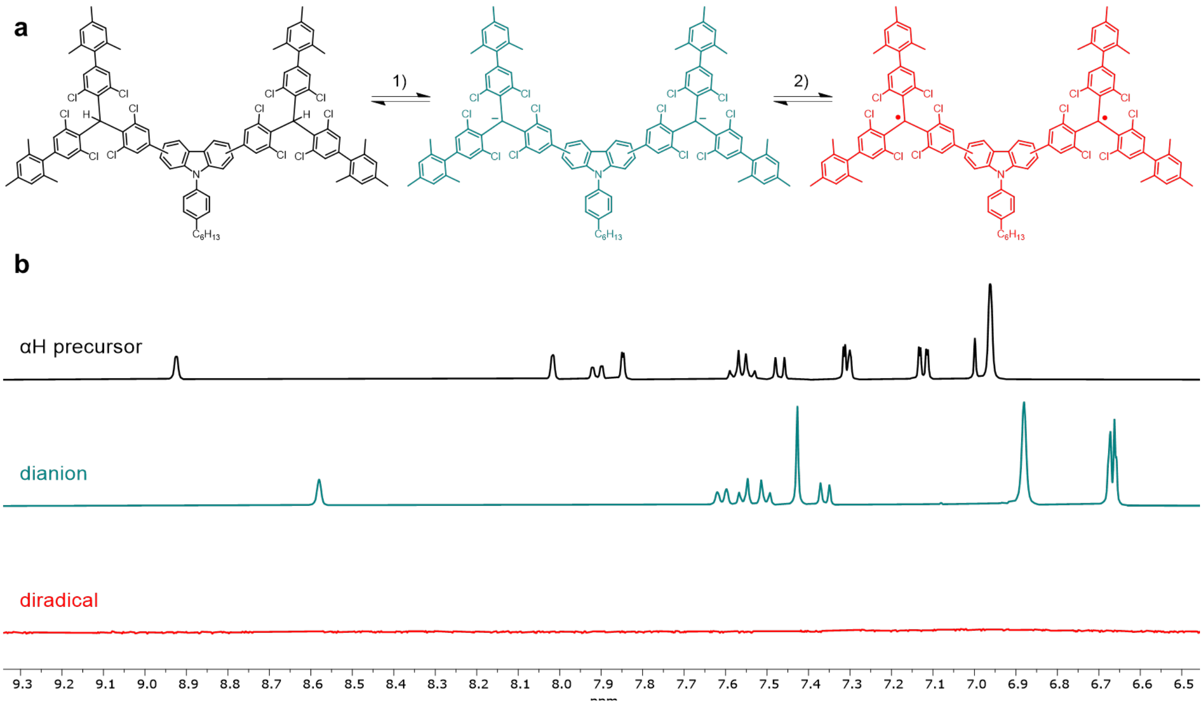

Figure 2: NMR monitoring of synthesis of N-phenyl carbazole bridged diradicals. (a) *Illustration of two-step radical conversion and* ***(b)*** *^{1}H NMR spectra of M_2TTM-3PCz-M_2TTM showing full conversion of αH precursors (black lines) to deprotonated dianionic intermediates (teal lines) and to diradical products (red lines) in DMSO-d_6/THF-d_8 3:1 (v/v). All spectra have been referenced against 1,3,5-trimethoxybenzene (^{1}H, 6.09 ppm) as the internal standard.*

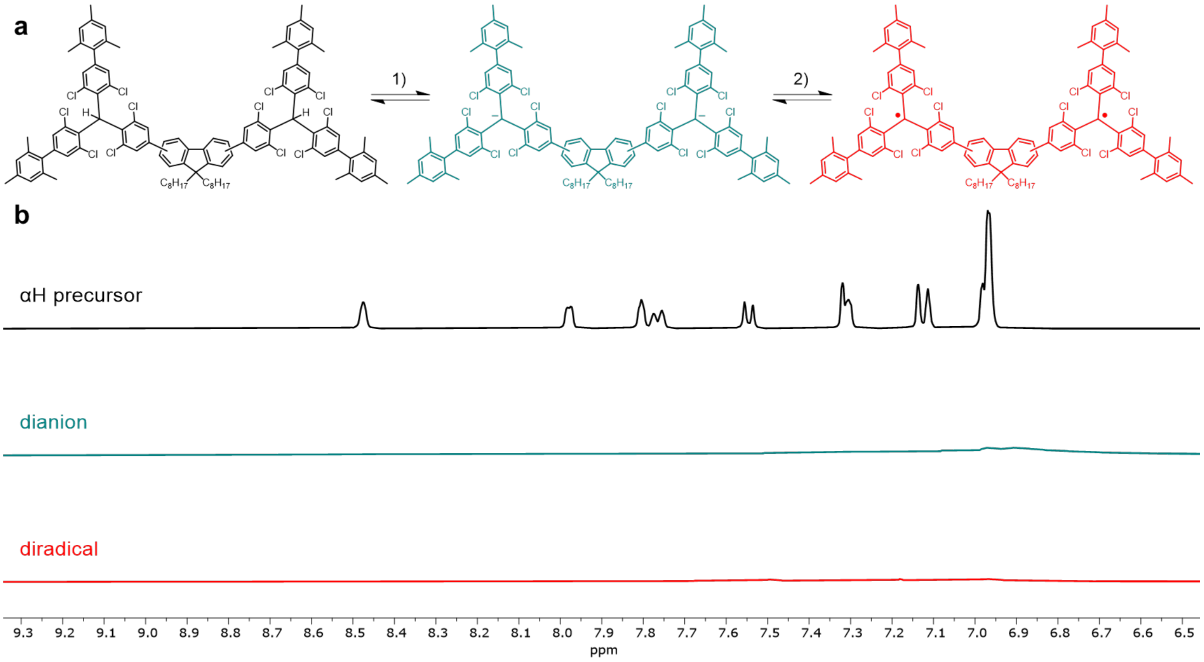

Figure 3: NMR monitoring of synthesis of fluorene bridged diradicals. (a) *Illustration of two-step radical conversion and* ***(b)*** *^{1}H NMR spectra of M_2TTM-3Flr-M_2TTM showing complete conversion of αH precursors (black lines) to deprotonated dianionic intermediates (teal lines) and to diradical products (red lines) in DMSO-d_6/THF-d_8 3:1 (v/v). All spectra have been referenced against 1,3,5-trimethoxybenzene (^{1}H, 6.09 ppm) as the internal standard.*

I.5 Cyclic Voltammetry

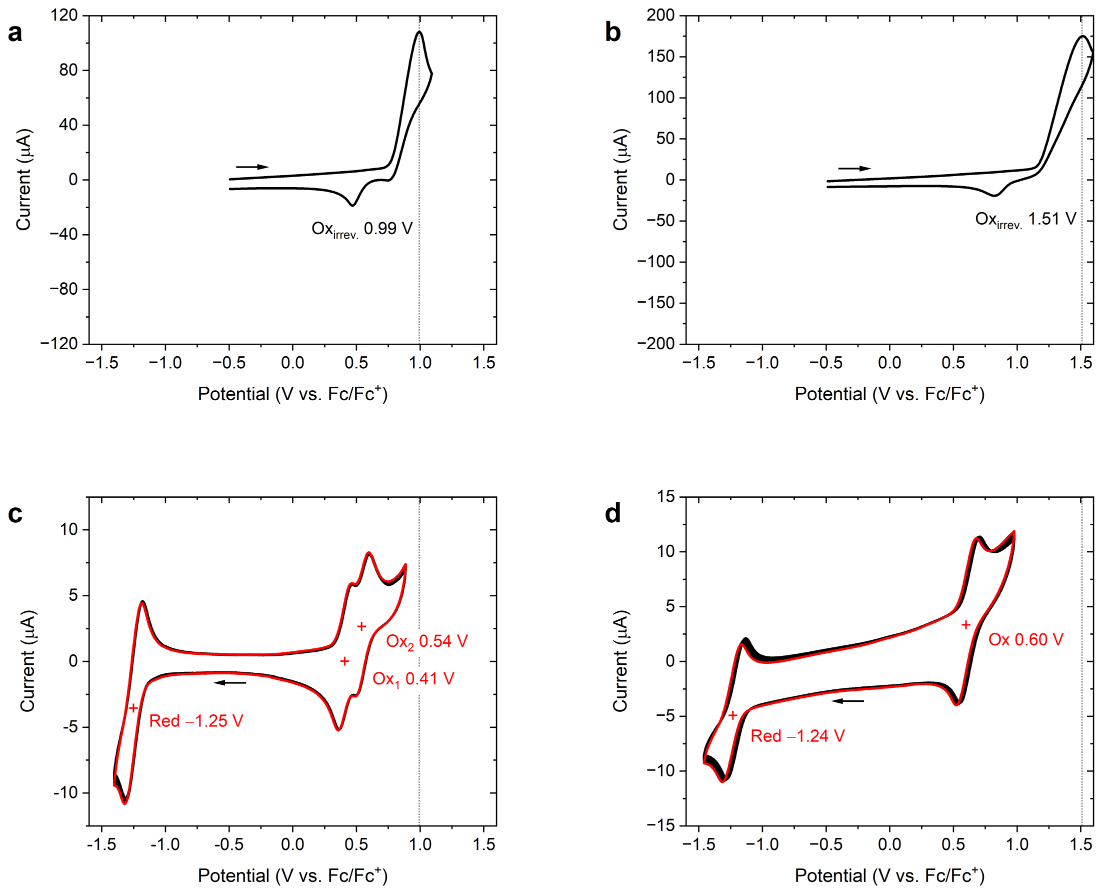

Figure 4: Cyclic voltammetry. *Oxidation scans for **(a)** PCz and **(b)** Flr linkers. The redox reactions of the linkers are interpreted irreversible and the oxidation potentials are reported as peak potentials (grey dashed vertical lines). Ten full redox cycles for meta-linked **(c)** M_2TTM-3PCz-M_2TTM and **(d)** M_2TTM-3Flr-M_2TTM. The reduction and oxidation potentials of the diradicals are reported as half-wave potentials for the first redox cycle (red line) in the cathodic and anodic ranges, respectively. The half-wave potentials are indicated by red plus signs. The dashed vertical lines in **c–d** are guides to the eye only. The arrow indicates the scan direction. The redox reactions in **c–d** are assigned to the two radical centres. We note that M_2TTM-3Flr-M_2TTM shows one reversible oxidation wave whereas in the other diradical oxidation is split into two waves separated by 0.1 V. This can be due to charge transfer from the linker (in **c**) that, after oxidation of one radical site, changes the oxidation potential of the other site. These effects are not observed in **d** where the two radicals are weakly coupled through conjugation and the linker is a weak electron donor. Accordingly, the reduction is observed as one reversible wave in all diradicals. The supporting electrolyte was 0.1 M solution of Bu_4NPF_6 in DCM (for **a,b**) and THF (for **c,d**). The scan rate was 0.1 V s^{-1}.*

Note: *The difference between oxidation and reduction potentials in Figure I.4.d correspond to the energies of the cation and anion at the singly-occupied SOMO site, this is similar to the Hubbard U in a Mott-Hubbard insulator. In M_2TTM-3Flr-M_2TTM this difference in energy is 1.84 eV. The zwitterionic singlet exciton PL is centred at 1.78 eV which is in excellent agreement to the CV measurement of the charging energy for the system.*

I.6 NMR Spectra

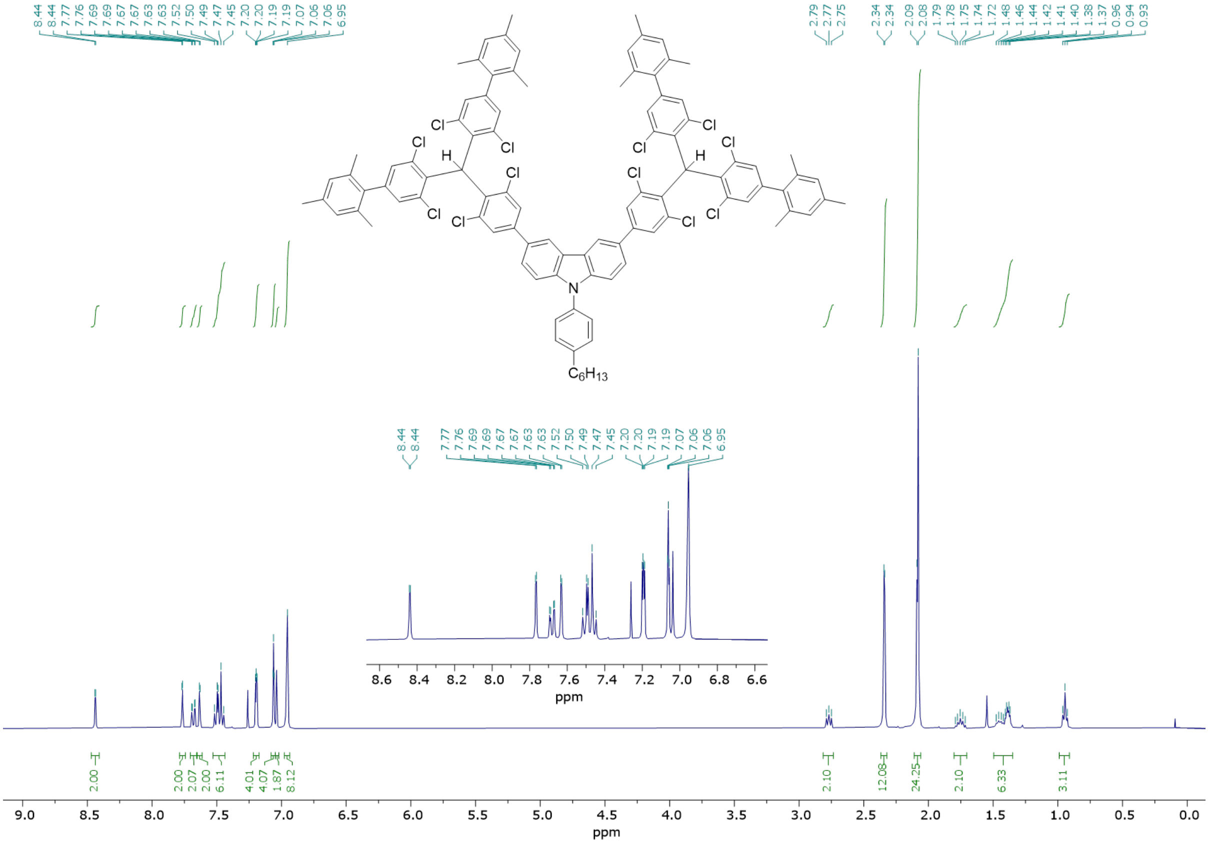

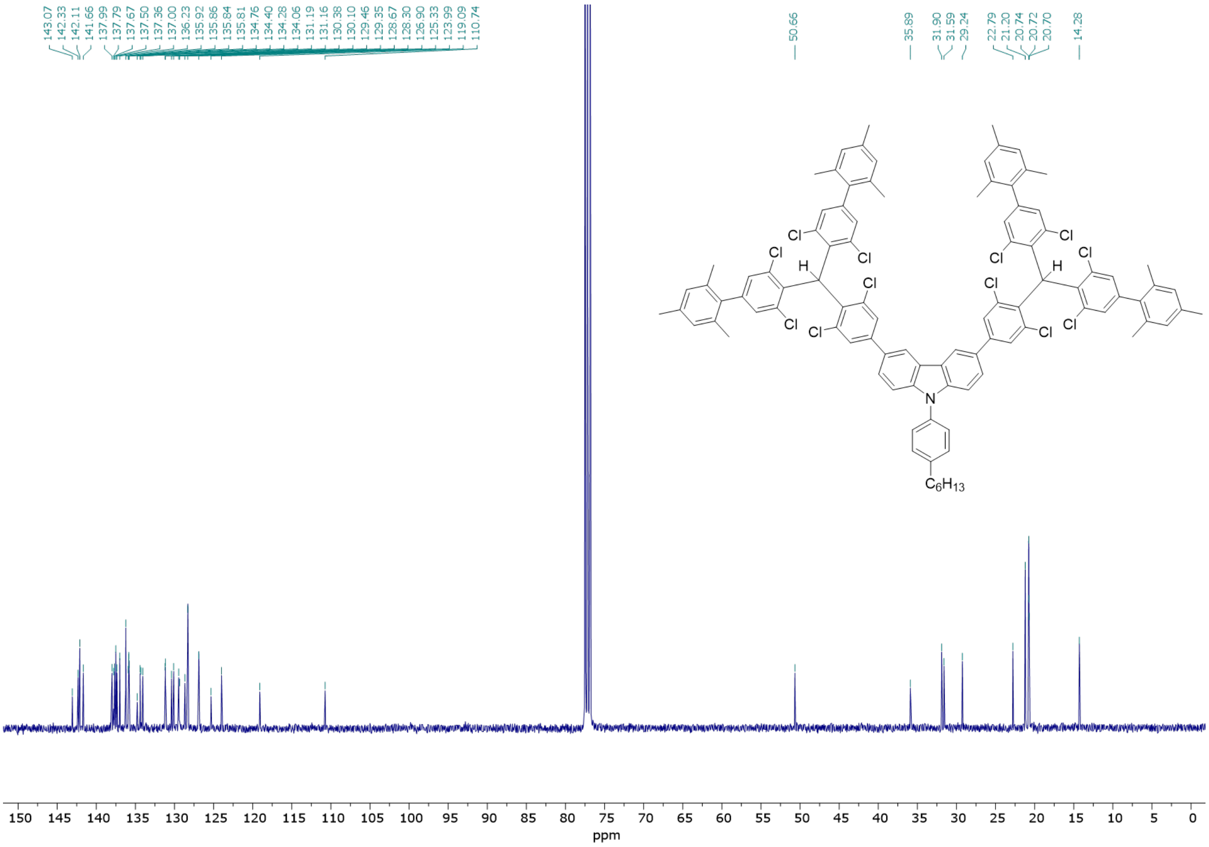

COSY

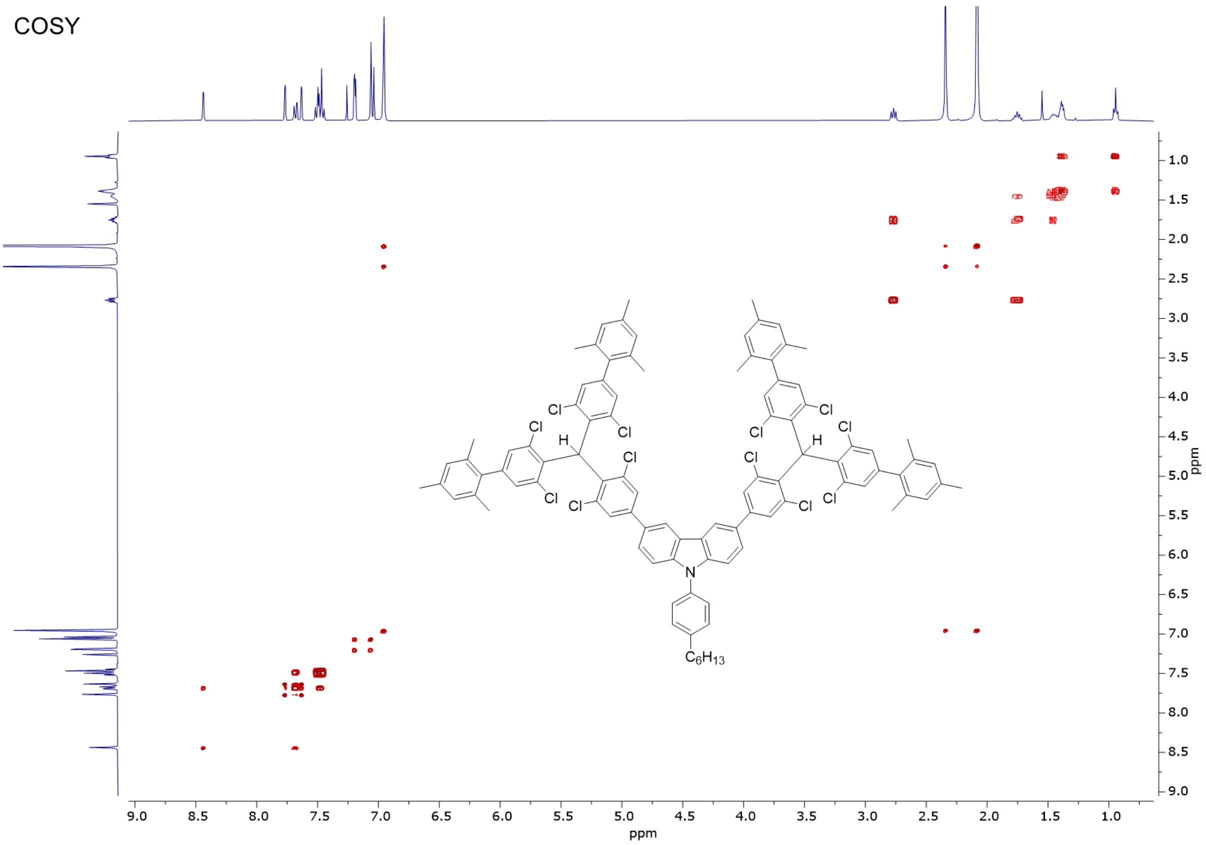
Cl
H
C6H13
N
ppm
9.0
8.5
8.0
7.5
7.0
6.5
6.0
5.5
5.0
4.5
4.0
3.5
3.0
2.5
2.0
1.5
1.0

HSQC

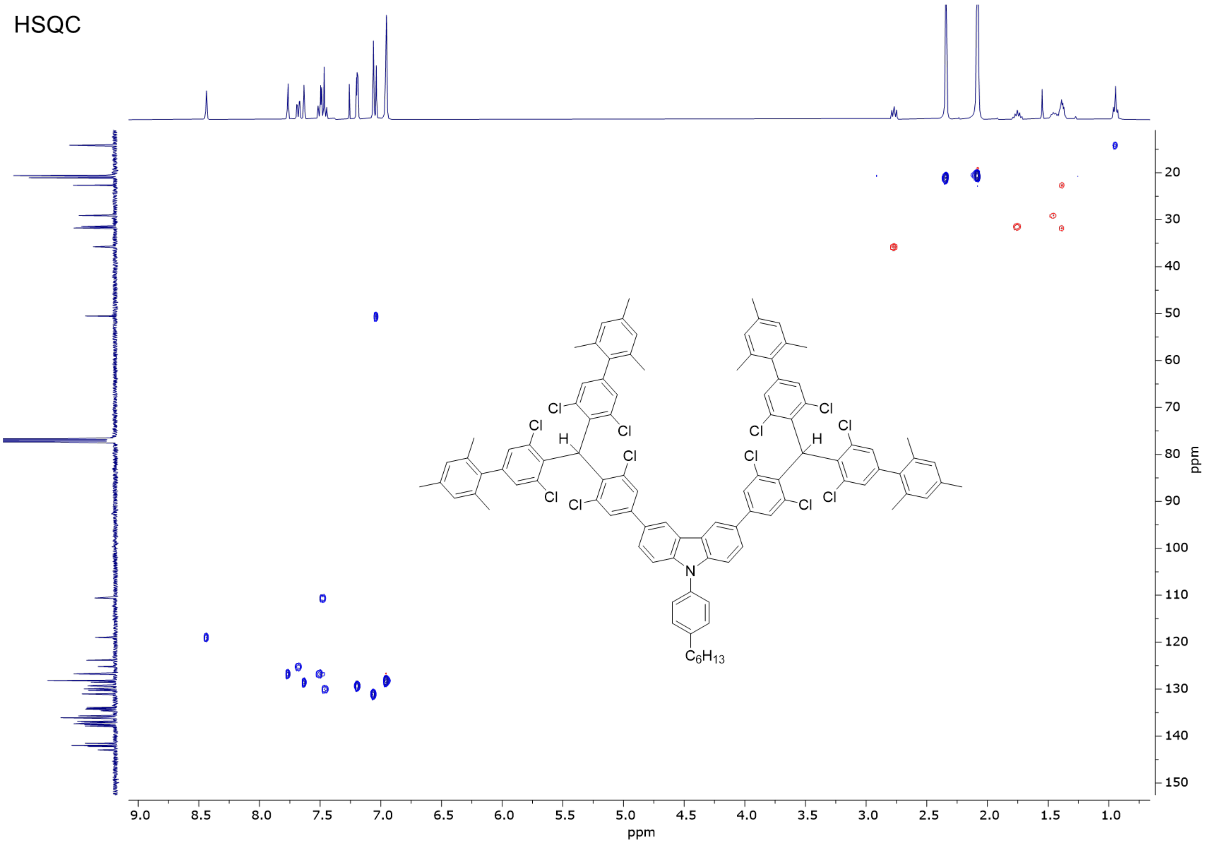
Cl
H
C6H13
N
ppm
9.0
8.5
8.0
7.5
7.0
6.5
6.0
5.5
5.0
4.5
4.0
3.5
3.0
2.5
2.0
1.5
1.0
20
30
40
50
60
70
80
90
100
110
120
130
140
150

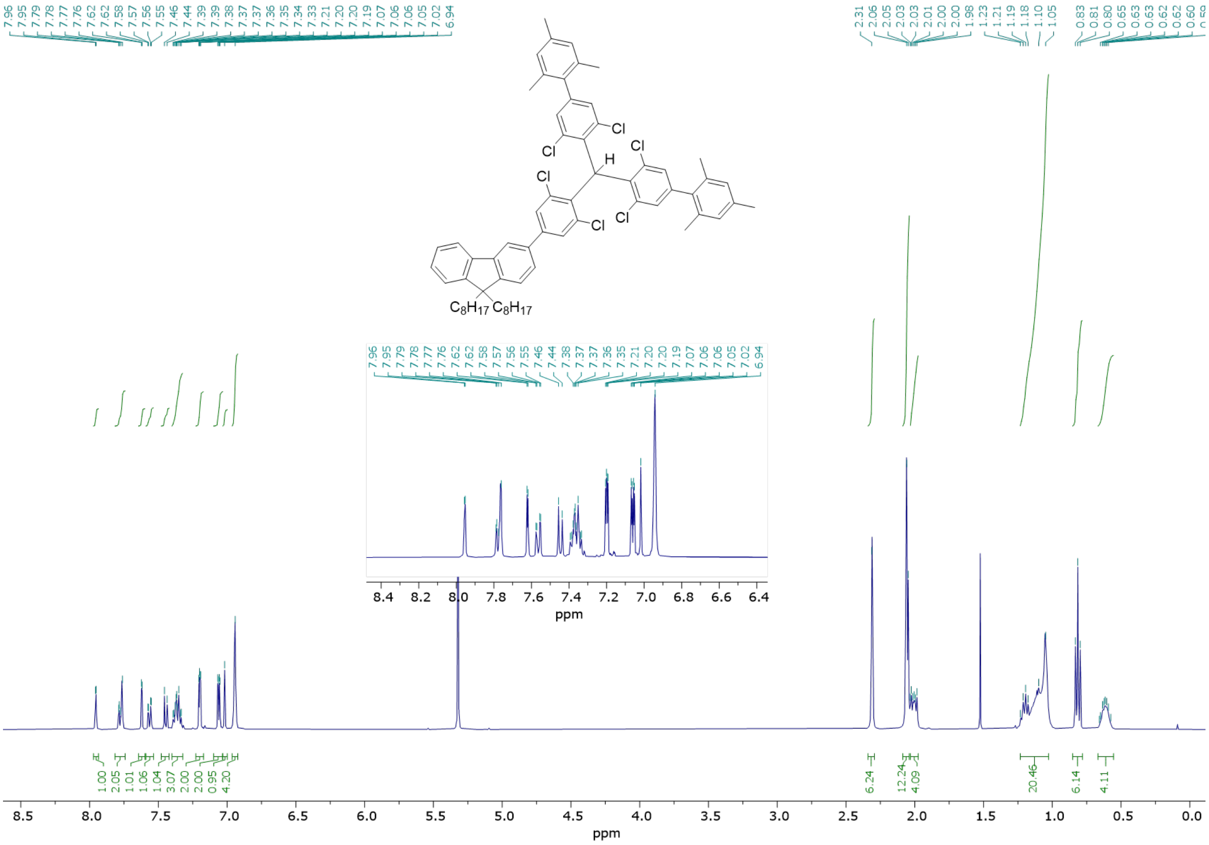

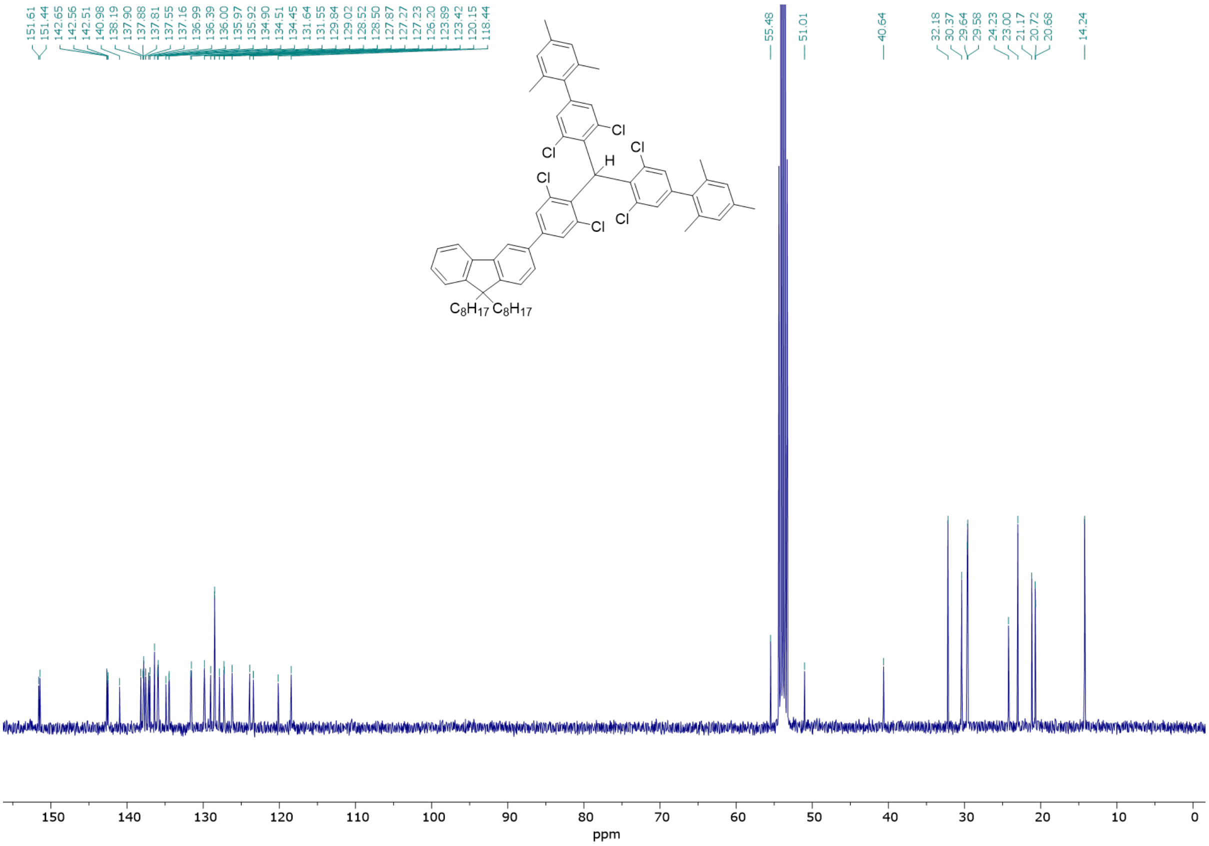
151.61
151.44
142.65
142.56
142.51
140.98
138.19
137.90
137.88
137.81
137.55
137.16
136.99
136.39
136.00
135.97
135.92
134.90
134.51
134.45
131.64
131.55
129.84
129.02
128.52
128.50
127.87
127.27
127.23
126.20
123.89
123.42
120.15
118.44
55.48
51.01
40.64
32.18
30.37
29.64
29.58
24.23
23.00
21.17
20.72
20.68
14.24
Cl
H
C_8H_{17} C_8H_{17}
ppm

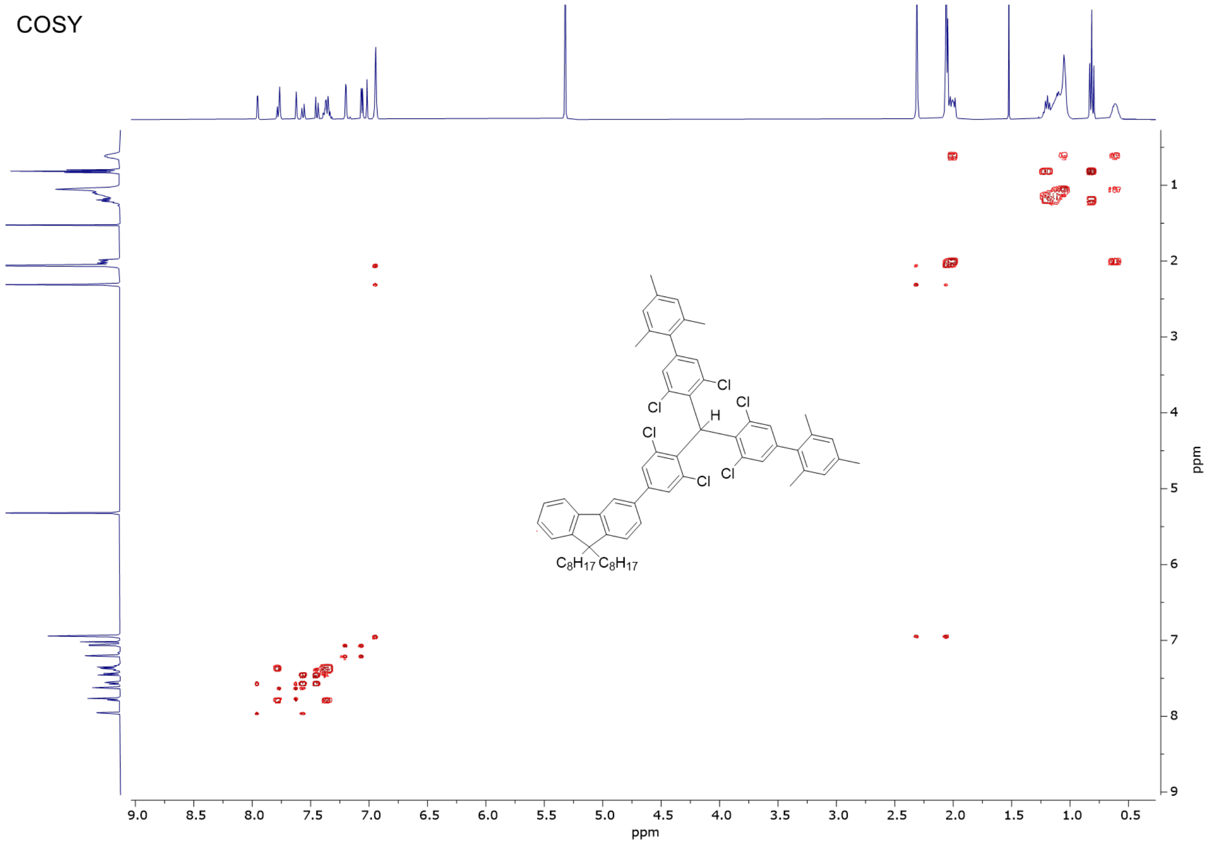
COSY
Cl
H
C_8H_{17} C_8H_{17}
ppm
ppm

HSQC

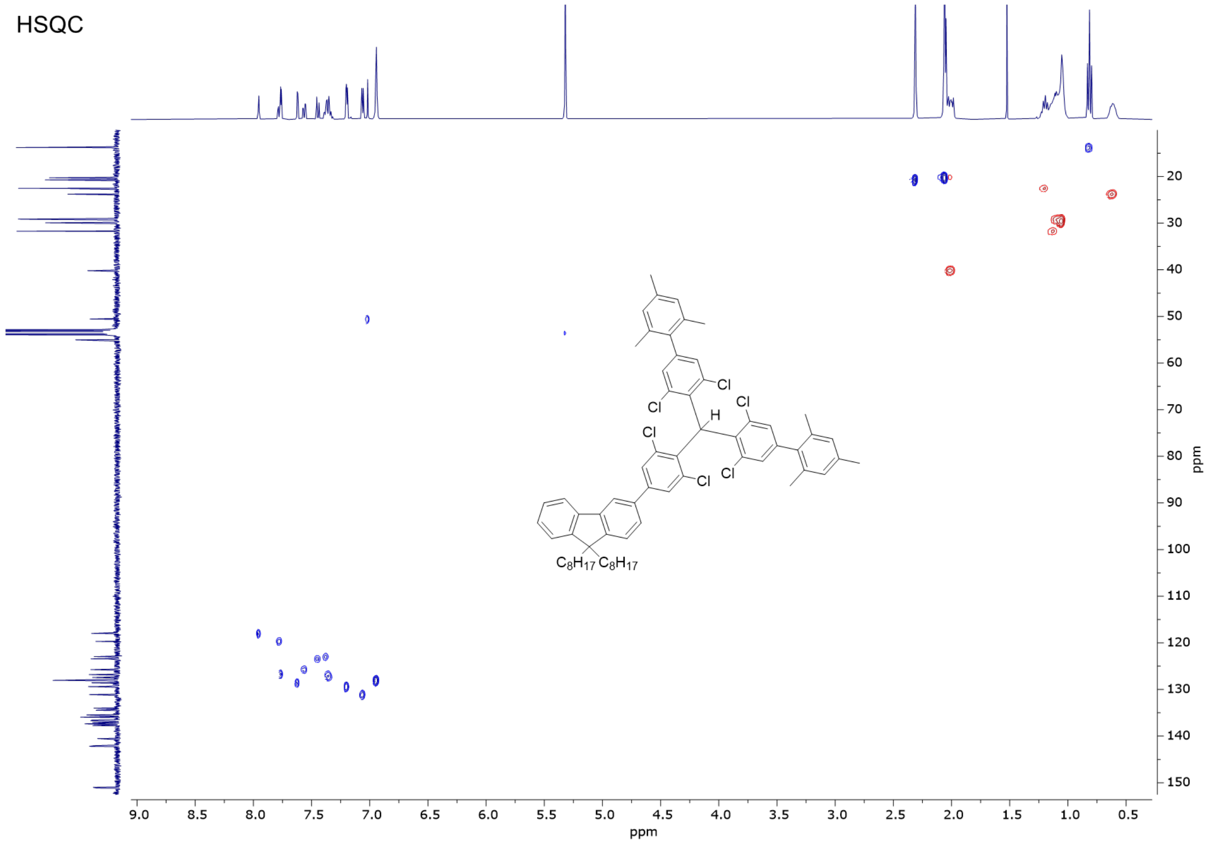

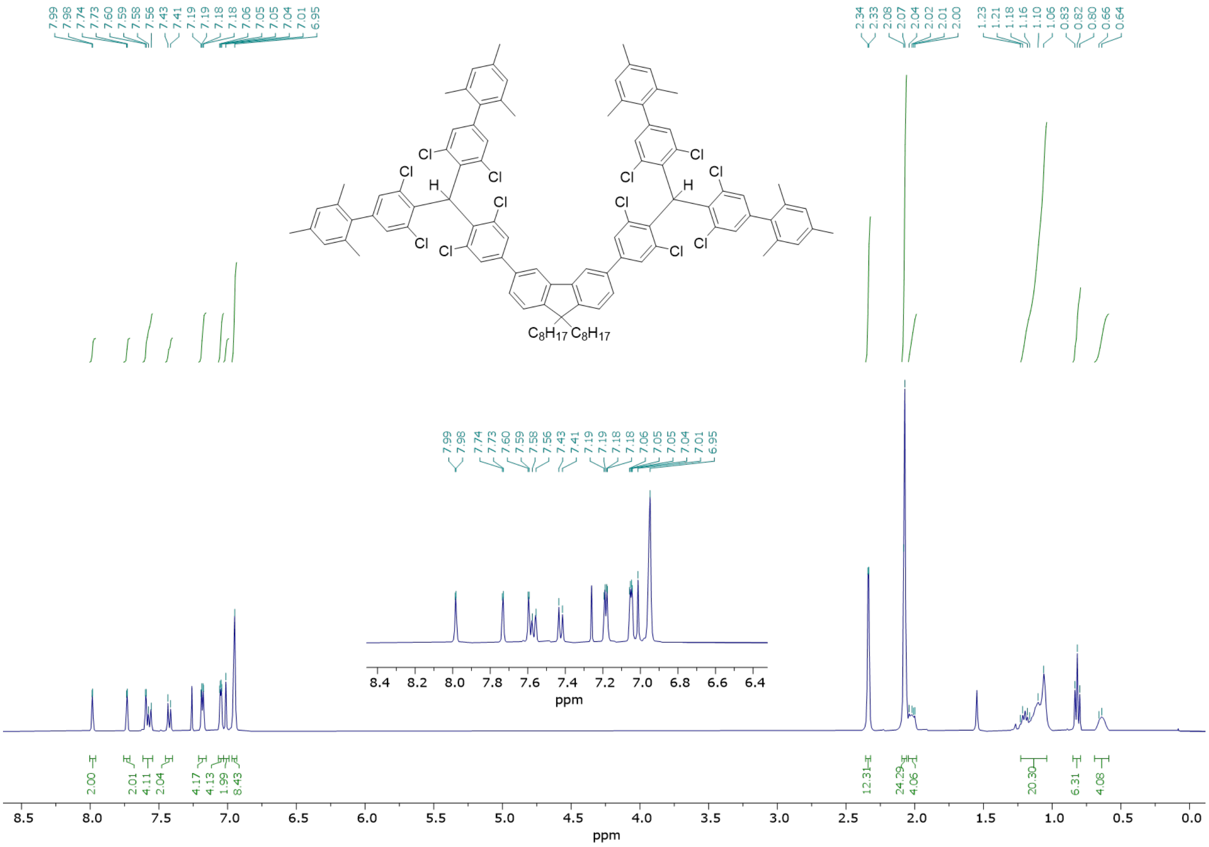

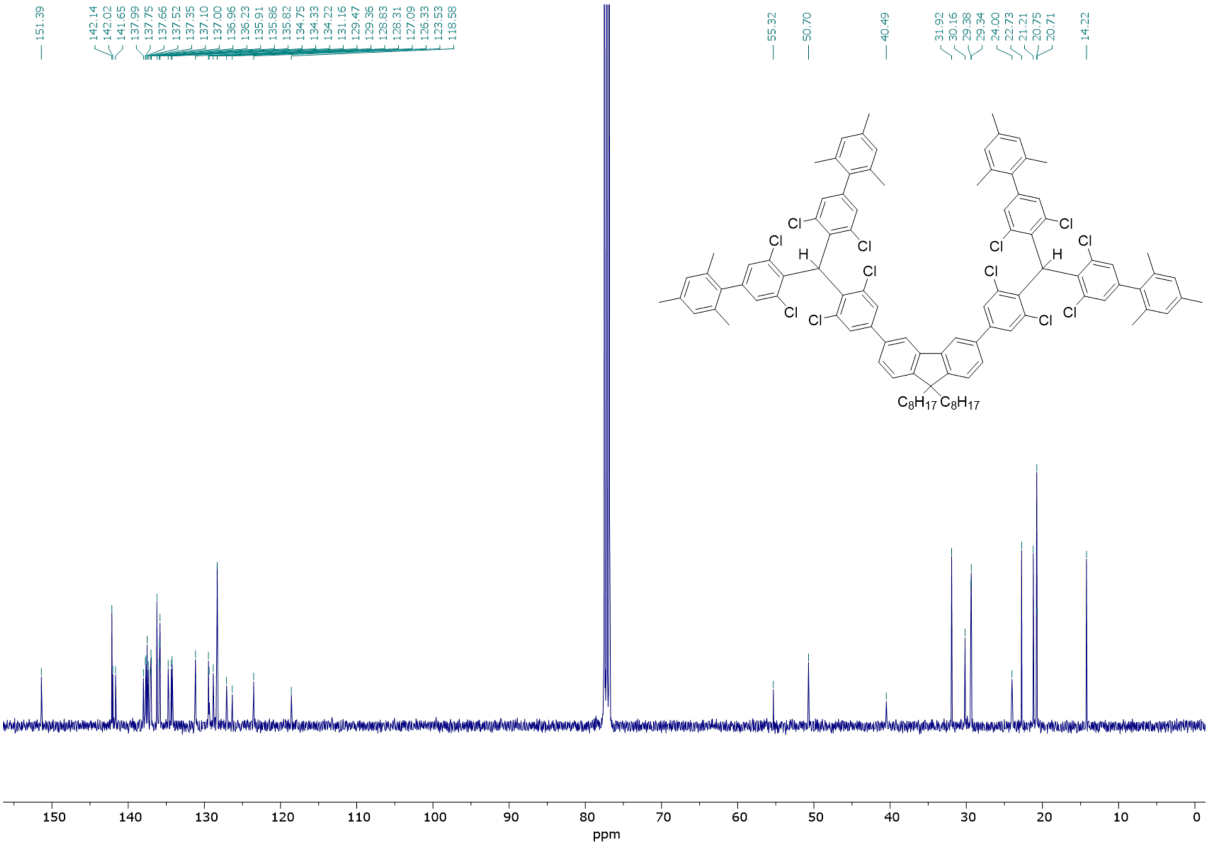
151.39
142.14
142.02
141.65
137.99
137.75
137.66
137.52
137.35
137.10
137.00
136.96
136.23
135.91
135.86
135.82
134.75
134.33
134.22
131.16
129.47
129.36
128.83
128.31
127.09
126.33
123.53
118.58
55.32
50.70
40.49
31.92
30.16
29.38
29.34
24.00
22.73
21.21
20.75
20.71
14.22
Cl
H
C8H17 C8H17
ppm

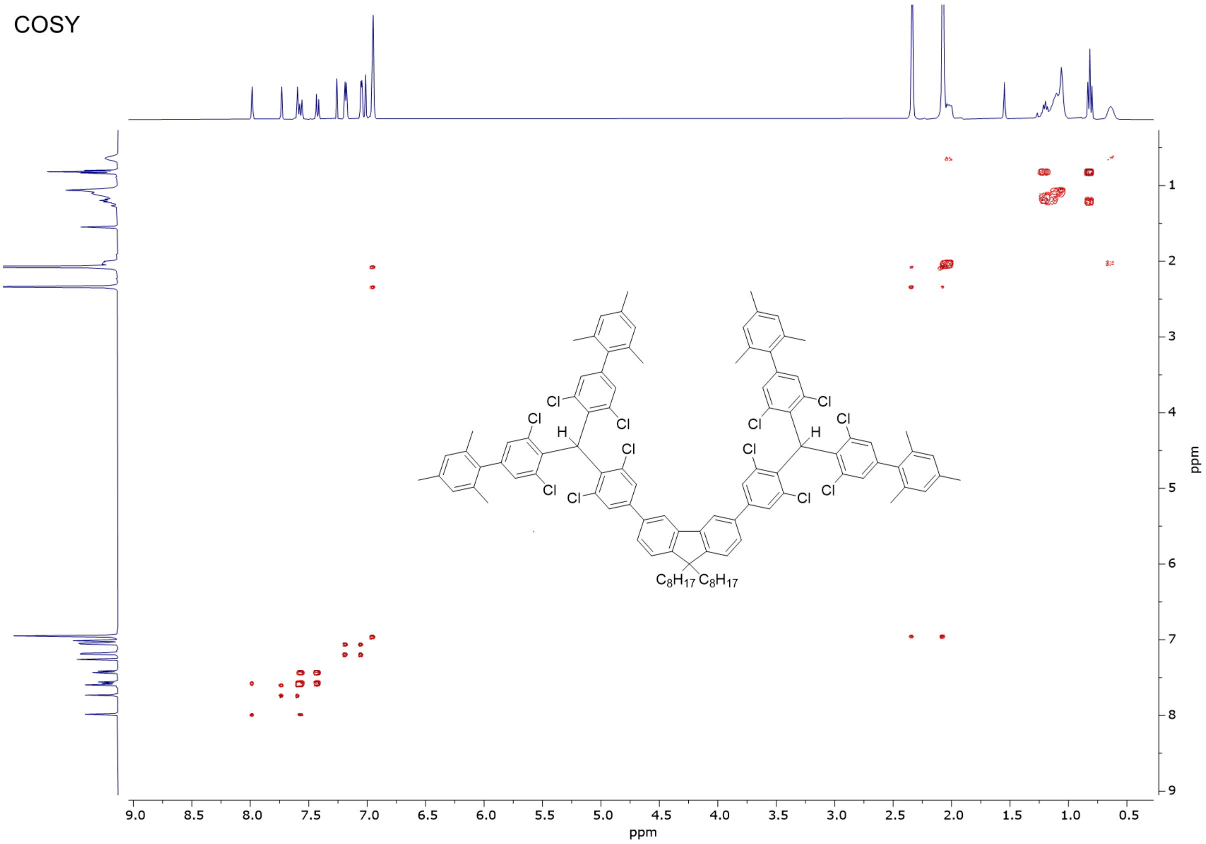
COSY
Cl
H
C8H17 C8H17
ppm
ppm

HSQC

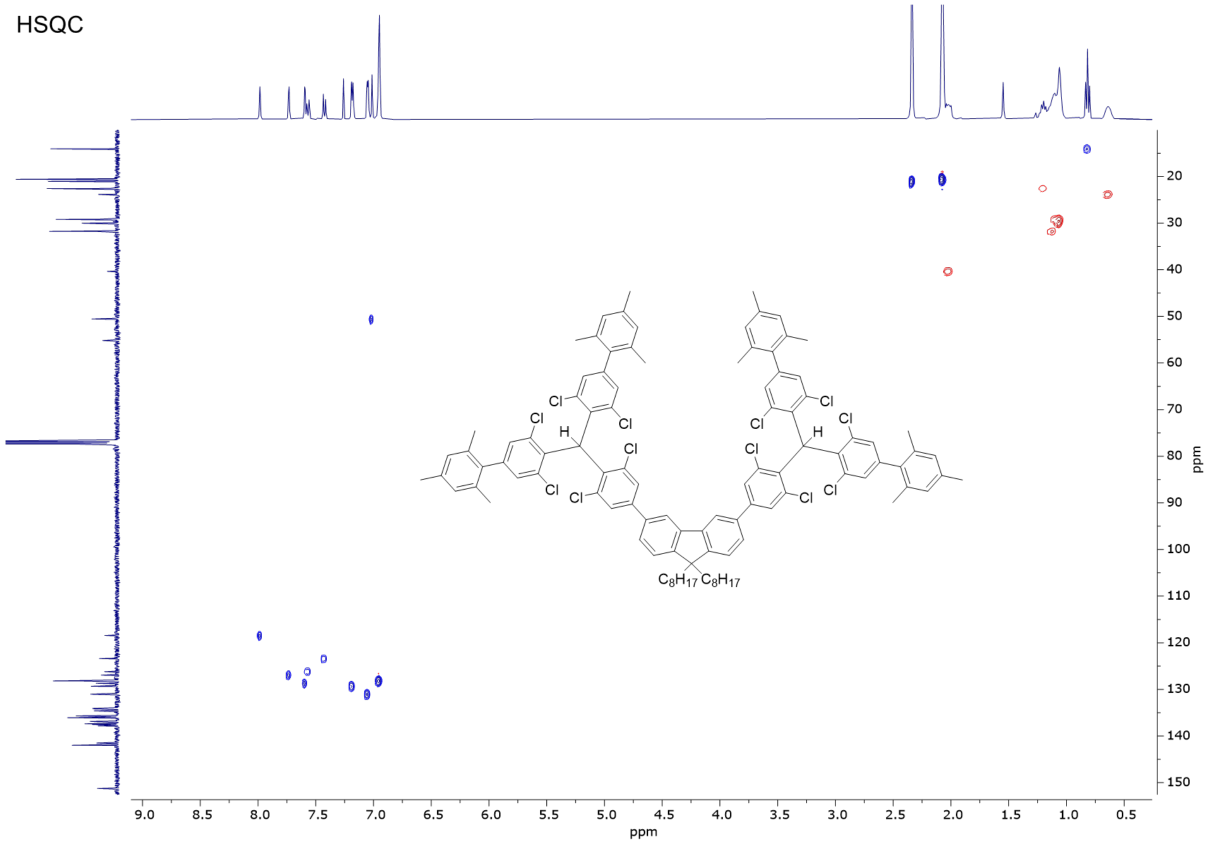

Cl
H
C_8H_{17} C_8H_{17}
ppm

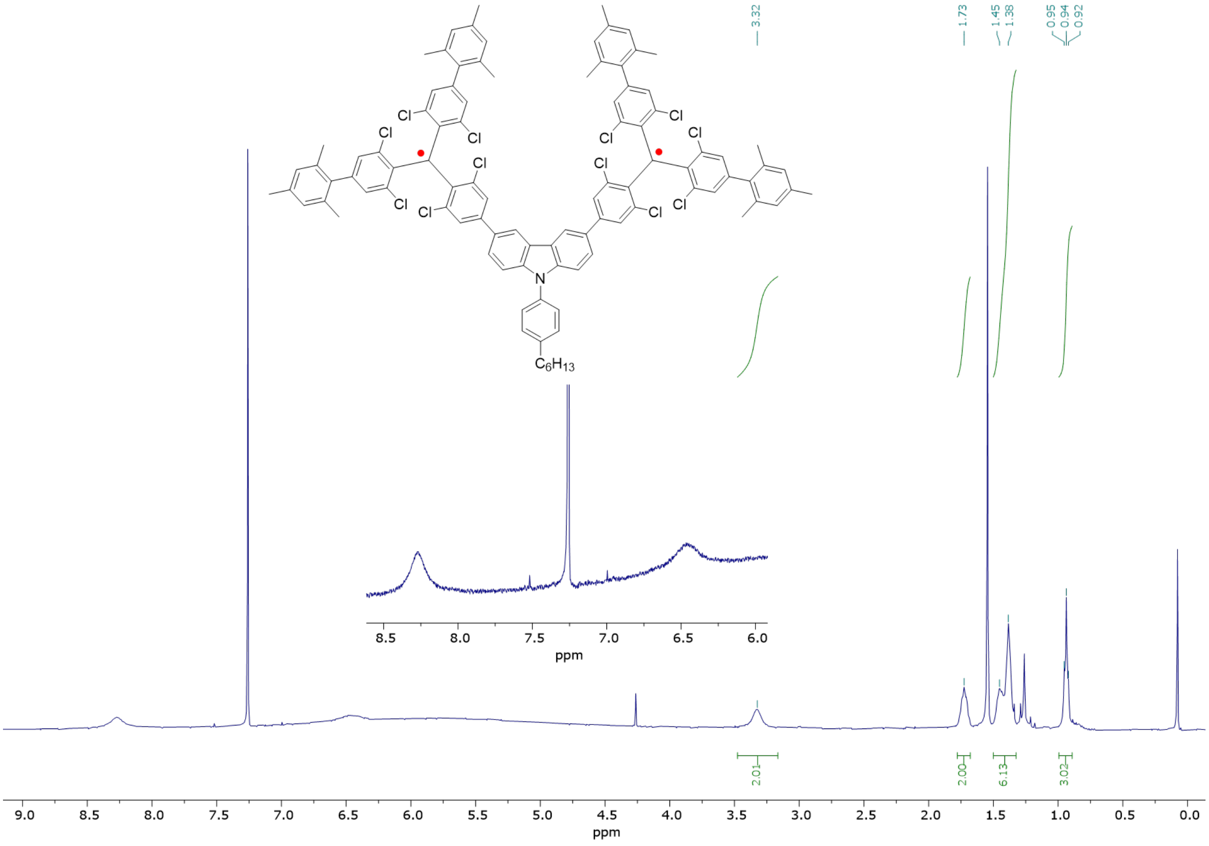

3.32
1.73
1.45
1.38
0.95
0.94
0.92
Cl
N
C_6H_{13}
2.01
2.00
6.13
3.02
ppm

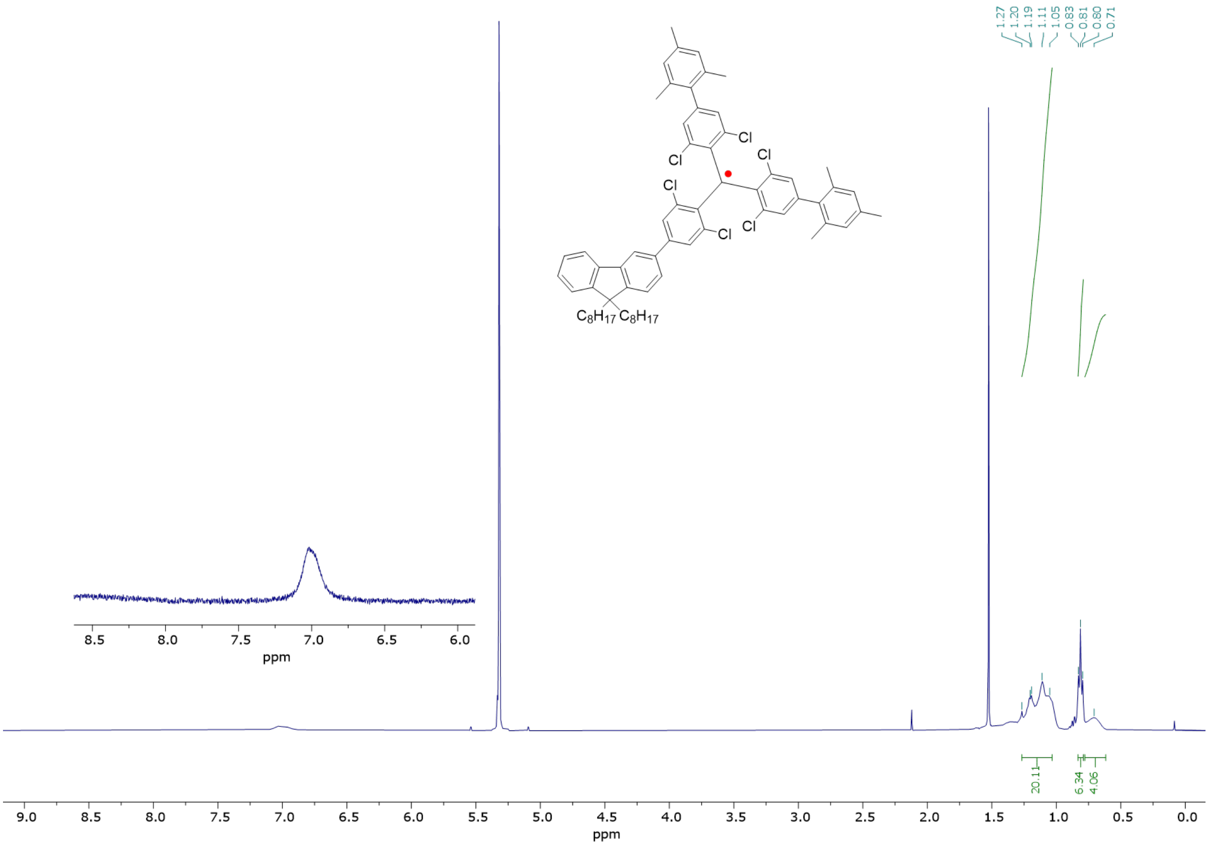
1.27
1.20
1.19
1.11
1.05
0.83
0.81
0.80
0.71
Cl
Cl
Cl
Cl
Cl
Cl
C8H17 C8H17
20.11
6.34
4.06
ppm
ppm

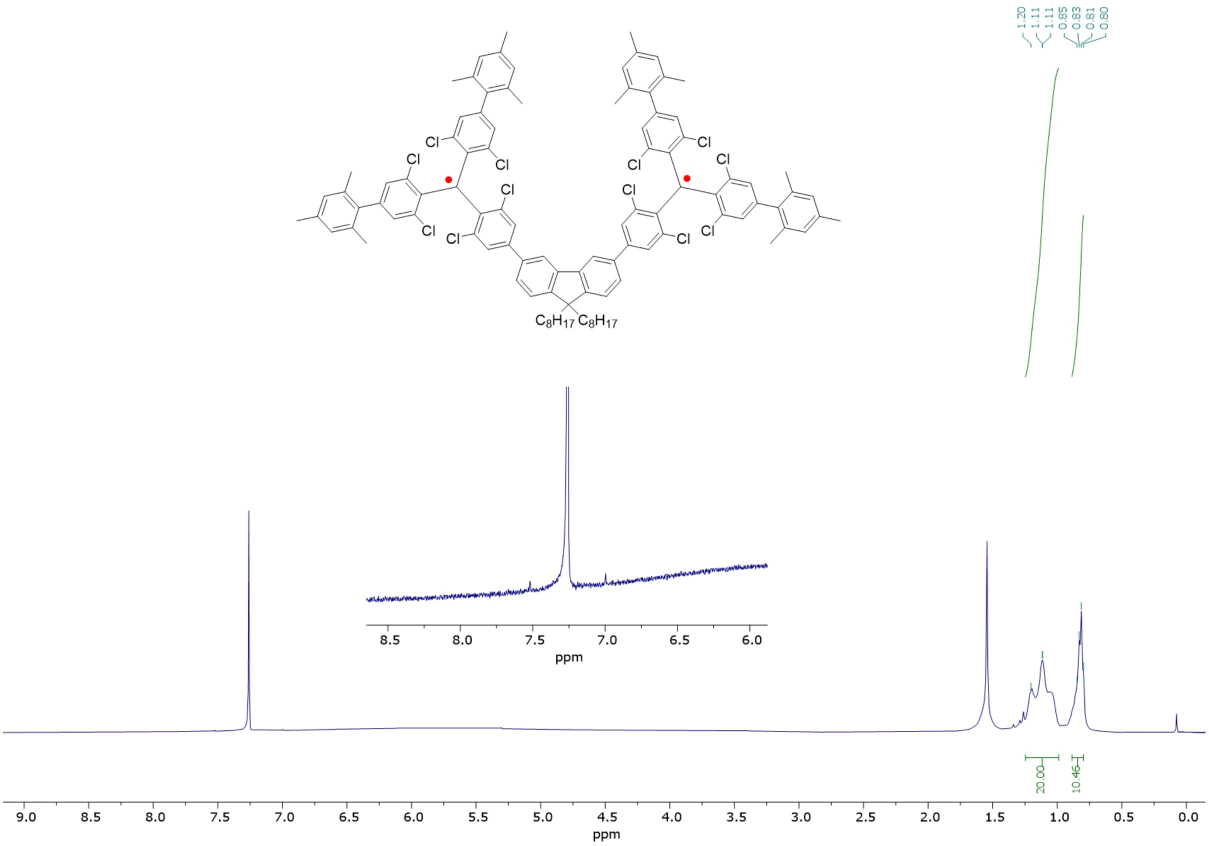
1.20
1.11
1.11
0.85
0.83
0.81
0.80
Cl
Cl
Cl
Cl
Cl
Cl
Cl
Cl
Cl
Cl
Cl
Cl
C8H17 C8H17
20.00
10.46
ppm
ppm

II. Steady State Optical Properties

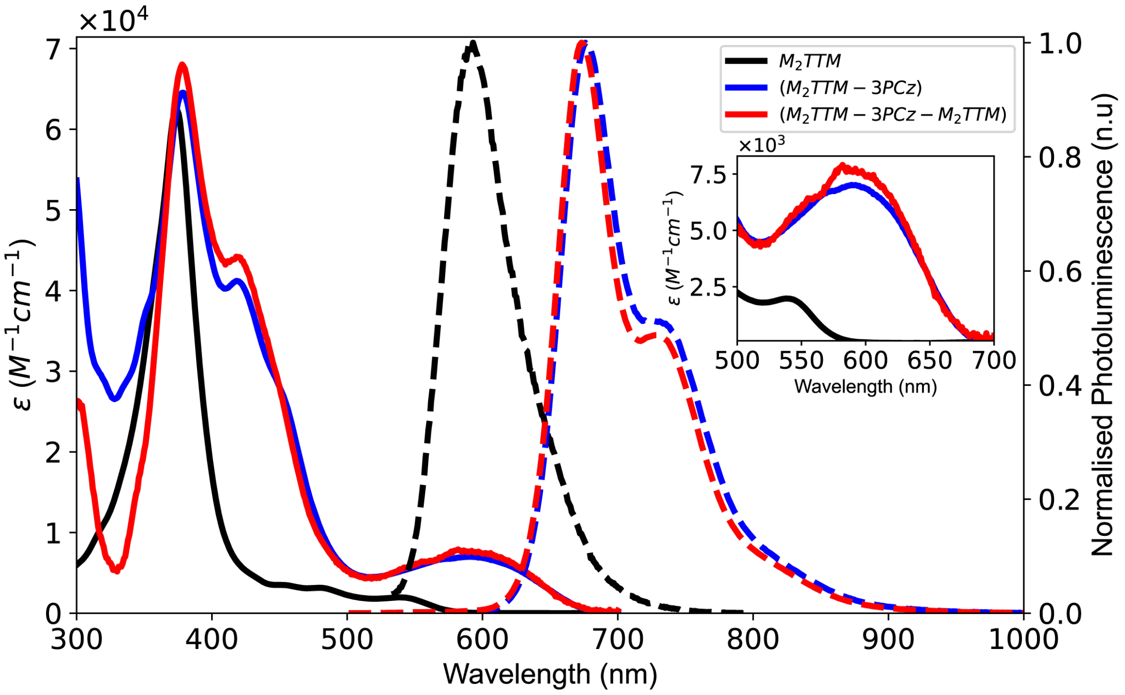

Figure 5: Steady state Photophysics of the -3-Phenylcarbazole(-3PCz) bridged system. *The absorption and photoluminescence spectra are presented for the unsubstituted monoradical M_2TTM (in black), the 3PCz-substituted monoradical M_2TTM-3PCz (in blue), and the 3PCz-substituted diradical M_2TTM-3PCz-M_2TTM (in red). These spectra were acquired under ambient conditions following a 532 nm excitation in a 100 μM toluene solution. The 3PCz-substituted monoradical and diradical exhibits a 90 nm (0.280 eV) red-shift compared to the unsubstituted radical M_2TTM.*

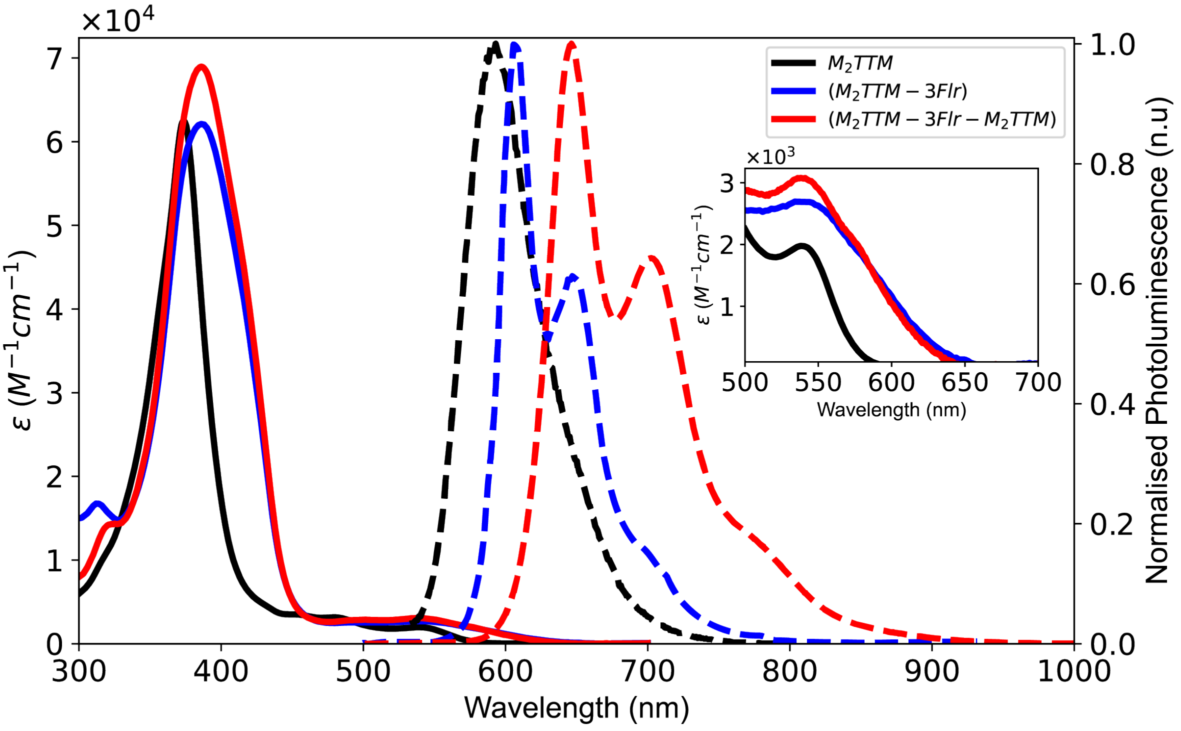

Figure 6: Steady state Photophysics of the -3-fluorene(-3Flr) bridged system. *The absorption and photoluminescence spectra are presented for the unsubstituted monoradical M_2TTM (in black), the 3Flr-substituted monoradical M_2TTM-3Flr (in blue), and the 3Flr-substituted diradical M_2TTM-3Flr-M_2TTM (in red). These spectra were acquired under ambient conditions following a 532 nm excitation in a 100 μM toluene solution. The M_2TTM-3Flr-M_2TTM diradical exhibits a 30 nm (0.1 eV) red-shift compared to the monoradical M_2TTM-3Flr and a 50 nm (0.2 eV) red-shift compared to the unsubstituted radical M_2TTM.*

Molecule	$\lambda_{abs,onset}$ (nm)	λ_{PL} (nm)	Φ_{PL} (%)	τ_{PL}(ns)	k_{rad} (ns^{-1})	k_{nonrad} (ns^{-1})
**M_2TTM**	580.0	595.0	12	21	0.0057	0.0419
**M_2TTM-3PCz**	670.0	680.0	94	8	0.1160	0.0074
**M_2TTM-3PCz-M_2TTM**	670.0	680.0	96	12	0.0774	0.0032
**M_2TTM-3Flr**	620.0	605.0	9	9	0.0101	0.120
**M_2TTM-3Flr-M_2TTM**	620.0	635.0	92	10(52%), 106(48%)	0.0939(T) 0.0087(S)	0.0082(T) 0.0007(S)

Table II.1: Steady state optical properties of all monoradicals and diradicals. *For the Triplet(T) and Singlet(S) type emission we observed that integrated emission counts do not change, implying that the PLQE from the low temperature state has not changed.*

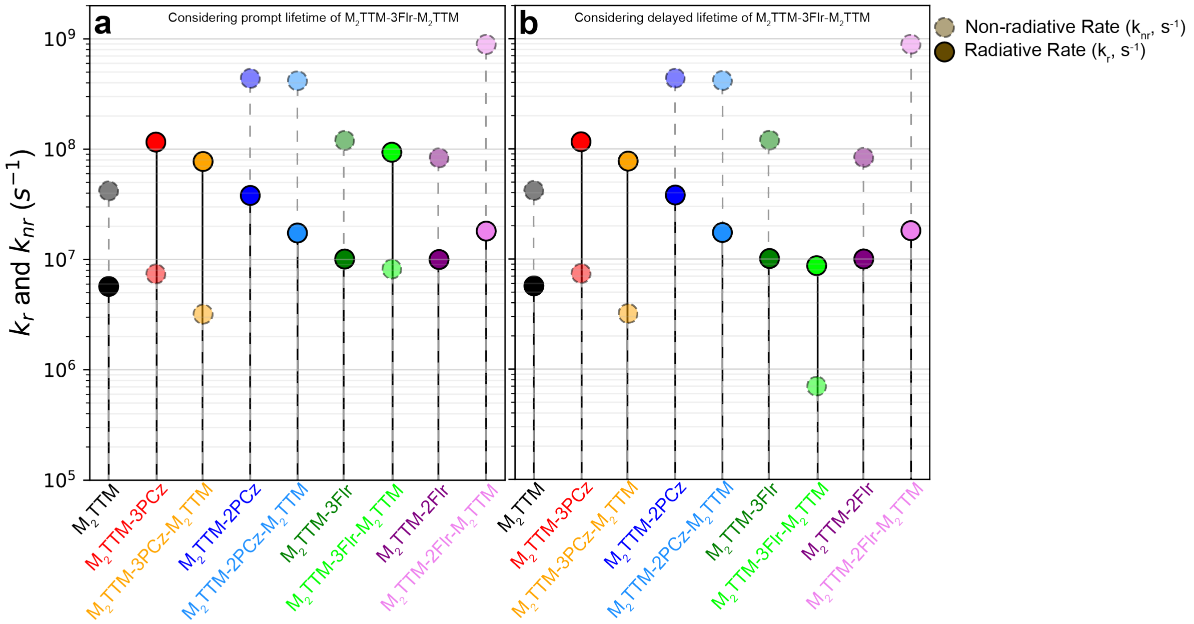

Figure 7: Radiative and Non-radiative lifetimes of all investigated systems. (a) *Considering the prompt lifetime of the M_2TTM-3Flr-M_2TTM diradical.* ***(b)*** *Considering the prompt lifetime of the M_2TTM-3Flr-M_2TTM diradical. We observe that by using a carbazole linker the k_r decreases when going from monoradical to diradical in both 3,6- and 2,7-linkages. Interestingly the k_{nr} value decreases by a large value when going mono- to diradical with a 3,6 linkage on Carbazole which allows us to maintain high luminescence efficiency in the diradical. If you consider the 2,7-linkage the radiative rate drops much more than the nonradiative rate when going from mono to diradical which causes an over-all decrease in the luminescence efficiency. In the 3,6-Fluorene bridged system we find that the radiative rate increases by 10x while the non-radiative rate decreases by 10x when going from monoradical to the diradical, this disparity in radiative and nonradiative rates is the underlying cause for a massive increase in quantum efficiency that is observed in the diradical. If we consider the delayed lifetime, the radiative rate is the same as the monoradical but the non-radiative rate drops by 100x in the diradical, yet again leading to a large PLQE.*

Reference	Molecule	λ$_{PL}$ (nm)	PLQE (%)	Alternant	MPL (%)	ODMR (%)
10.1021/jacs.3c01076 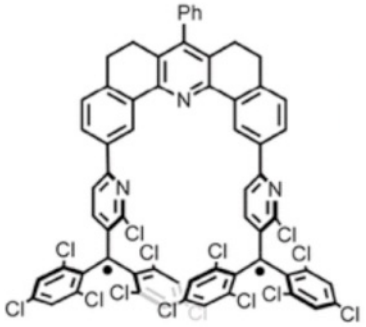	TTM-THDBA-TTM	650<br>720	1.2%<br>2.7%	no	+10% (650nm) -30% (720nm) At 14.5T and 4.2K	n.a
10.1021/jacs.4c11116 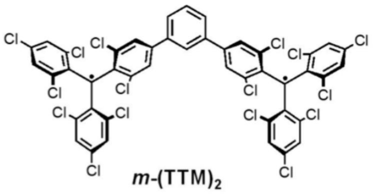	TTM-*m*Ph-TTM	620<br>680	0.6%	yes	n.a	0.05% (100mT, 2.8GHz, 85K)
10.1021/jacs.4c03972 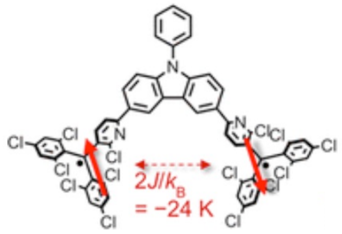	PyTM-3PCz-PyTM	670	28%	no	+300% (670nm) At 18T and 4.2K	n.a
Our Work 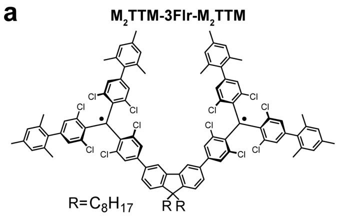	**M$_2$TTM-3FIr-M$_2$TTM**	**630<br>700**	**>90%**	**yes**	**+100% (630nm) -100% (700nm) At 4T, at 0.25K-20K**	**-10% (630nm) +10% (700nm) At 4.2T, 120GHz, 0.25K-20K**

Table 2 Comparison of recent trityl diradical materials.

We wish to highlight what we consider the important differences between our work and previously reported diradicals. We attribute the improved magneto-optical properties to a combination of the correct distance between radical centres, enforced by the fluorene bridge, which does not possess a donating Nitrogen lone pair such that it breaks the alternant symmetry and introduces charge-transfer character. Additionally, the bulkier mesityl group maintain enables greater sample purity (through suppressed side reactions during the synthesis) and maintains the restricts the conformational freedom between the two radical centers.

III. Time Resolved Emission Spectroscopy

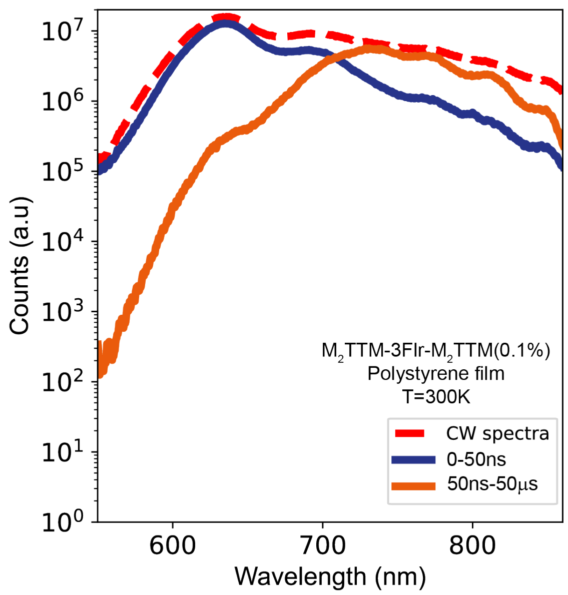

Figure 8: Component analysis of time resolved photoluminescence. *This shows the PL measured in the [0-50) ns time interval and in the (50 ns-50 μs] time interval measured with the iCCD detector, alongside the CW PL spectrum. This shows that there is about equal contributions from the short and long time spectra.*

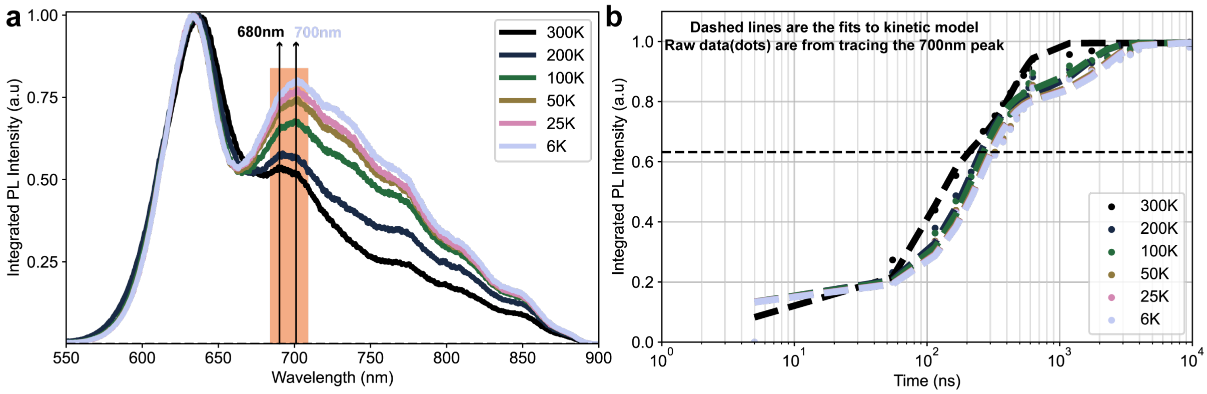

Figure 9: Kinetic modelling of TRPL at different temperatures. (a) *Continuous wave PL Spectra of he spin-coated 0.1wt% M_2TTM-3Flr-M_2TTM doped in Polystyrene, while cooling down from 300K to 6K. Cooling down is accompanied by an increase in the relative contribution of the 690nm PL peak relative to the 635nm emission. A further narrowing of the 635nm emission peak is also observed.* ***(b)*** *Kinetics extracted from the 680nm-700nm region(dots) shaded in (a) is modelled using a biexponential decay model(dashed lines) at each temperature to extract the prompt and delayed kinetics parameters. A lengthening of the kinetics for prompt and delayed decays are observed upon cooling. Spectra are obtained after exciting a the spin coated film with a 532nm excitation beam at a 6μJcm^{-2} fluence. Parameters for this fitting are provided in Table 2 below.*

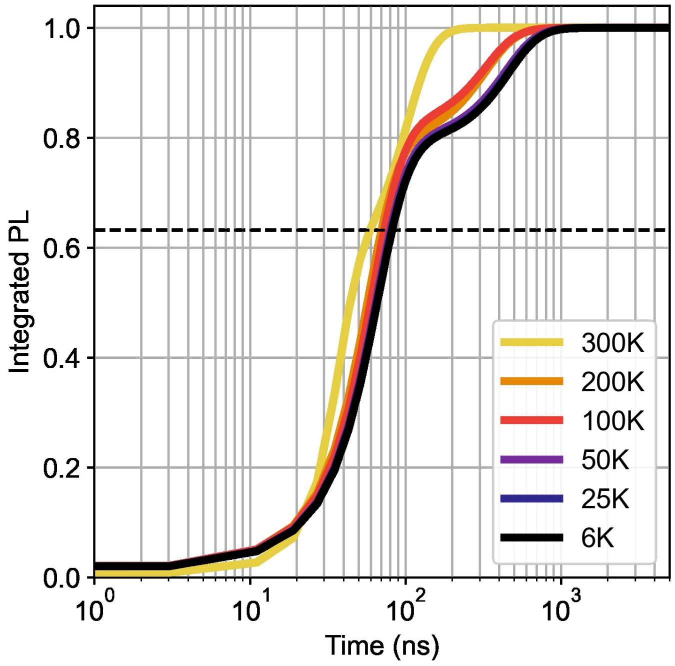

Figure 10: Integrated Photoluminescence kinetics at different temperatures. *The integrated kinetics taking into account the full emission spectrum is shown at different temperatures for the spin coated film of 0.1wt% M_2TTM-3FIr-M_2TTM doped in Polystyrene. Spectra are obtained after exciting a the spin coated film with a 532nm excitation beam at a 6μJcm^{-2} fluence.*

Temperature(K)	k_1 (ns^{-1})	k_2 (ns^{-1})	A_1 (a.u)	A_2(a.u)	$t_{0,1}$ (ns)	$t_{0,2}$ (ns)
300	0.096	0.0059	0.53	0.47	9.49	37.82
200	0.088	0.0037	0.55	0.45	16.42	123.50
100	0.085	0.0025	0.55	0.45	17.83	128.00
50	0.086	0.0019	0.56	0.44	18.95	171.6
25	0.085	0.0019	0.55	0.45	19.17	177.0
6	0.083	0.0018	0.56	0.44	19.19	179.5

Table 3: Fitting parameters used to model the integrated PL kinetics traces of the integrated PL at different temperatures. *The function* $f(t) = \frac{A_1}{1+e^{-k_1(t-t_{0,1})}} + \frac{A_2}{1+e^{-k_2(t-t_{0,2})}}$ *is used to fit to the integrate PL curve.*

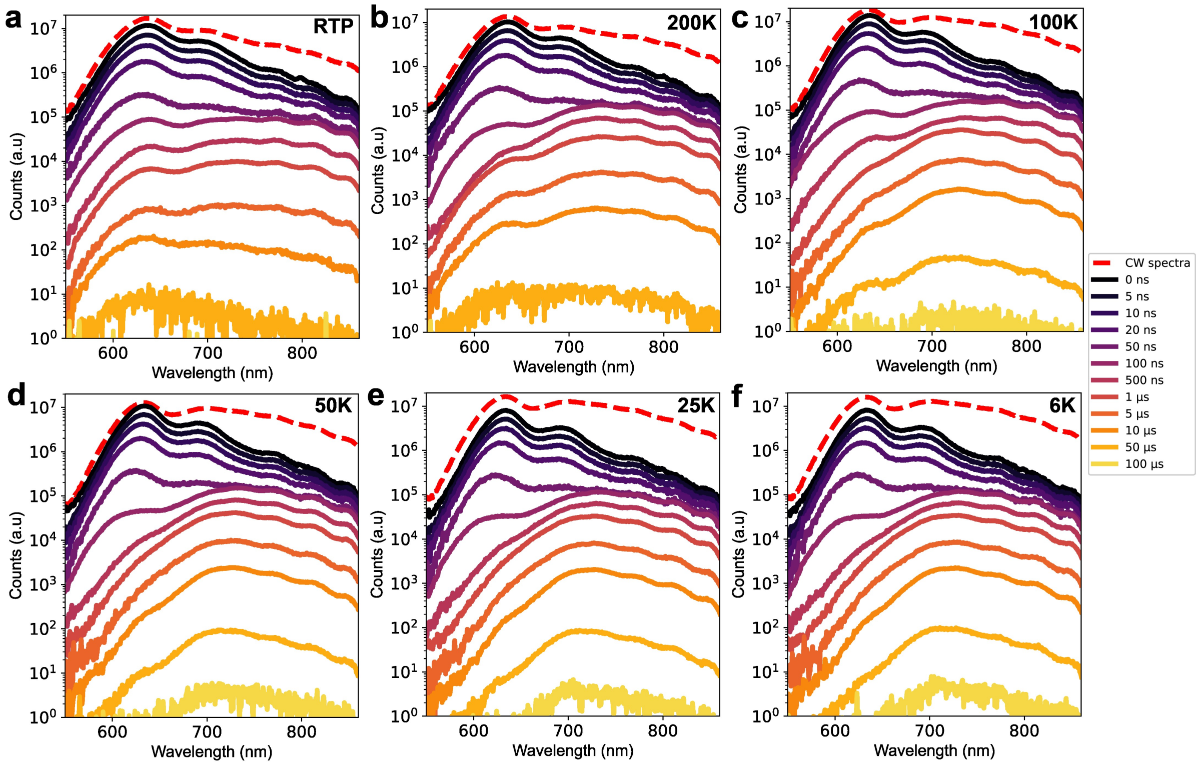

Figure 11: Spectral Slices during time resolved photoluminescence recorded at different temperatures. *Spectral slices at 0ns, 5ns, 10ns, 20ns, 50ns, 100ns, 500ns, 1μs, 5μs, 10μs, 50μs, 100μs are presented for temperatures of **(a)** 300K, **(b)** 200K, **(c)** 100K, **(d)** 50K, **(e)** 25K, **(f)** 6K. The continuous wave spectra(red, dashed lines) are provided at each temperature for comparison to the temporally evolving spectra. In all cases we have reached the noise-floor by 100μs, beyond which we cannot detect any PL spectra. Spectra are obtained after exciting a spin coated 0.1wt% M_2TTM-3FIr-M_2TTM doped Polystyrene film with a 532nm excitation beam and 6μJcm^{-2} fluence.*

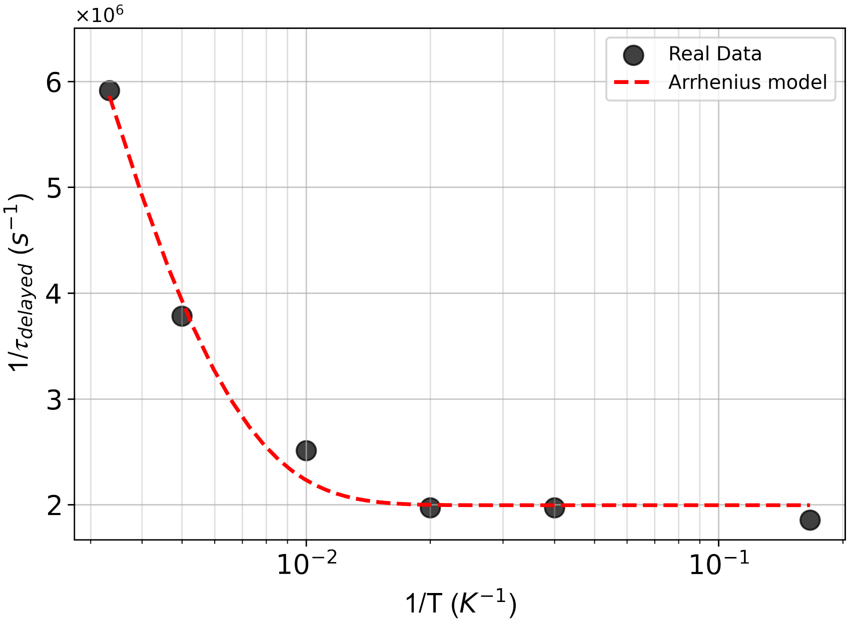

Figure 12: Arrhenius equation fitting. *By fitting the Arrhenius model to the observed change in delayed emission we can quantify an activation barrier of 10.15 meV (~118 K) to the population of the*

red emitting state that exhibits delayed emission. This was performed by fitting the exponential-containing equation, $k = Ae^{-E_{act}/kT}$, to a plot of rate constant (k) against inverse-temperature ($\frac{1}{T}$).

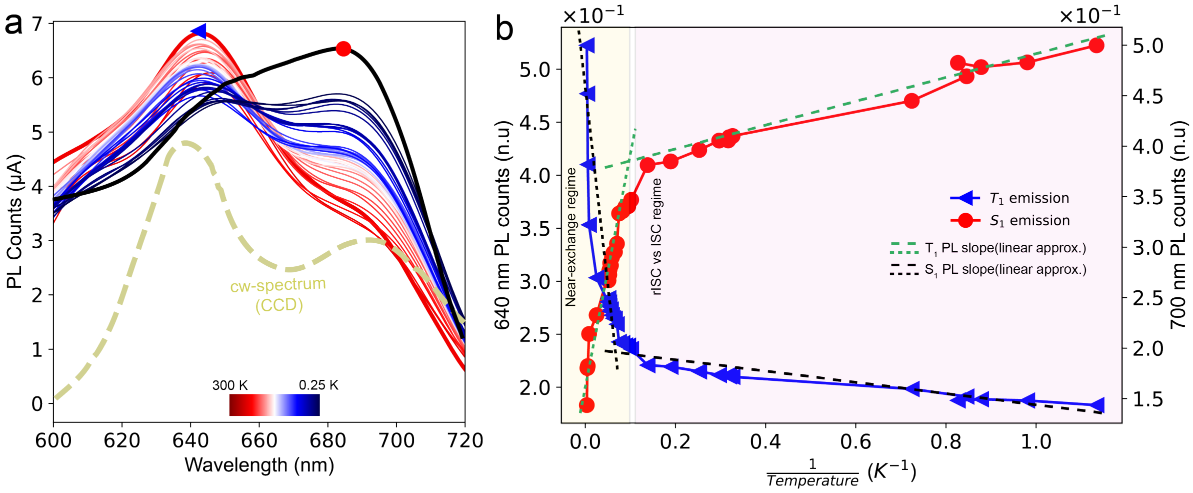

Figure 13: Spectral evolution of 0.1wt% M2TTM-3FIr-M2TTM doped Polystyrene films when cooling down. ***(a)*** *Spectrum recorded at regular temperature intervals from 300 K to 0.25 K. The cw-spectrum recorded using a CCD camera has been shown in yellow for reference. The closed-blue triangle and closed-red circle denote the points tracked for the temperature dependent trend. PL, in all cases is measured by exciting a M_2TTM-3FIr-M_2TTM(0.1%):Polystyrene spin-coated film using a 532 nm laser.* ***(b)*** *The curie-like inverse-temperature dependent intensity at 640 nm(blue, closed triangles) and 690-700 nm (red, closed circles). The different slopes are denoted using dashed lines. We are able to see that the temperature dependence has 2 regimes: (i) The 300-40 K regime where rISC and ISC compete with each other. ISC will dominate over rISC at temperatures below activation energy (118 K). (ii) The near-exchange regime. We obtain an exchange energy of 1.2 K, so below the rISC regime temperature the main process thermally susceptible process is the stabilization of the singlet level below the triplet level by approaching exchange. It is important to note that the integrated emission counts do not change much upon cooling indicating that the red emitting ~700nm state is almost as efficient as the blue emitting 640nm state. Both states are equally emissive.*

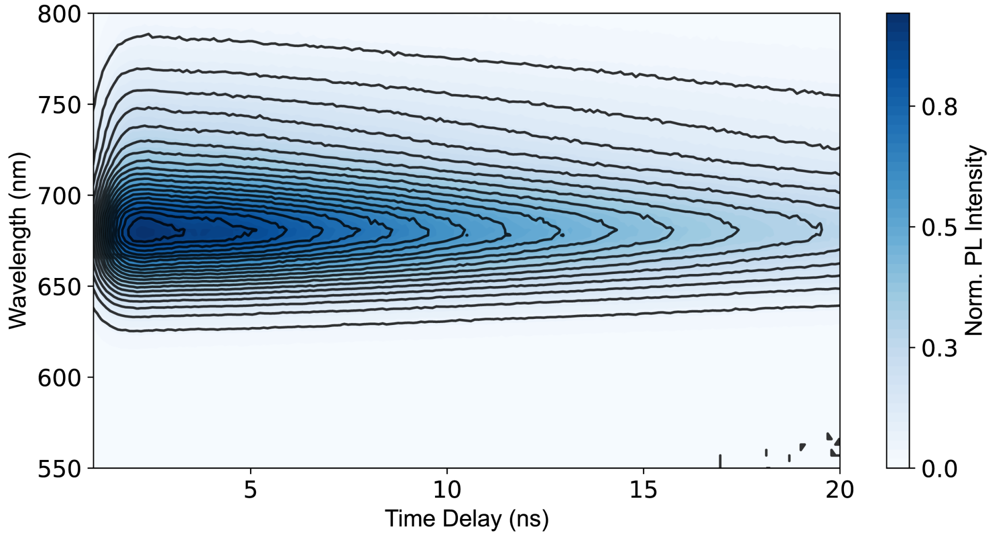

Figure 14: Time resolved emission spectra of M2TTM-3PCz-M2TTM. *A 532nm excitation of fluence 10μJcm-2 was used on a 0.1wt% film of the emitter in polystyrene.*

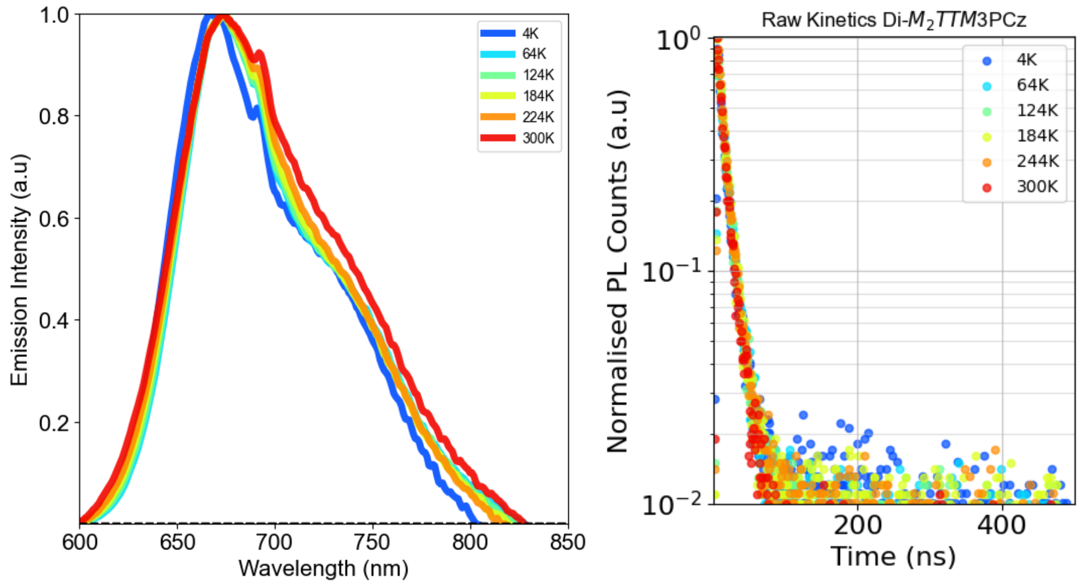

Figure 15: Temperature dependent emission spectra of M2TTM-3PCz-M2TTM: (Left) *Normalised emission intensity measured at each temperature.* ***(Right)*** *TCSPC traces of the emission at each temperature. Note the absence of any meaningful changes when changing the temperature. In all cases we use a 532 nm pulsed excitation with a fluence of 6μJcm^{-2}. There is no spectral change, indicating that the singlet state here is dark, which we cover in later Supporting Information section IV.C.*

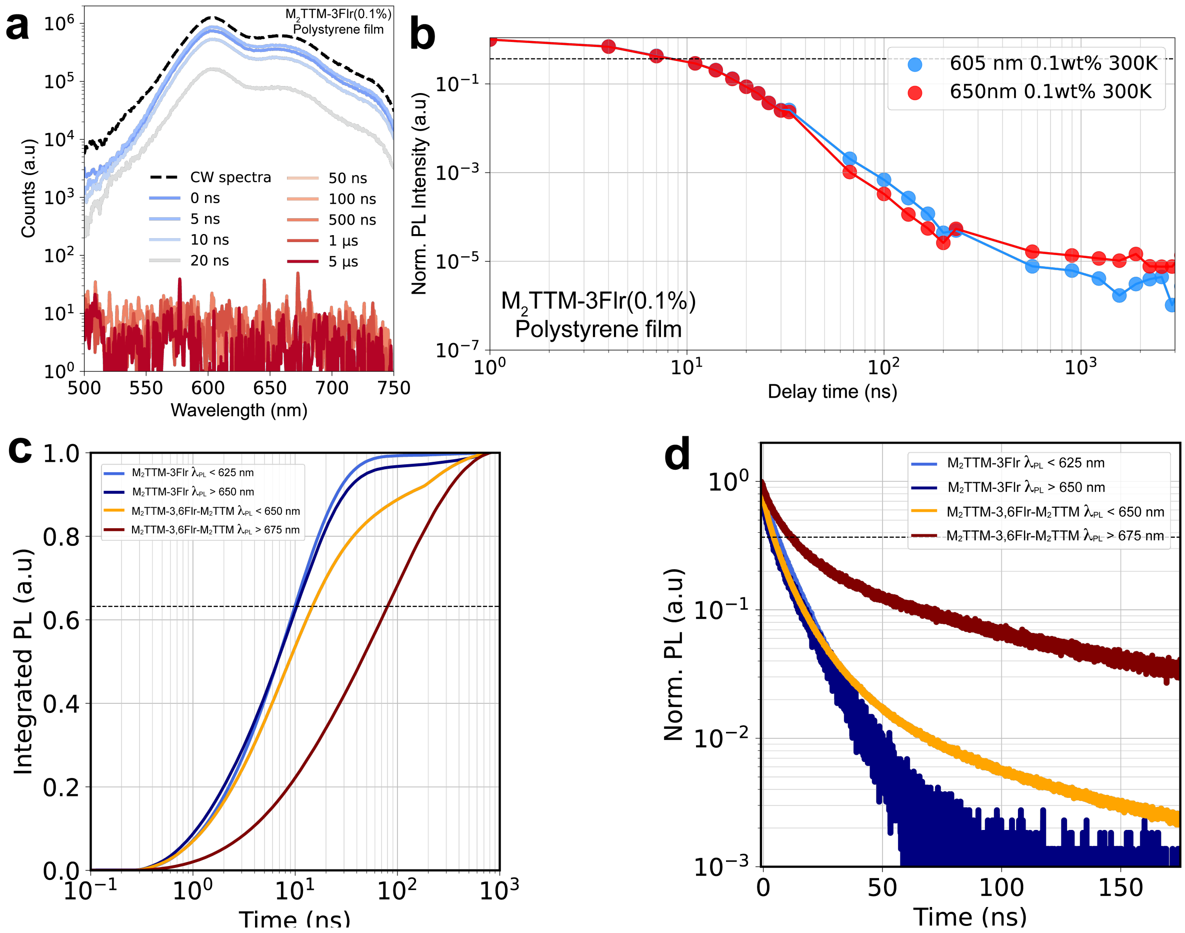

Figure 16: Transient PL studies on monoradical M2TTM-3FIr. (a) *Spectral slices cut at quoted time points compared to the steady state CW spectrum (black dashed lines).* ***(b)*** *The decay kinetic traces recorded by tracking the normalised intensity at 605 nm and 650 nm, the calculated lifetime is*

4.7 ns, the 1/e line is denoted by the black-dashed line in the subfigure. ***(c)*** *Integrated PL kinetic traces for the different peaks in the monoradical and diradical.* ***(d)*** *PL decay kinetic traces of the monoradical and diradical. (a)-(b) are measured using an electronically gated iCCD camera, (c)-(d) are measured using time-correlated single photon counting (TCSPC). In all cases we measure 0.1wt% doped polystyrene thin-film, where a pulsed pump at 532 nm with a fluence of 6μJcm^{-2} is used to excite the sample.*

IV. Magneto-Optical Spectroscopy

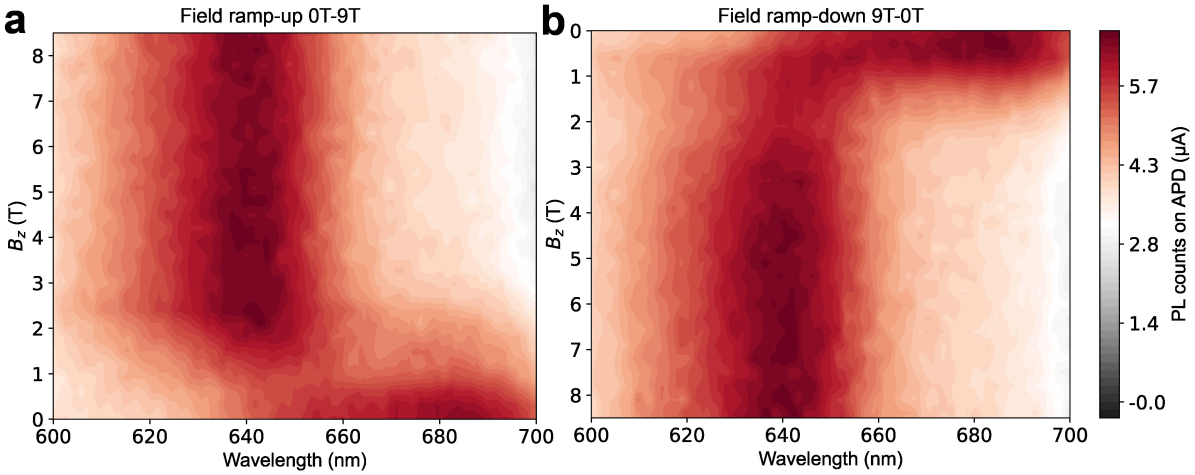

***Figure 17: Magneto-photoluminescence heatmaps at 250mK. (a)** Spectral evolution while ramping up field from 0T to 9T. **(b)** Spectral evolution while ramping down field from 9T to 0T. PL was recorded for a 0.1wt% M_2TTM-3Flr-M_2TTM doped polystyrene film excited using a 405nm laser excitation with 90nW of power to ensure proper thermalisation of the sample stage.*

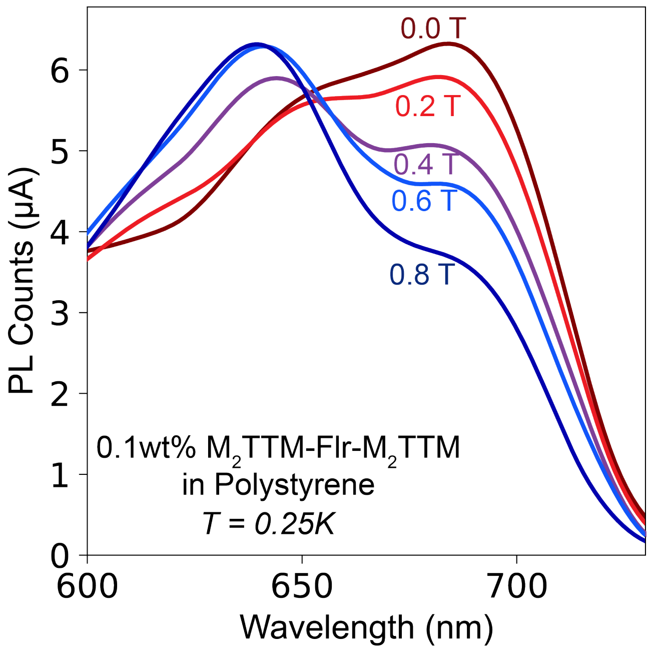

***Figure 18: Magneto-photoluminescence spectra at 250mK in 0.2T intervals shown till the exchange interactions is over-powered.** We observe the smooth transition from singlet to triplet with the ratio of 700 nm : 640 nm PL changing, tacking the population change as spins are shifted is shifted from spin-Singlet to spin-Triplet in the ground state of the molecule.*

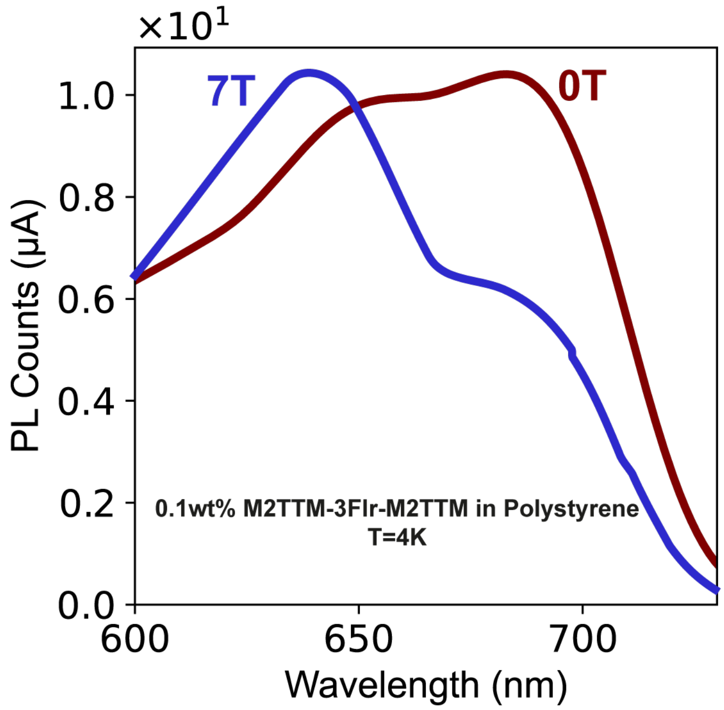

Figure 19: Magneto-photoluminescence at 4K. *Spectral evolution while ramping up field from 0T to 7T. PL was recorded for a 0.1wt% M_2TTM-3FIr-M_2TTM doped polystyrene film excited using a 405nm laser excitation with 150nW of power to ensure proper thermalisation of the sample stage.*

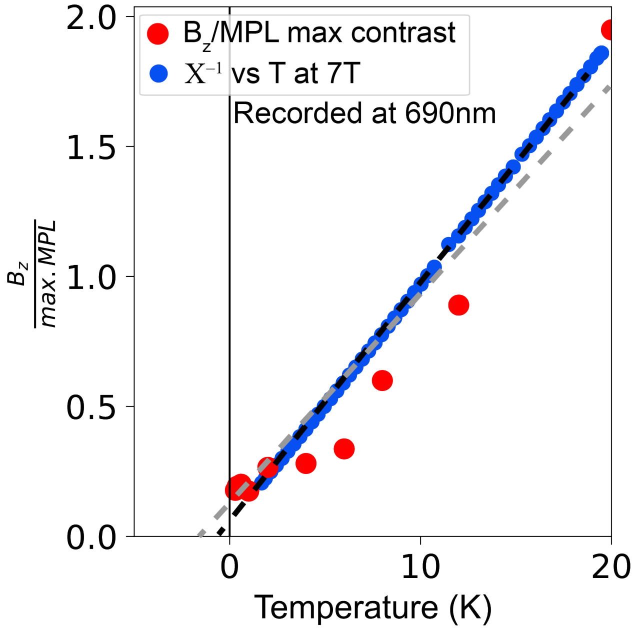

Figure 20: Comparing the susceptibility to the inverse of magPL. *We compare the inverse-magnetization temperature dependence to the inverse of the maximum MPL contrast to show that both show the same type of Curie-Weiss law obeying behaviour that is expected from antiferromagnetic systems.*

IV.A Fitting the Magnetic field response of the PL

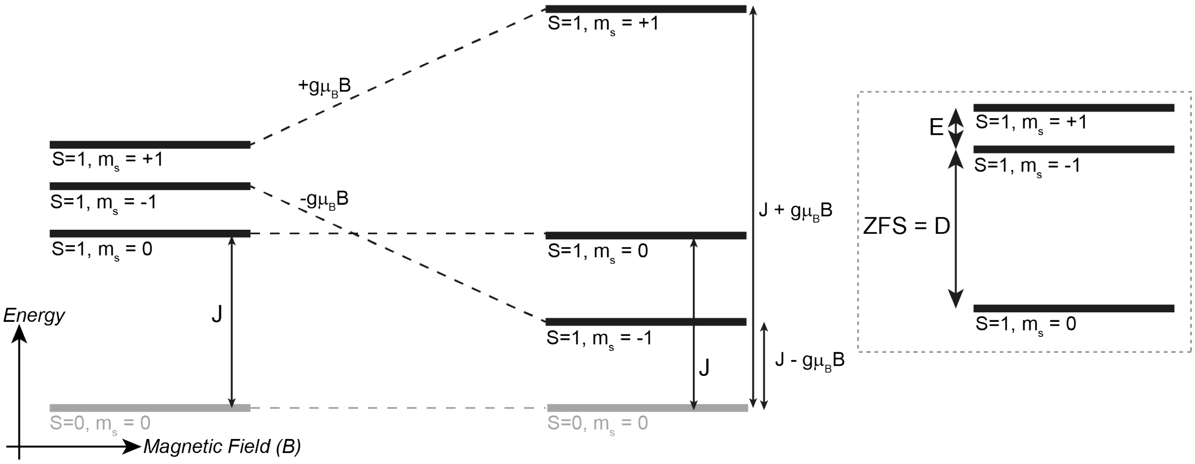

Figure 21. The magnetic field dependent level splitting for the ground state. *We use an antiferromagnetic level structure where the singlet (S=0) is lower in energy than the triplet (S=1). The inset to the right shows the zero-field splitting of the triplet ground state. The zero-field splitting (ZFS or D) is the energy difference between the m_s = -1 and m_s = 0 sublevels of the triplet. The anisotropy (E) parameter is the energy difference between the m_s = -1 and m_s = +1 sublevels of the triplet. The zeeman interaction terms for the m_s = -1, +1 levels is $+g\mu_B B$ and $-g\mu_B B$.*

We use *E({S,m_s}, B)* as our terminology where S is the net spin state and ms is the sublevel spin quantum number, B is the applied magnetic field magnitude. Based on the energy level diagram in Figure 21 we can define the magnetic field dependent energy of the ground state as:

E({0,0}, B) = 0.0, (I.1)

E({1,0}, B) = J, (I.2)

E({1,-1}, B) = J - $g\mu_B B$, (I.3)

E({1,+1}, B) = J + $g\mu_B B$, (I.4)

Using I.1-4 we can construct the population of the singlet {0,0} level from the boltzmann distribution as follows:

$P_S(B, J, T) = \frac{1}{1+e^{{-J}/{T}}+e^{{-J+B}/{T}}+e^{{-J-B}/{T}}}$, (eq.1)

In eq.1 we have neglected the dimensionless constants $g\mu_B$ and k_B to be equal to 1, which is true in the reduced units basis. The broadening over the magnetic field arises from an uncertainity in the distribution of exchange (J) values which we can factor in as a Gaussian distribution of J values with a mean value J_0 and standard deviation ΔJ:

$$\frac{1}{\sqrt{2\pi}\Delta J}\int_{-\infty}^{\infty} P_S(B,J,T)\, e^{{-(J-J_0)^2}/{2\Delta J^2}} dJ, \quad \text{(eq. 2)}$$

The effective smearing from the convolution of a series of Fermi-functions can be defined as an effective temperature from the units of this solution, which is in Kelvin. This effective temperature T_{eff} is found to be:

$T_{eff} = \sqrt{T^2 + (\Delta J)^2}$, (eq. 3)

We can numerically solve this problem for all the temperatures and magnetic-field dependencies:

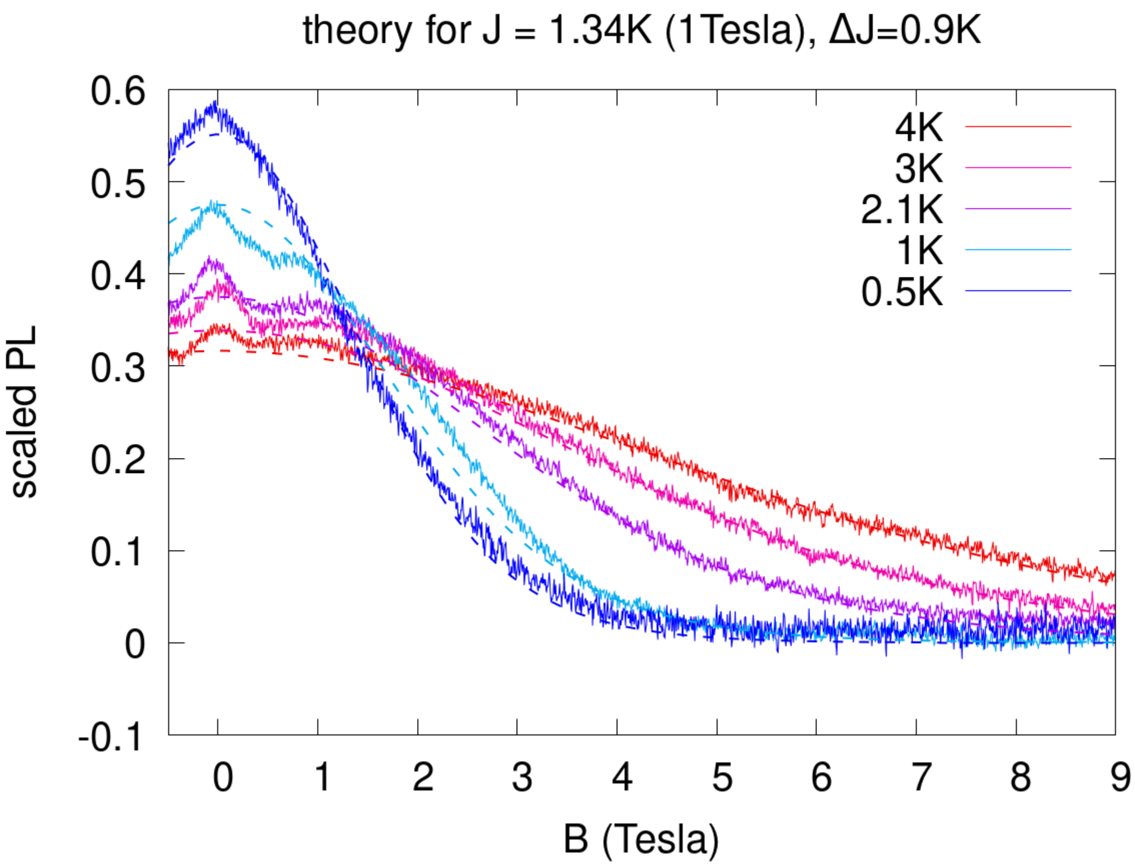

Figure 22: Solutions to (eq.2) fitted to the MPL behaviour at different temperatures showing excellent agreement.

From the fitted solutions we find that the standard deviation in exchange is 0.9 K indicating a set of triplet states perhaps in the ground state where we have a triplet with an anntiferromagnetic exchange energy 1.34 K and another triplet with antiferromagnetic exchange energy of 0.24 K.

IV.1 A phenomenological 4-state model:

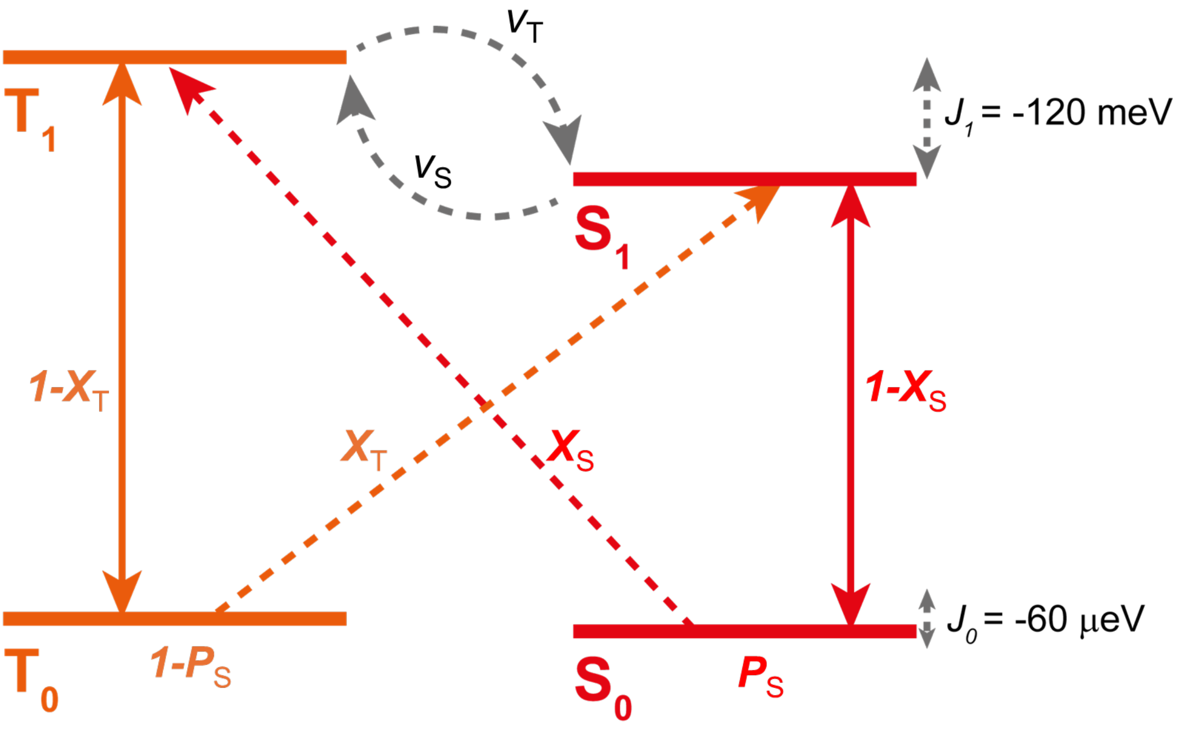

Figure 23: Transfer probability based model for magneto-photoluminescence. *We assume a 4-level system. The ground state is composed of spin-singlet and spin-triplet states, labeled S_0 and T_0 respectively with an antiferromagnetic exchange interaction of -60 µeV. The excited state also has the corresponding singlet and triplet excited states, labeled S_1 and T_1 respectively. The population of the singlet state, the ground state, is labeled as P_S. Assuming that the ground state only contains S_0 and T_0 we can say that the ground state triplet population is $1\text{-}P_S$. The rate of intersystem crossing from T_1 to S_1 is v_T and the rate of the reverse intersystem crossing from S_1 to T_1 is v_S. The probability of cross-excitations is labeled as X_S for the $S_0 \rightarrow T_1$ excitation and X_T for the $T_0 \rightarrow S_1$ excitation. Assuming no intermediate excitations, the vertical excitation probabilities are $1\text{-}X_S$ for the $S_0 \rightarrow S_1$ excitation and $1\text{-}X_T$ for the $T_0 \rightarrow T_1$ excitation.*

We consider the 4-level electronic structure as described in Figure 23. At the lowest temperatures the following limiting scenarios are possible:

- T ~ 0 K, B = 0 T: The S_0 population is $P_S = 1$, thus the T_0 population is $1\text{-}P_S = 0$. (I.1)
- T ~ 0 K, B = 9T: The S_0 population is $P_S = 0$, thus the T_0 population is $1\text{-}P_S = 1$. (I.2)

We assume that since the emission intensity is the same for the $S_1 \rightarrow S_0$ and $T_1 \rightarrow T_0$ and the PLQE at room temperature is ~1.0 we assume that all generated excitons radiatively decay down, ignoring the nonradiative coupling for now, which is expected to be small. So we can safely apply the approximation: PL(S_1) = Population of S_1 and PL(T_1) = Population of T_1 where PL referes to photoluminescence.

With these basic limiting equations and assumptions in place we can define the PL de-excitation probability from S_1 and T_1 as:

$PL(S_1) = A_S[(\mathbf{1\text{-}}\boldsymbol{X}_S)\boldsymbol{P}_S+\boldsymbol{X}_T(\mathbf{1\text{-}}\boldsymbol{P}_S)]$,   (eq. 1)

$PL(T_1) = A_T[(\mathbf{1\text{-}}\boldsymbol{X}_T)(\mathbf{1\text{-}}\boldsymbol{P}_S)+\boldsymbol{X}_S\boldsymbol{P}_S]$,   (eq. 2)

Where A_S and A_T are arbitrary proportionality constants for the singlet and triplet.

First we turn to the derivation of the expected MPL response we derive from the Singlet emission at ~700 nm, which we abbreviate as MPL(S_1):

PL(S_1, 0 T) = A_S[**1-*X*$_S$**] , using I.1 (eq. 3)

PL(S_1, 9 T) = A_S[***X*$_T$**] , using I.2 (eq. 4)

So, MPL(S_1)= $\frac{PL(S_1, 9\ T)}{PL(S_1, 0\ T)} = \frac{\boldsymbol{X}_T}{1\text{-}\boldsymbol{X}_S}$, (eq. 5)

Next we derive the expected MPL response we derive from the Triplet emission at 640 nm, which we abbreviate as MPL(T_1):

PL(T_1, 0 T) = A_T[$\boldsymbol{X}_S \boldsymbol{P}_S$] , using I.1 (eq. 6)

PL(T_1, 9 T) = A_S[**1-*X*$_T$**] , using I.2 (eq. 7)

So, MPL(T_1)= $\frac{PL(T_1, 9\ T)}{PL(T_1, 0\ T)} = \frac{1\text{-}\boldsymbol{X}_T}{\boldsymbol{X}_S}$, (eq. 8)

We can use the experimental MPL observation at 250 mK (Main, Figure 2.b) which gives us the values: MPL(S_1) = 0.5 (-50%) and MPL(T_1) = 1.15 (+15%). Equating these values to eq.5 and eq.8 we construct a system of linear equations with variables $\boldsymbol{X}_S$ and $\boldsymbol{X}_T$. Solving this linear system we obtain

$\boldsymbol{X}_S$ = 0.77 (result 1a) and $\boldsymbol{X}_T$ = 0.12 (result 1b)

To test the validity of this electornic structure we can first try to test if these cross-excitation probabilities can reproduce the temperature dependent intensity of singlet emission. We can compare the emission ~0 K and at 300 K:

PL(S_1, 0 T, 0 K) = A_S[(**1-*X*$_S$**)$\boldsymbol{P}_S$] = A_S x 0.23, (eq. 9.a)

PL(S_1, 0 T, 300 K) = A_S[P(↑↑) $\boldsymbol{X}_T$ + P(↑↓)(**1-*X*$_S$**)] = A_S x 0.147 , (eq. 9.b)

$\left.\frac{PL(S_1, 0\ T, 0\ K)}{PL(S_1, 0\ T, 300\ K)}\right|_{predicted}$ = 1.56, (result 2)

$\left.\frac{PL(S_1, 0\ T, 0\ K)}{PL(S_1, 0\ T, 300\ K)}\right|_{observed}$ = 1.6 (From Figure 13, 690-710 nm PL)

Where P(↑↑) and P(↑↓) are the triplet and singlet populations at ambient conditions which is mandated to be 0.75 and 0.25 by spin-statistics. We immediately see that the prediction has a deviation of 2% with the experiment which is an excellent agreement.

To further test the validity of the theory we can we can try to test if the cross-excitation probabilities can reproduce the temporal evolution of singlets at room temperature which we define as the population at ~300 fs to that at 5 ns once intersystem crossing has finished and equillibrium is established between triplets and singlets in the excited state:

P_{TA}(S_1, 300 K, *early-time*) = (**1-*X*$_S$**) P(↑↓) = 0.0575, (eq. 10.a)

P_{TA}(S_1, 300 K, *late-time*) = P(↑↓) (**1-*X*$_S$**) + P(↑↑) $\boldsymbol{X}_T$ = 0.1475, (eq. 10.b)

$\left.\frac{P_{TA}(S_1, 300\ K,\ \textit{late-time})}{P_{TA}(S_1, 300\ K,\ \textit{early-time})}\right|_{predicted}$ = 2.57, (result 3)

$\left.\frac{P_{TA}(S_1, 300\ K,\ \textit{late-time})}{P_{TA}(S_1, 300\ K,\ \textit{early-time})}\right|_{observed}$ = 2.51 (From value of 0.98 eV PIA in Main Figure 2.d)

Thus our prediction has a 2.8% deviation from the experimental observation. It is worth noting that in TA we can also have a further 1% uncertainity due to probe-noise. But nevertheless the agreement of the prediction from cross-excitation probabilities calculated from magPL data with the experimental temperature and transient behaviour shows us the validity of this general 4-level structure and the correctness of the $\boldsymbol{X}_S$ and $\boldsymbol{X}_T$.

These probabilities are actually related to the rISC and ISC rates. If they were absent then we would have PL(T_1, 300 K):PL(S_1, 300 K) = 3.0, this is not true as the correct answer here is 1.6. Using this knowledge, we can then instead write PL(T_1, 300 K):PL(S_1, 300 K) = 0.856 A_T/ 0.144A_S = 1.6 → A_T = 0.27A_S.

The level-structure:

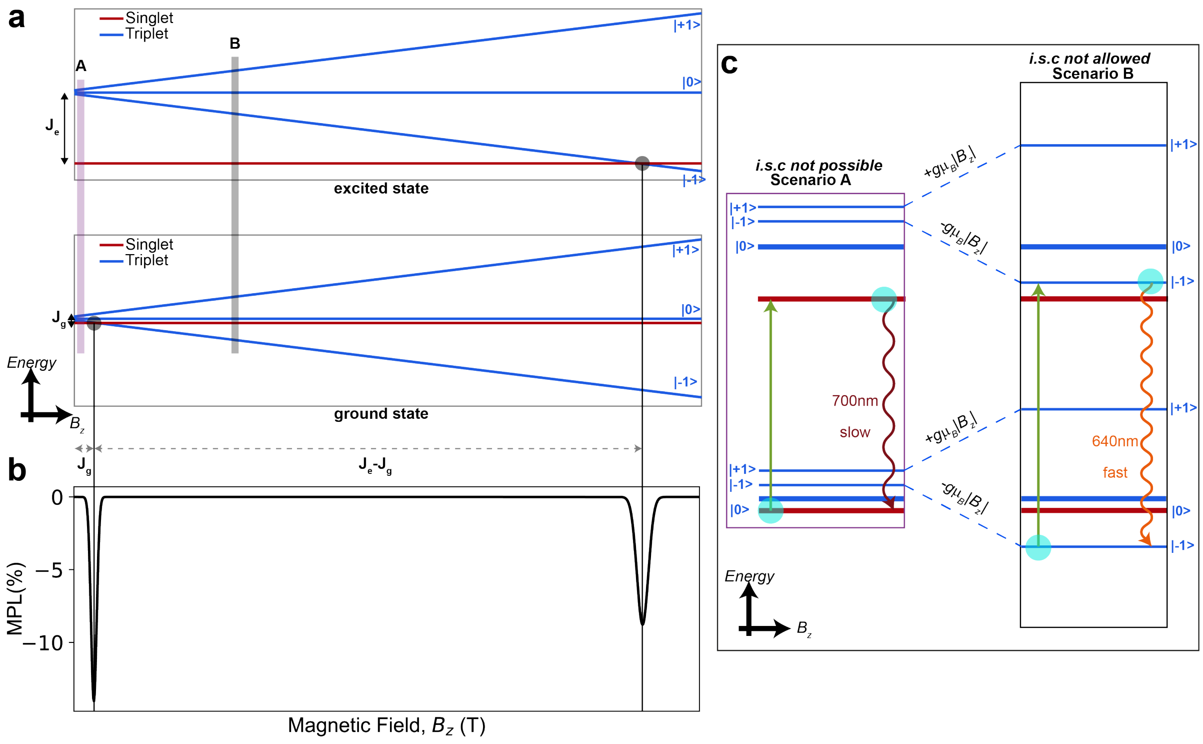

Figure 24: Model for magnetic field effect on photoluminescence. ***(a)*** *Ground state and excited state zeeman diagram assuming a singlet(red) and triplet(blue) in both states. This provides a phenomenological model to correlate with the observed magnetic field effect on photoluminescence.* J_g *and* J_e *are the ground state and excited state exchange energies.* ***(b)*** *Simulated Magneto-photoluminescence curves depicting where we hope to see resonance points. We observe resonance features in the emission at magnetic fields providing a zeeman energy comparable to* J_g *and* J_e*.* ***(c)*** *Considering the emission spectrum we obtain by increasing the field we can construct 2 scenarios: (Scenario A)* $B_z < J_g$ *and (Scenario B)* $B_z > J_g$*. In Scenario A, a sub-exchange magnetic field is applied. We know that the emission is at 700 nm is slow and from a zwitterionic singlet* S_1 *state. The system is antiferromagnetic and <1.2 K the* S_0 *level is the true ground state of the system. Direct vertical excitation can populate only* S_1 *and no reverse-intersystem crossing could occur as the temperatures are far below the activation energy (36 meV). Thus all the PL is at 700 nm. Now in Scenario B, with fields above the exchange interaction, the triplet* m_s *= -1 sublevel is the ground state and is populated. We reason that due to an unfavorable i.sc from this sublevel there is virtually no repopulation of the S1 level and hence no 700 nm PL, as a consequence all the PL comes from the 640 nm triplet-emission channel. This spin-selective i.s.c has been shown to occur due to selection rules associated with the process*(*7*, *8*)

IV.2 Magnetic Field dependent photoluminescence of M_2TTM-3PCz-M_2TTM diradical

We performed temperature and magnetic field dependent measurements on the M_2TTM-3PCz-M_2TTM diradical, the results seem to qualitatively match those by Mizuno *et al* (*9*) .

As shown in Figure 25-26, when cooling down observe no change in spectra, the intensity of the PL goes down by 5x when cooling down from 100K to 0.5K. This can be attributed to populating the ground state singlet state which is dark in the M_2TTM-3PCz-M_2TTM diradical.

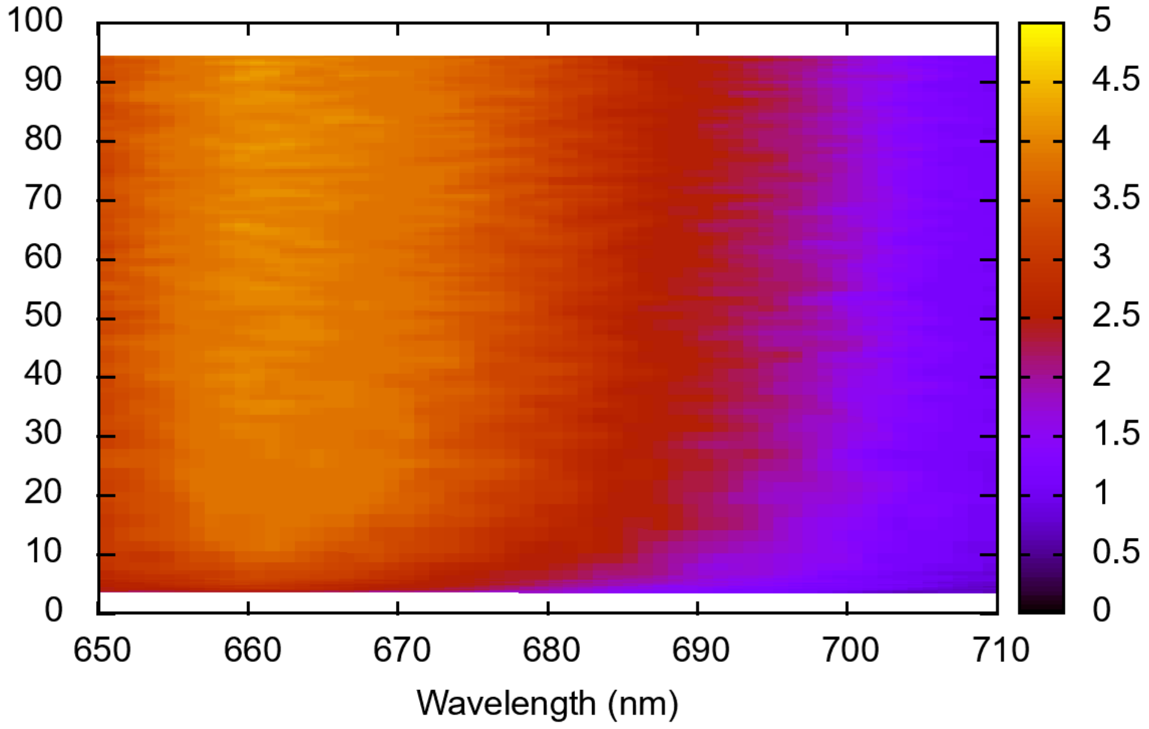

Figure 25: Temperature dependence of the photoluminescence of M2TTM-3PCz-M2TTM. *Spectra were recorded for a 0.1wt% M_2TTM-3PCz-M_2TTM doped polystyrene film excited using a 405nm laser excitation with 150nW of power to ensure proper thermalisation of the sample stage.*

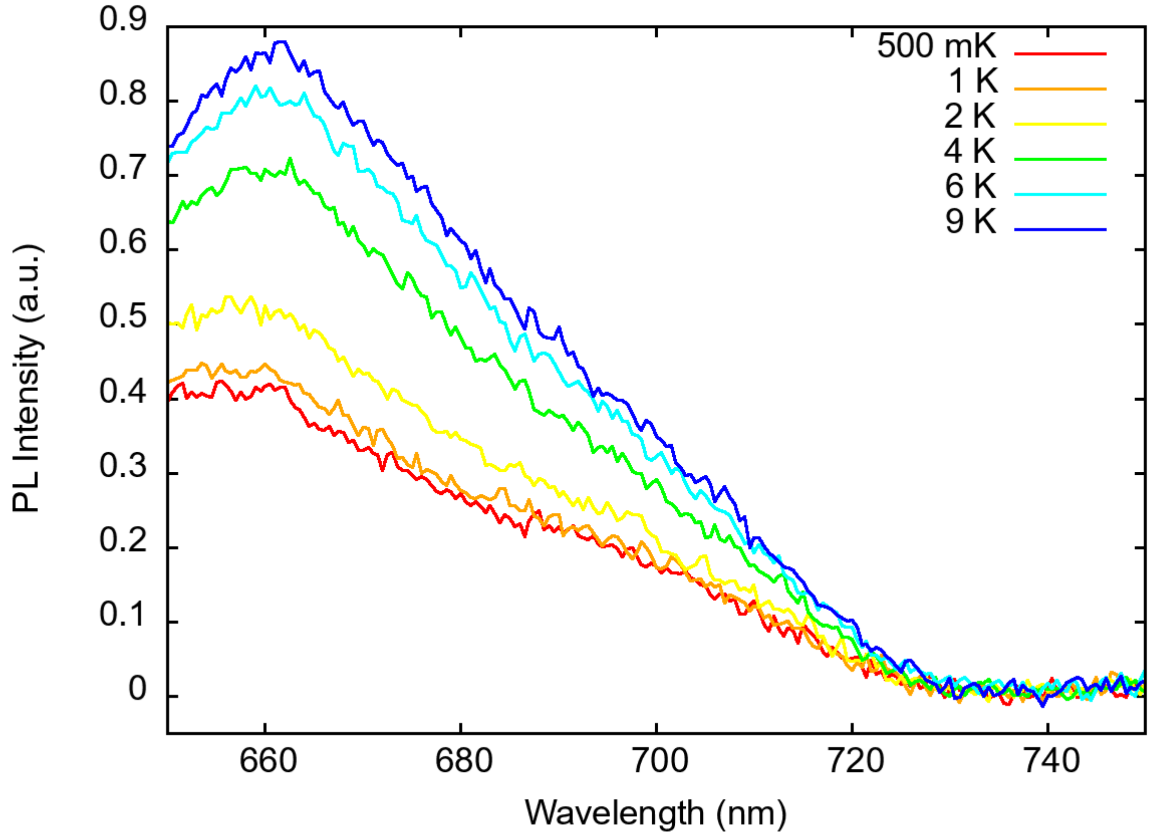

Figure 26: Spectral slices for the temperature dependence of the photoluminescence of M2TTM-3PCz-M2TTM. *Spectra were recorded for a 0.1wt% M_2TTM-3PCz-M_2TTM doped polystyrene film excited using a 405nm laser excitation with 150nW of power to ensure proper thermalisation of the sample stage.*

As shown in Figure 27-28, at 0.2K (where we are below exchange and have polarised the singlet ground state), the magnetic field increases the PL by 2x when ramped up from 0T to 9T. This can be attributed to populating the ground state triplet state which is bright, this is brought down below the singlet level by the Zeeman interaction. At these fields and temperatures the the m_s=-1 sublevel of the triplet is populated in the ground state.

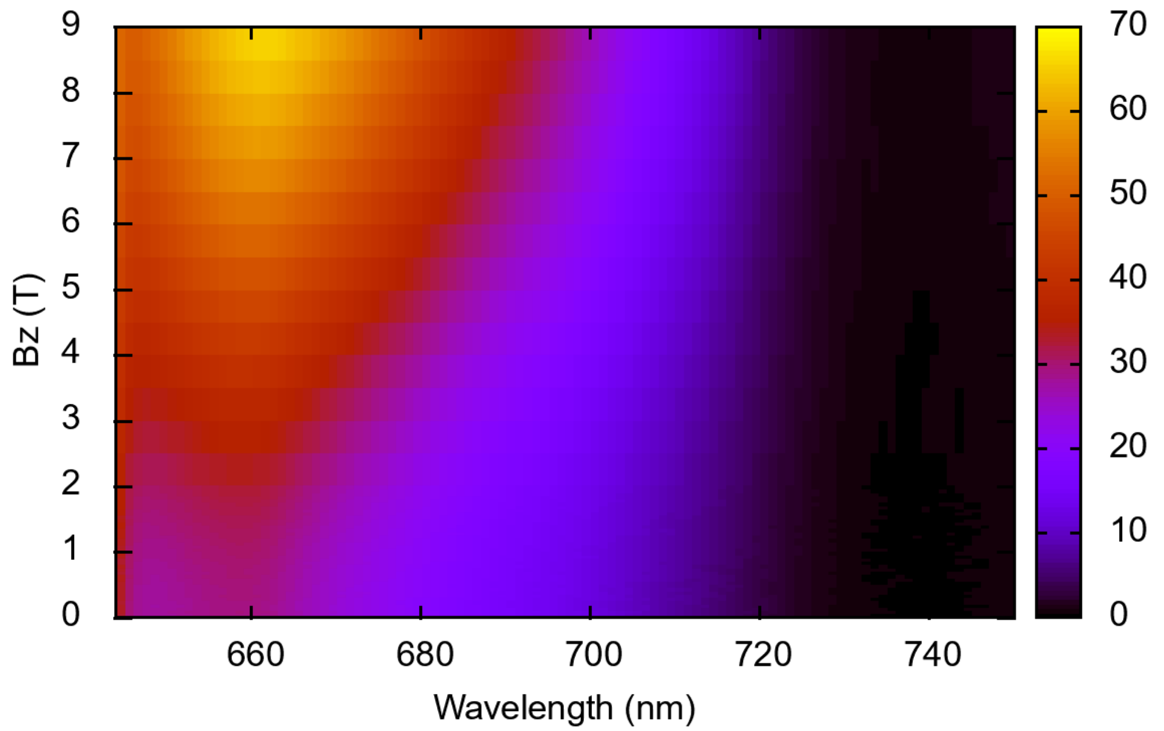

Figure 27: Magnetic field dependence of the photoluminescence of M2TTM-3PCz-M2TTM. *Spectra were recorded at 0.2 K for a 0.1wt% M_2TTM-3PCz-M_2TTM doped polystyrene film excited using a 405nm laser excitation with 150nW of power to ensure proper thermalisation of the sample stage.*

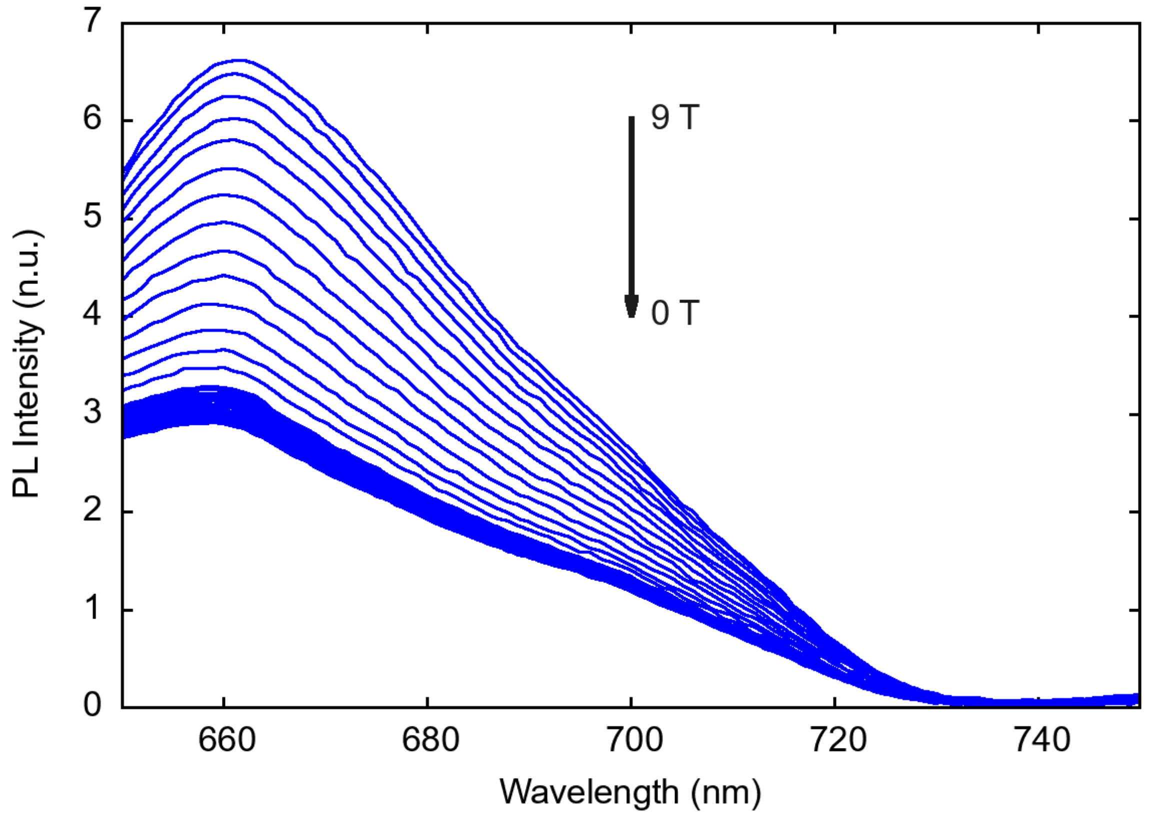

Figure 28: Spectral slices of the magnetic field dependence of the photoluminescence of M2TTM-3PCz-M2TTM. *Spectra were recorded at 0.2 K for a 0.1wt% M_2TTM-3PCz-M_2TTM doped polystyrene film excited using a 405nm laser excitation with 150nW of power to ensure proper thermalisation of the sample stage.*

As shown in Figure 29-30,the MPL behaviour is thermally dependent. This is because as temperature is increased above the antiferromagnetic exchange (around 3K here) the triplet and singlet levels are thermalised thus the relative population difference at temperatures above exchange with an applied field is lower than the relative population difference at sub-exchange temperatures. The triplet PL is still increased with a magnetic field due to the Zeeman interaction

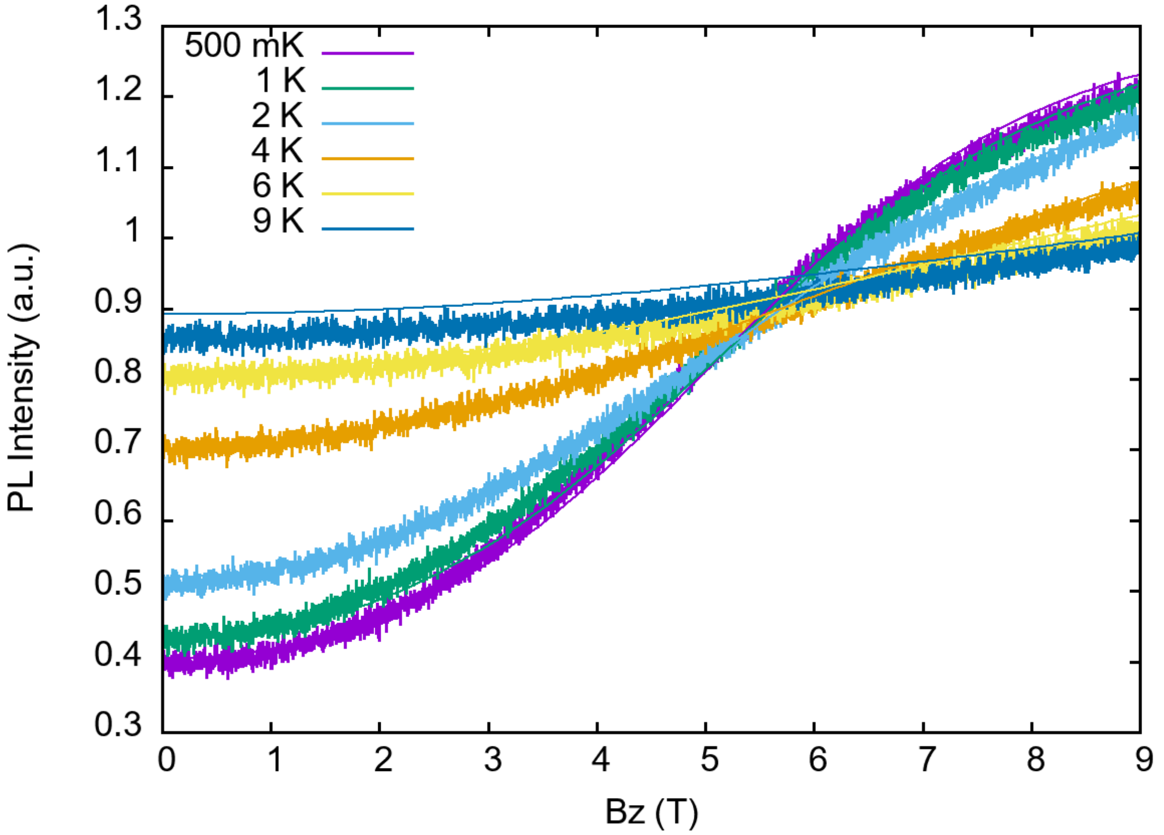

Figure 29: Temperature dependence of the MPL for M2TTM-3PCz-M2TTM. *Spectra were recorded from 0.5K to 9K for a 0.1wt% M_2TTM-3PCz-M_2TTM doped polystyrene film excited using a 405nm laser excitation with 150nW of power to ensure proper thermalisation of the sample stage.*

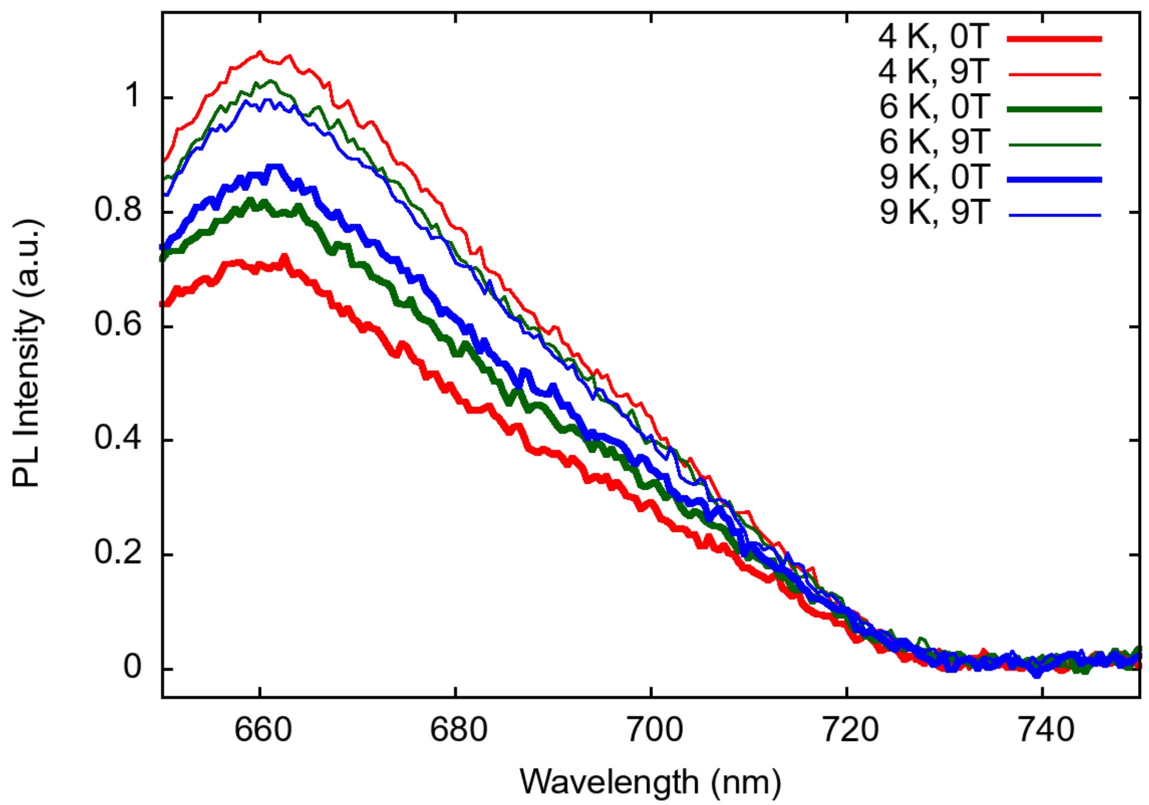

Figure 30: Spectral slices for the temperature dependence of the MPL for M2TTM-3PCz-M2TTM. *MPL Spectra at 0T and 9T were recorded at 4K, 6K and 9K for a 0.1wt% M_2TTM-3PCz-M_2TTM doped polystyrene film excited using a 405nm laser excitation with 150nW of power to ensure proper thermalisation of the sample stage.*

As shown in Figure 31, the MPL behaviour is independent of laser fluence indicating this is a single-molecule behaviour and does not involve bimolecular artefacts.

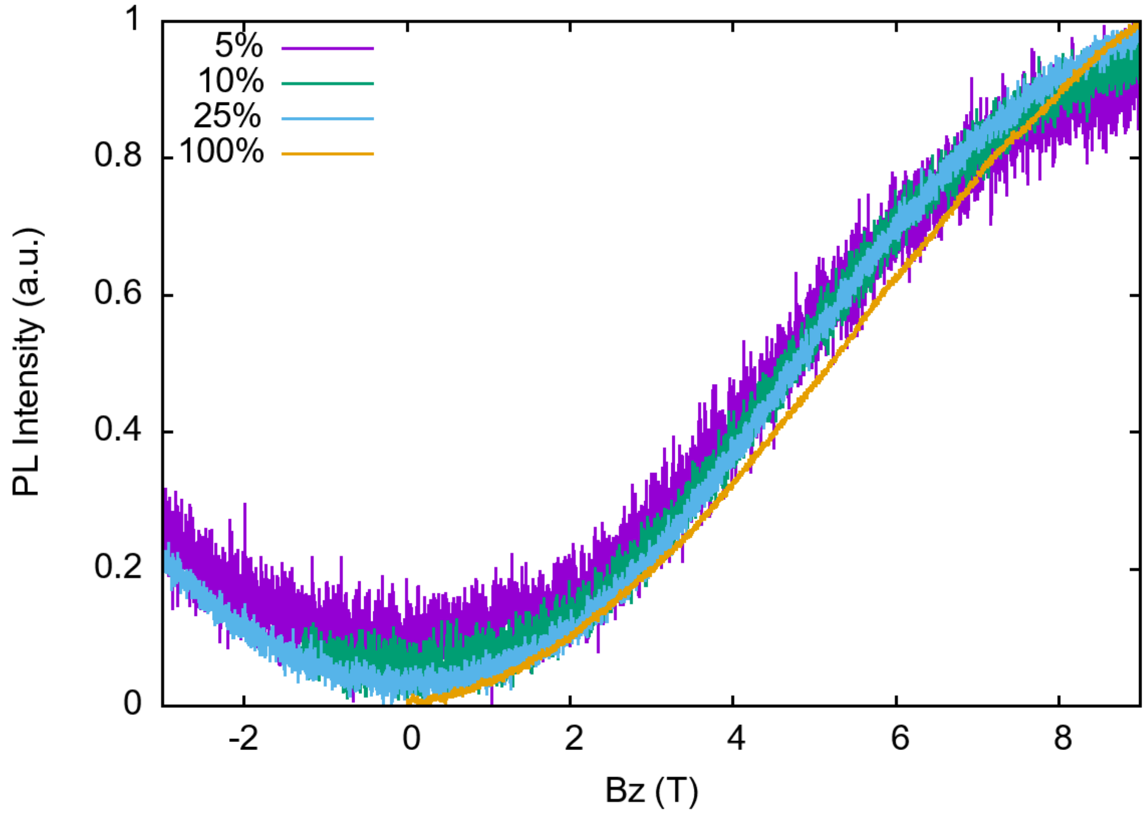

Figure 31: Laser power dependence of the MPL for M2TTM-3PCz-M2TTM. *Spectra were recorded from at 0.2K for a 0.1wt% M_2TTM-3PCz-M_2TTM doped polystyrene film excited using a 405nm laser excitation with varying laser power. The sample stage temperature was measured and PL measurements were conducted only after proper thermalisation of the sample stage.*

V. High Frequency Photoluminescence Detected Magnetic Resonance (HF-PLDMR)

We have set up HF-PLDMR experiments at microwave frequencies beyond 100 GHz. We have implemented evanescent amplified microwaves from whispering-gallery modes on a single crystal dielectric disk-resonator described in Methods and in earlier reports to study rotons in superfluid helium(*10*). Given in Figure 32 is the microwave transmission spectrum of the standalone resonator.

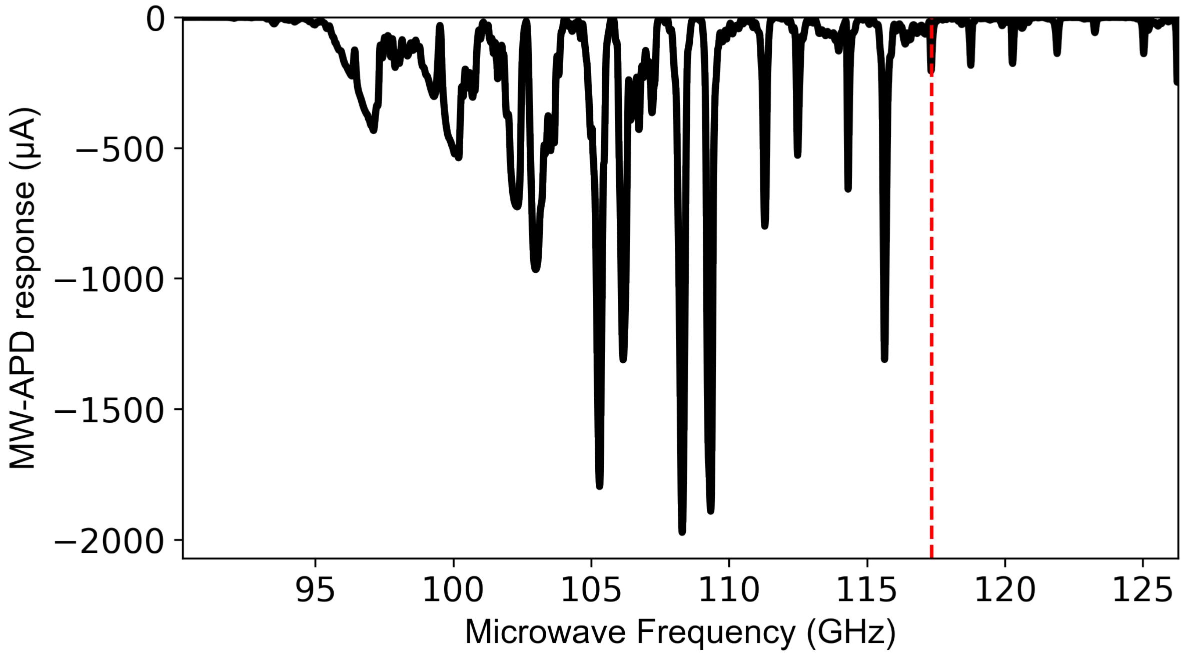

Figure 32: Microwave transmission spectrum of the standalone microwave dielectric resonator. *We are able to achieve a factor n = 9 amplification in frequency. For the current paper we choose the f_{MW} = 117.3115 GHz.*

The virtue of using high frequencies is the access to the fine structure that is otherwise inaccessible in X or Q-band techniques. Additionally at low temperatures the higher fields allow us to access a unique scenario: Complete spin polarisation in the T- state, so the ground state at $B_z > 0.65$ T is T_- and so the first $|\Delta m_s| = 1$ transition is the $T_- \rightarrow T_0$ which is accessed by the microwaves.

We first turn to analysing the PLDMR obtained at 117.3115 GHz from the full PL which is in the 600 – 750 nm range. This is shown in Figure 33.

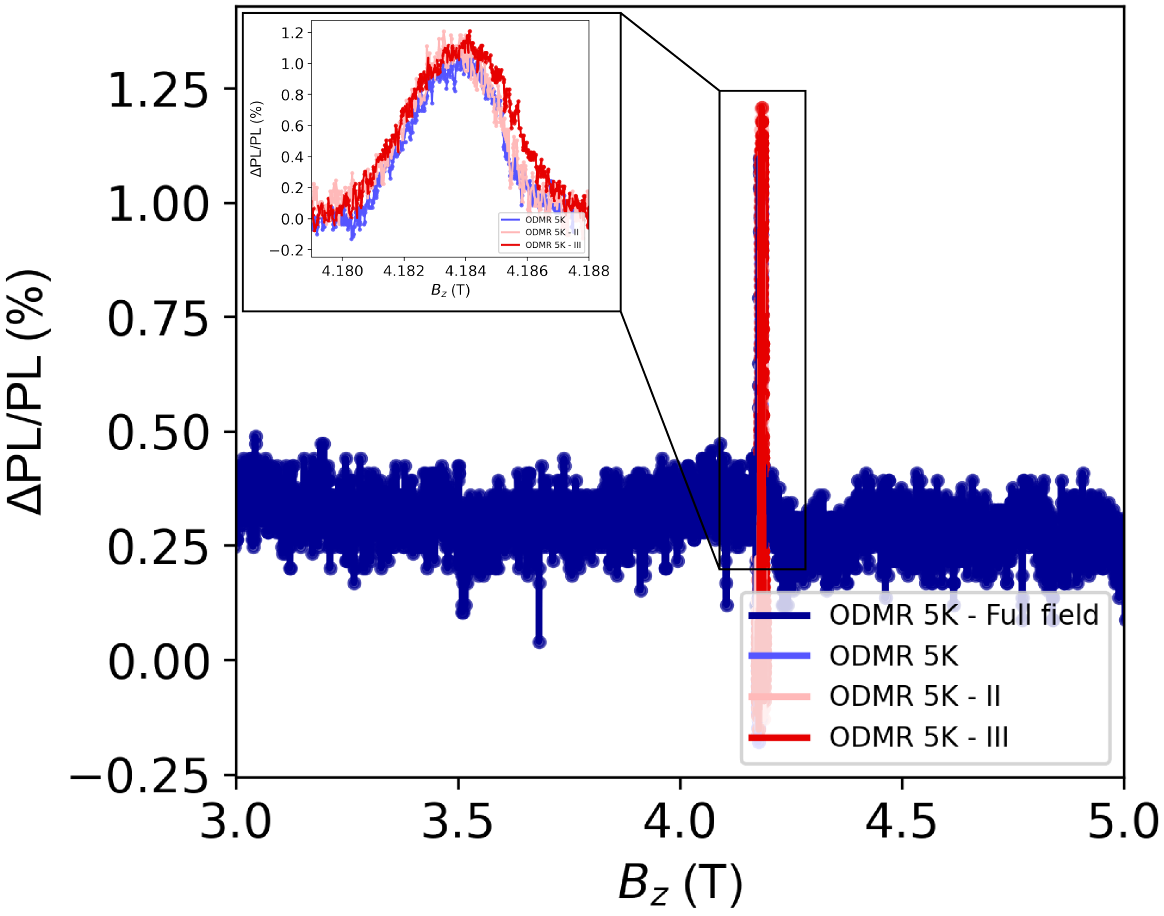

Figure 33: ODMR spectrum at 5 K in the 600 – 750 nm PL range with f_{MW} = 117.3115 GHz. *The experiment was repeated in up-field (ramp-up) and down-field (ramp-down) for each curve so in total the experiment was repeated 8 times. Each up-down field pair is labelled using roman numerals I-IV. (inset) Detailed scans at resonance B_z = 4.1835 T was performed using 0.0001 T steps. This confirmed the large contrast of ~1.2 % at 5 K.*

As we observe in Figure 33, a sharp positive PLDMR transition with a contrast of ~1.2% is observed at 4.184 T at 5K. In order to confirm this we ran multiple scans while ramping the magnetic field up and down to get independent data sets, which reproduced the data and established this as a real, albeit very large, PLDMR signal.

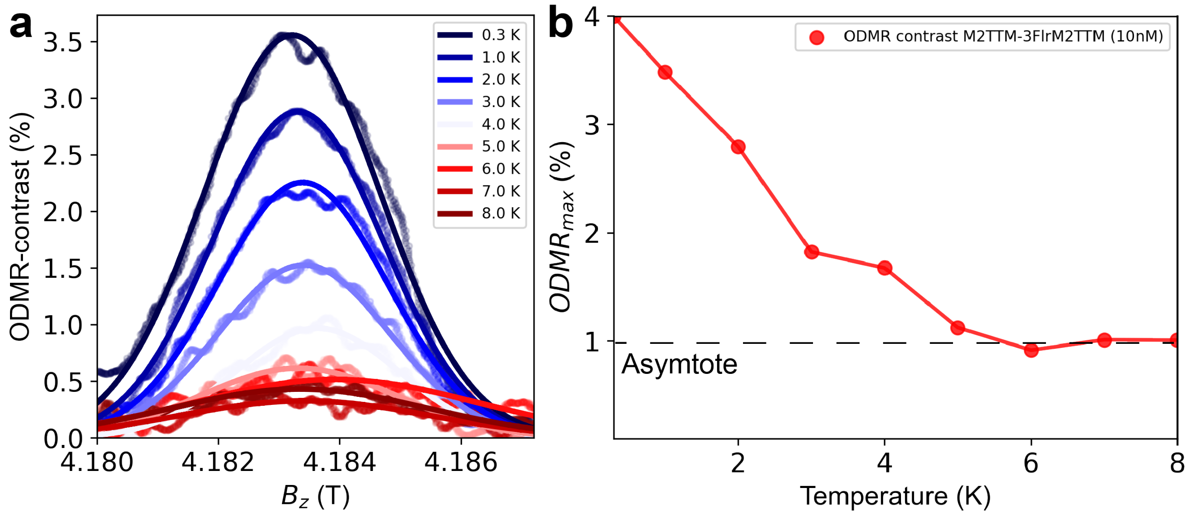

Figure 34: Temperature dependence of ODMR spectrum from 0.3K-8K in the 600-750 nm PL range with f_{MW} = 117.3115 GHz. ***(a)*** *The contrast scales with temperature, attaining an asymptotic value of ~1% by 5K.* ***(b)*** *The max-contrast plotted against temperature. In all cases a M_2TTM-3Flr-M_2TTM(10nM):PhCl3 crystal is used with a very small excitation fluence measuring 20 μA on the reference APD.*

To test the temperature dependence of this signal we ran the PLDMR experiment focussing on 4.180 T – 4.187 T region, shown in Figure 34, where the peak is contained. The temperature dependence and associated asymptotic stabilisation of the PLDMR contrast to ~0.5% at 5K indicates the origin of the signal being magnetization and spin based instead of thermal or bolometric.

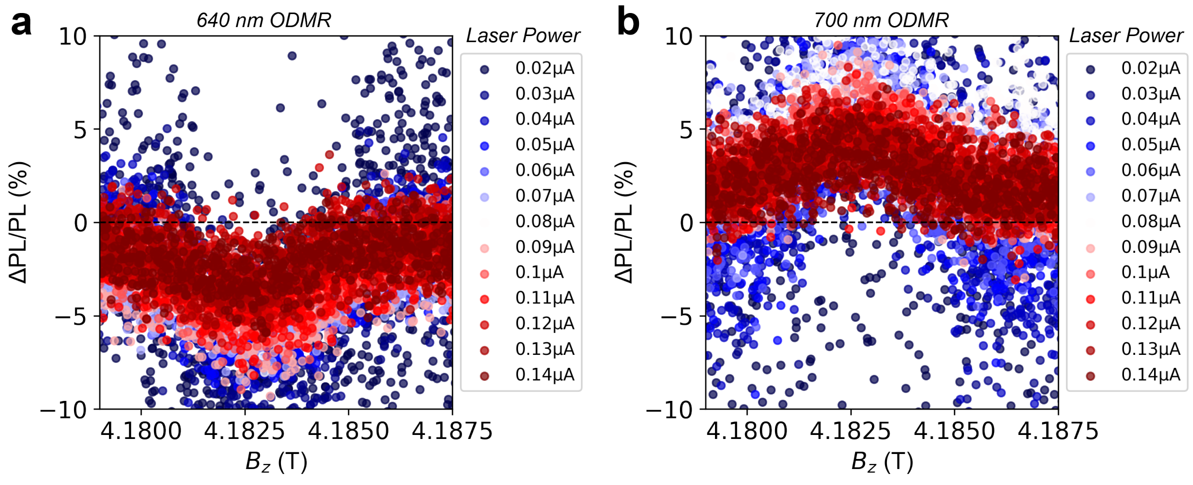

Figure 35: ODMR spectrum at 0.3K measured at different laser powers. *The data are recorded at* ***(a)*** *640 nm and* ***(b)*** *700 nm. The triplet emission experiences a bleach and the singlet feature experiences an enhancement with magnitudes ~15%. There is no laser power dependence indicating fluence independence of the signal. This also shows that the emergent triplet and singlet PLDMR signals are not originating from bimolecular or aggregation mediated bi/multimolecular states.*

Given that the triplet and singlet emit at distinct energies, 640 nm and 700 nm respectively, we perform wavelength resolved PLDMR to see if we can attribute them to separate dynamics. We find, as shown in Figure 35, the 640 nm PLDMR shows a negative contrast ~10% and the 700 nm PLDMR shows an equal and opposite positive contrast of ~10%. Due to the larger bandwidth of the singlet PL the difference between the two signals remains positive, but of

lower magnitude, this is the response we record when agnostic to PL wavelength. In Figure 35 we also show the scans performed at a plethora of fluences, which we measure using the value on the reference laser APD ranging from 0.02μA-0.14μA. The signal magnitude and sign is fluence independent which shows that it is not coming from any bimolecular or multimolecular process which would otherwise affect the PLDMR signal severely and would scale with fluence with a power law.

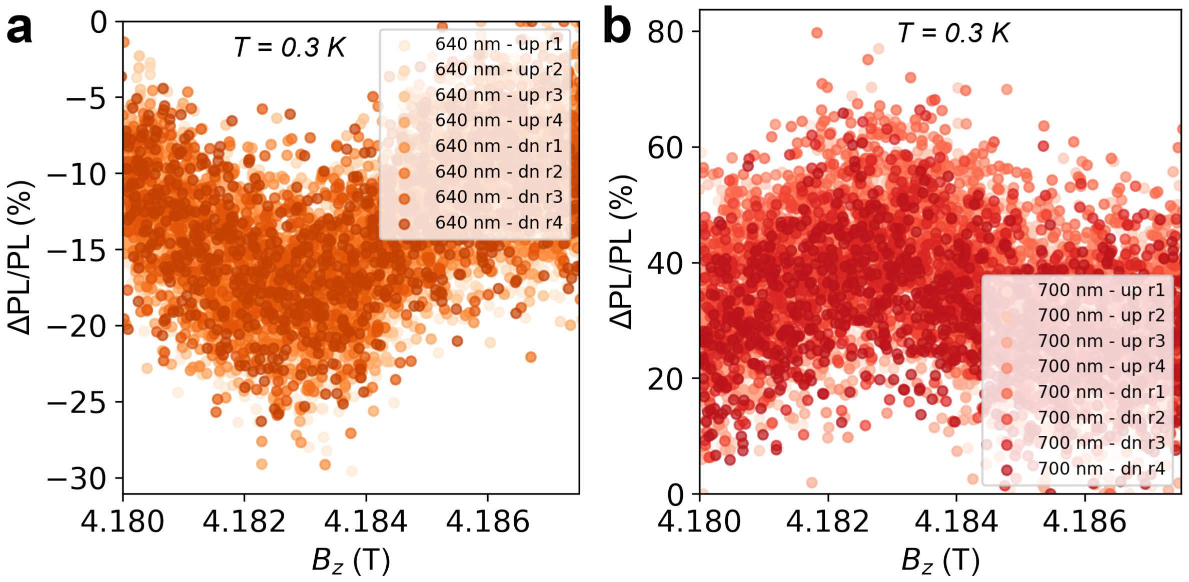

Figure 36: Repeats of spectrally resolved ODMR at 0.3K and low laser power. *Measurements performed on **(a)** 640 nm triplet PL and **(b)** 700 nm singlet PL show that we get nearly the same PLDMR contrast sign on every measurement performed, both while ramping up and down the magnetic field. This also precludes the ODMR arising from stored magnetic fluxes.*

To increase confidence in our data at separate wavelengths at the lowest temperature of 0.3K we repeated the field ramp-up and ramp-down sweeps at 640 nm and 700 nm grating positions multiple times which is shown in Figure 36, repeating the same contrast value every time with no indication of hysteresis.

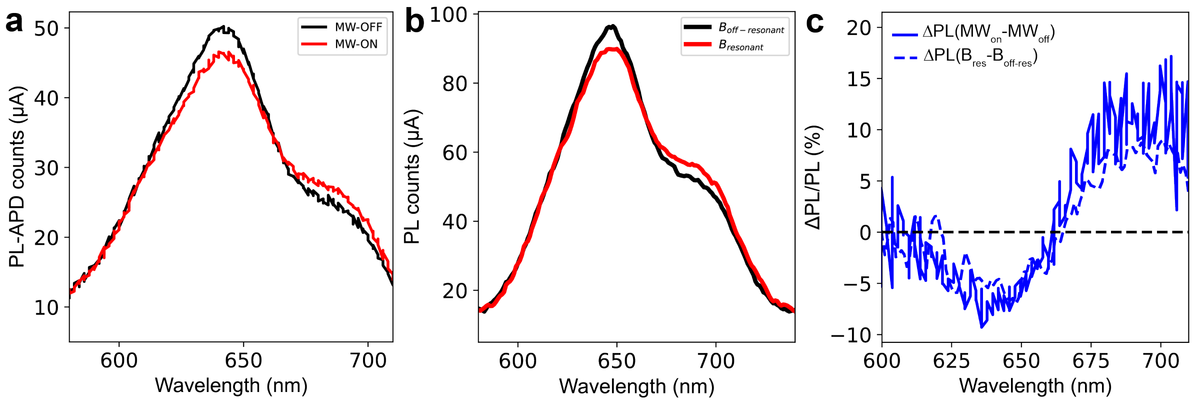

Figure 37: Spectrally resolved PLDMR. *We performed this in 2 ways, first **(a)** By homing on the resonance field position of 4.1835 T and then turning the microwaves on (red) and off (black), second **(b)** by keeping the microwave pulse on and then by measuring spectra at resonant magnetic field and off-resonant magnetic field (black). The key difference between (a) and (b) is that in (b), where the magnetic field is being ramped off-resonance, there is no possibility of heating at the sample area thus, confirming the equivalent response in microwave and magnetic field resonance helps us confirm that*

the PLDMR induced probe of spin polarisation arises from optical dynamic spin polarisation and not any thermal effects.

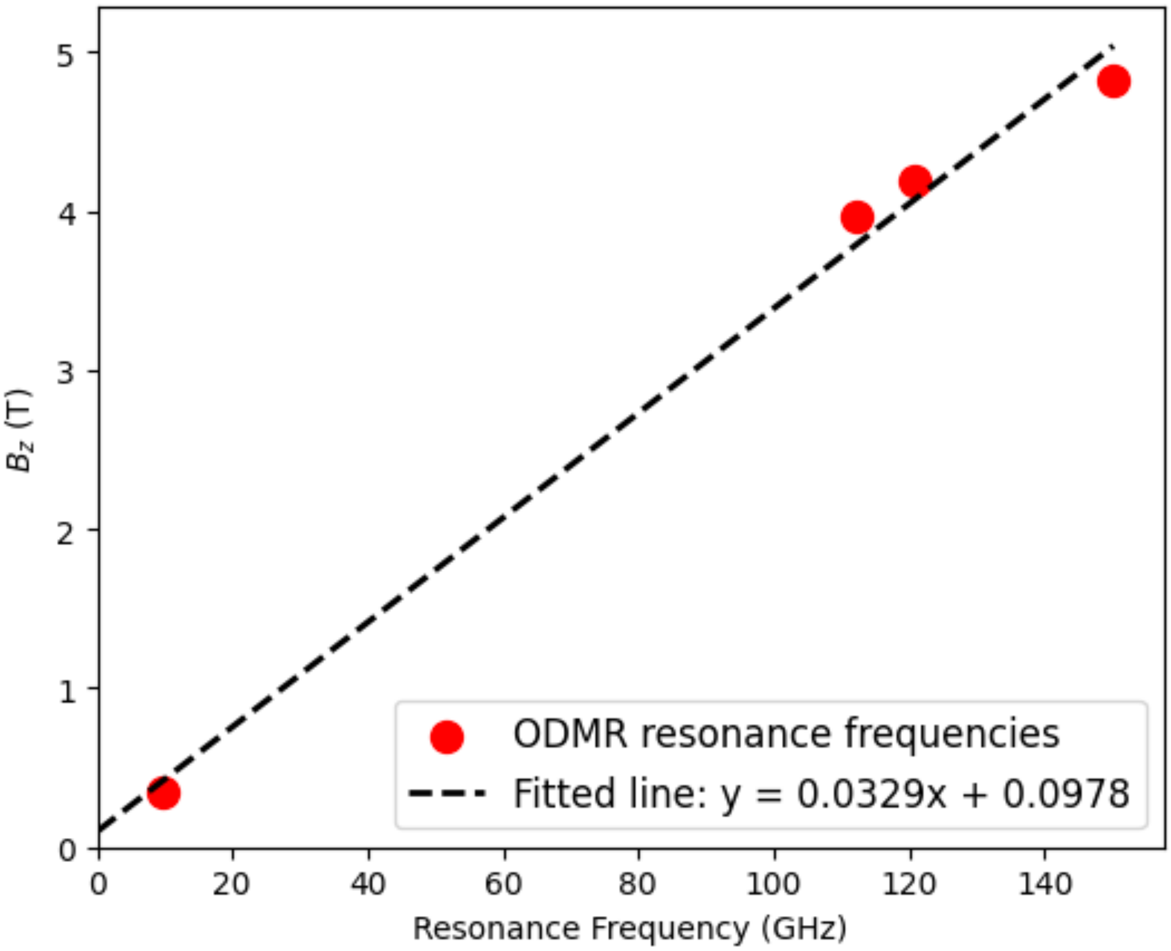

Figure 38: Zeeman shift in PLDMR. *In this figure we show the PLDMR resonance field for measurements carried out at different microwave frequencies. The Zeeman-shift linear fit is shown in black dashed lines.*

As described in the main text, we have spectrally resolved the PLDMR response at the resonance position. In the main text we showed the effect of turning the microwave pulse on or off at the resonant field position, also shown in Figure 37.a. This can also be done alternatively by keeping the microwave on continuously and applying a resonant and off-resonant magnetic field. This is shown in Figure 37.b, where we are able to recreate the same effect of the microwaves in sign and magnitude (the differential PL is shown in Figure 37.c) at the different spectral regions. We directly see in this data that the PLDMR drives population into the singlet state through ISC which reflects as an increased 700 nm at resonance, which is afforded by a bleach of the triplet population at 640 nm. A proposed mechanism is shown in Figure 39.

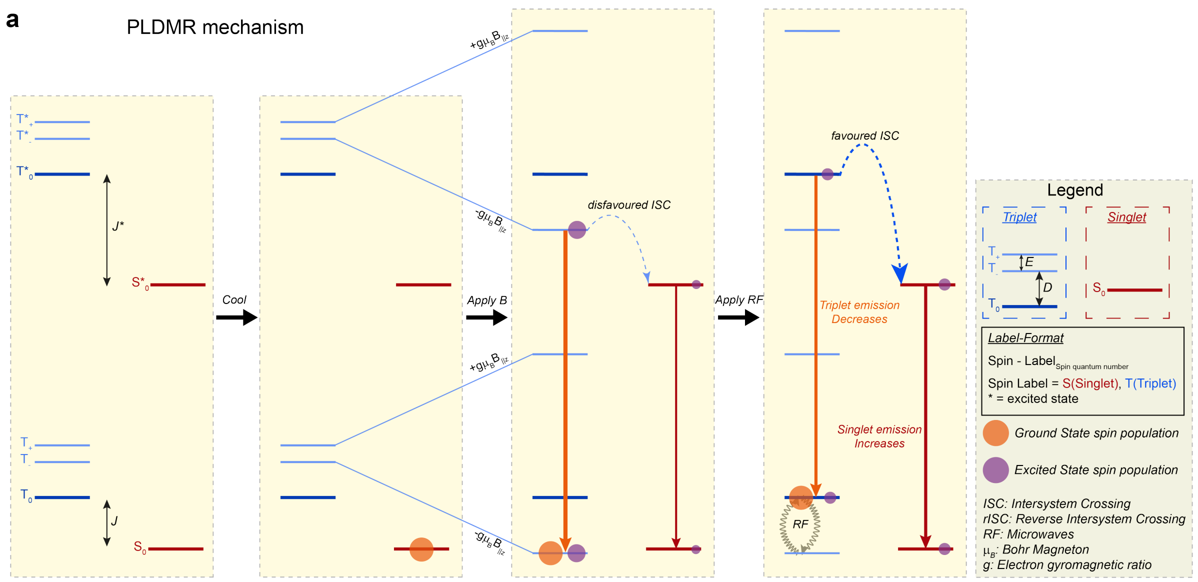

Figure 39: Possible mechanism for PLDMR. *(From left) When we cool down the M$_2$TTM-3Flr-M$_2$TTM molecule the molecule attains its ground state spin population, entirely polarised on the S_0 level, this is shown in Panel 2. Based on the magneto photoluminescence results we know that by applying fields beyond 0.65 T we stabilise the T_- level below the S_0 through the Zeeman energy, shown in Panel 3. This will shift the entire ground state spin population to T_-. Applying a microwave drive, shown in Panel 4, at the resonant condition will now drive the T_- population to the T_0 population. They key difference between the condition in Panel 3 and Panel 4 is the starting polarisation, which experimentally manifests in decreased triplet (640 nm) PL and increased singlet (700 nm) PL. This can be explained by a higher intersystem crossing rate from $T^*_0 \rightarrow S^*_0$ compared to the intersystem crossing rate from $T^*_1 \rightarrow S^*_0$. This reinforces the possibility of spin-selective ISC and subsequently rISC.*

VI. Transient Absorption Spectroscopy

Transient absorption studies were performed on the 0.1wt% doped polystyrene thin films of M$_2$TTM-3Flr and M$_2$TTM-3Flr-M$_2$TTM (Figure 40-41). The data for M2TTM-3Flr shows a photo-induced absorption (PIA) from the excited state in the visible and infra-red probe regions with monoexponential dynamics and no spectral evolution in any epoch. In stark contrast the spectra of the M$_2$TTM-3Flr-M$_2$TTM doped polystyrene films (Figure 40) show multiple growth and decay features in different spectral regions. The main PIAs of interest are centered at 520nm and 1240nm which we shall call PIA-2 and PIA-3 respectively. We observe that PIA-1 resembles the PIA observed from the monoradical M$_2$TTM-3Flr. PIA-1 decays fastest with a lifetime of 150 ps. This PIA, when deconvoluted contributes ~9% of the total population at room temperature as the associated growth in PIA-2 is only by 9-10%. While PIA-1 is decaying, PIA-2 grows in slowly within this time reaching its peak at 10ps after which it starts decaying. As PIA-2 decays, the final PIA-3 starts growing in, reaching its maximum population at 3 ns. Once the populations of PIA-2 and PIA-3 have equilibrated by 3 ns they both decay with biexponential kinetics with lifetimes 9.0 ns and 105.8ns which closely match the observed emission lifetimes. But this also indicates that the underlying states have convoluted absorption spectrum which we deconvolute through singular value decomposition (SVD) and Decay Associated Spectrum(DAS) analysis (Figure 40.d) to

show the distinct spectral components. Correlating the TRES and TA we can propose an excited state mechanism (Figure 40.e) where: The first excited state formed is thus the same in the monoradical and diradical. But within 400 ps a second state forms, this state emits at 640nm but also acts as a reservoir for a third slowly decaying state that emits at 700nm. Thus all the emission in the system comes out from the transiently evolved states in the excited state. From the nanosecond TA kinetics we observe no fluence dependence of these ns-decaying PIA features which is commensurate with our fluence independence in the PL kinetics.

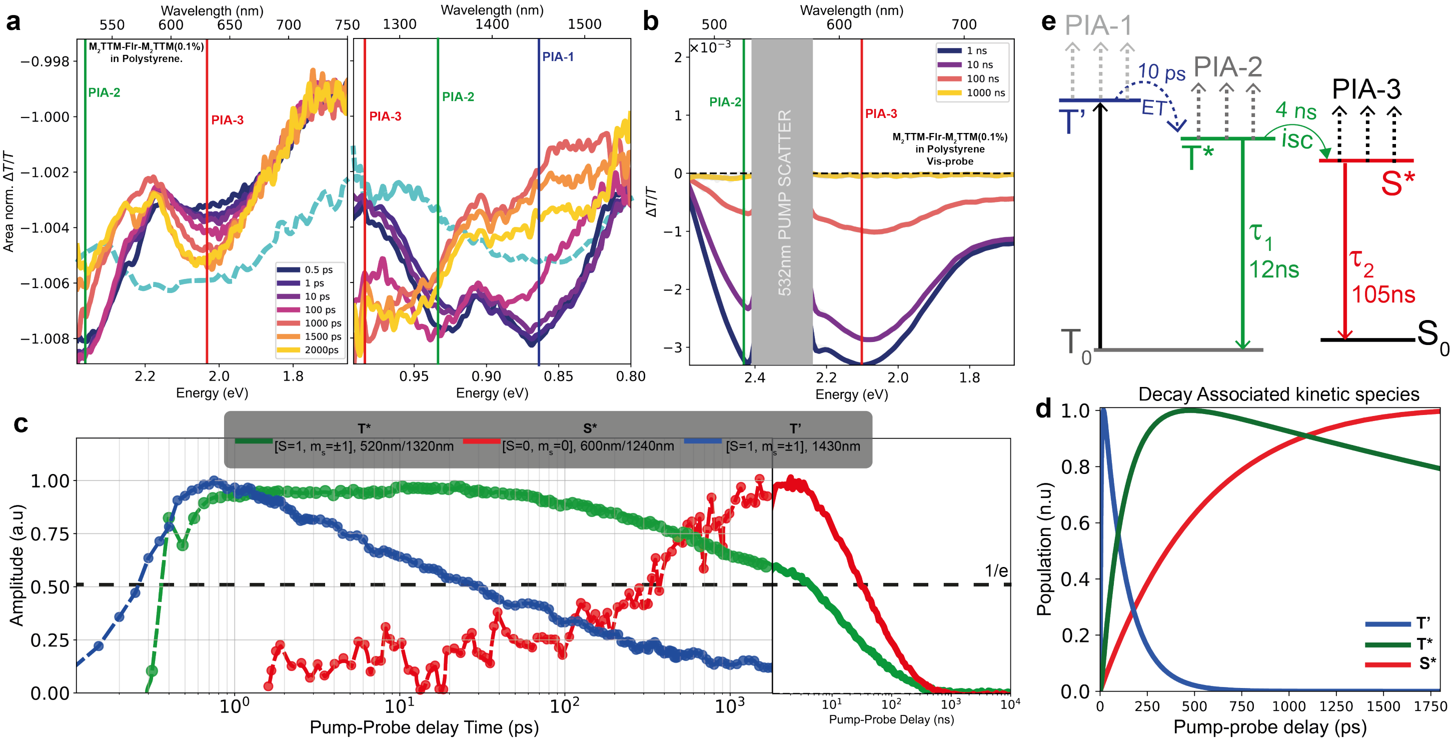

Figure 40: The different spectral and kinetic components from picosecond-nanosecond transient absorption. (a) *Spectral slices at select time intervals in the visible(left) and infra-red(right) probe regions. The temporally invariant spectrum of the M₂TTM-3FIr monoradical is shown in dashed blue lines for reference. Lines indicated as PIA-1, PIA-2 and PIA-3 indicate the regions of the spectrum varying with different kinetics attributed to different excited state species.* ***(b)*** *Spectral slices in nanosecond transient absorption with a visible probe.* ***(c)*** *The kinetic traces of PIA-1(green), PIA-2(red) and PIA-3(blue) are shown in the picosecond and nanosecond timescales, the picosecond-nanosecond break-point is 1.2ns. We observe that PIA-1 reseembles that of the monoradical and decays first, while it decays the PIA-2 feature grows in within 10ps and then starts decaying with a lifetime of 9.4ns, the PIA-3 feature grows in last and has a growth lifetime of 200ps, it also has the longest decay kinetics with a biexponential lifetime of 9.1ns and 104.2ns which resembles the delayed emission lifetime.* ***(d)*** *The Decay associated kinetic species deconvoluted from the full picosecond transient absorption showing 3 kinetic species.* ***(e)*** *The model that conforms to the observed spectral and kinetics observed in the transient absorption experiment. We assume that the first excited state T' is similar in electronic structure to the monoradical due to similarities in the PIA, followed by the second triplet CT state. The pump-scatter at 532nm has been omitted from the plot. The final state formed is a singlet CT state with a smaller optical gap with the ground state as evidenced by time resolved photoluminescence studies.*

Area normalisation is performed using the equation $\left(\frac{f(t,\lambda)}{\int_0^\lambda f(t,\lambda)d\lambda}; f(t,\lambda) = \frac{\Delta T(t,\lambda)}{T(t,\lambda)}\right)$

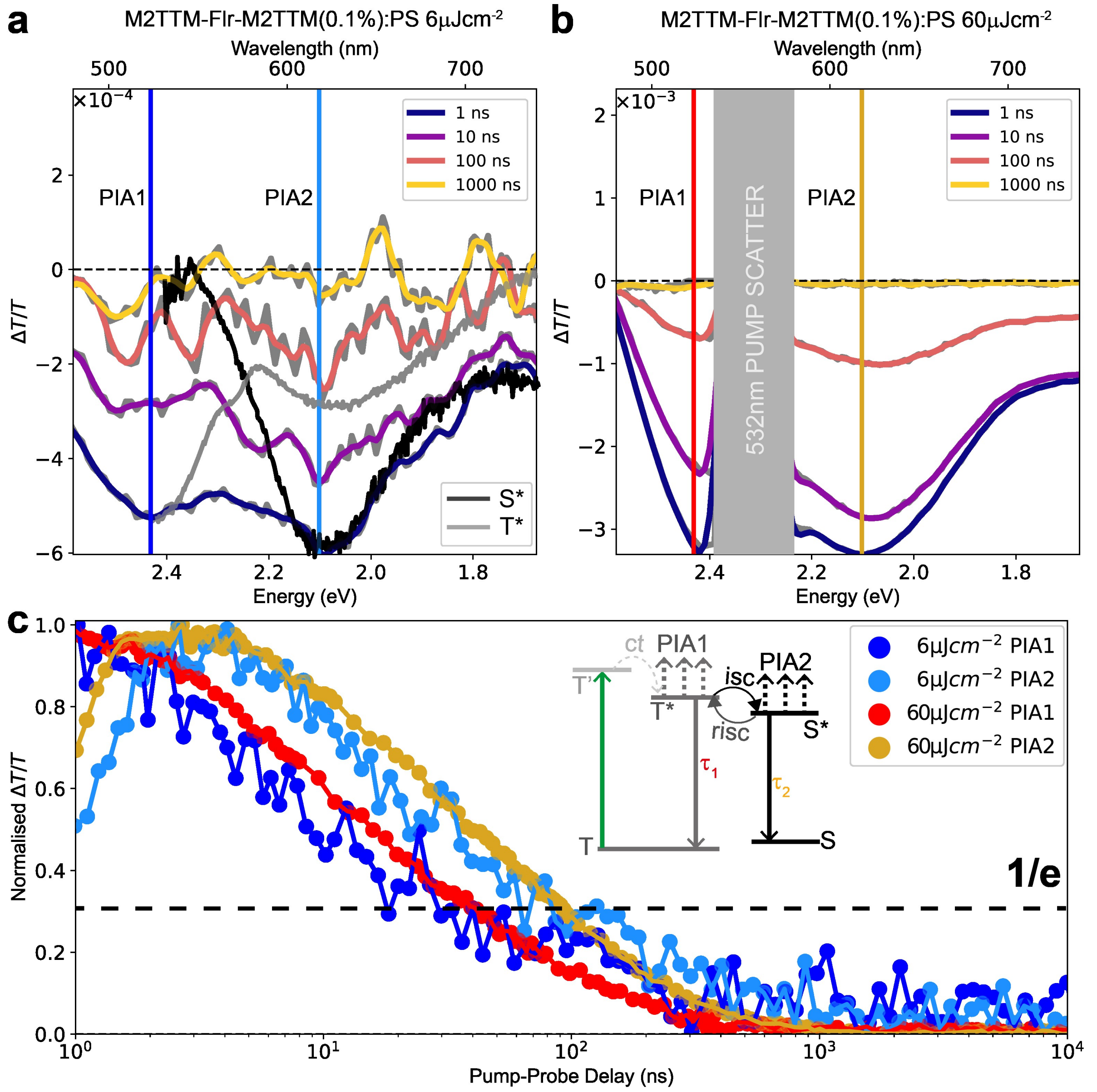

Figure 41: Fluence dependent nanosecond visible probe transient absorption spectrum. ***(a)*** *Spectral slices at 6μJcm*$^{-2}$ *fluence and* ***(b)*** *Spectral slices at 60μJcm-2. The decomposed prompt(grey) and delayed(black) components from our previous SVD analysis of the picosecond transient absorption spectra is shown in (a) for reference. Slices are presented at 1, 10, 100, 1000 ns. In* ***(c)*** *we show the kinetics recorded for the sample at the 2 fluences. We observe that the 550nm PIA is faster than the 620nm PIA for all fluences with no observable modulation in kinetics with 10x increase in fluence. Spectra were recorded at quoted fluences with a 532nm excitation on thin films of a 0.1wt% M*$_2$*TTM-3FIr-M*$_2$*TTM in Polystyrene*

VII. SQUID Magnetometry

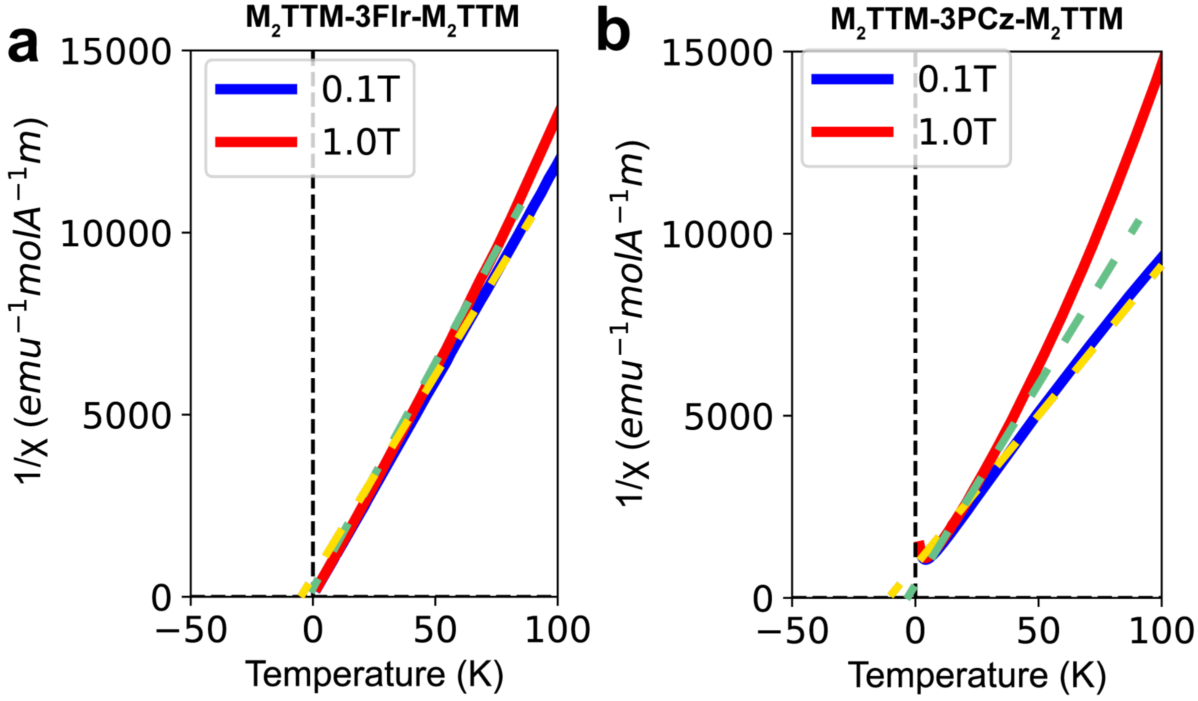

Figure 42: Curie Plots for magnetometry on pure diradical powders. ***(a)*** *M_2TTM-3Flr-M_2TTM and* ***(b)*** *M_2TTM-3PCz-M_2TTM. The data were recorded at 0.1T(blue) and 1.0T(red). The Curie-Weiss plots at the low temperature, linear limit are shown in yellow and green dashed lines for fits corresponding to 0.1T and 1.0T respectively.*

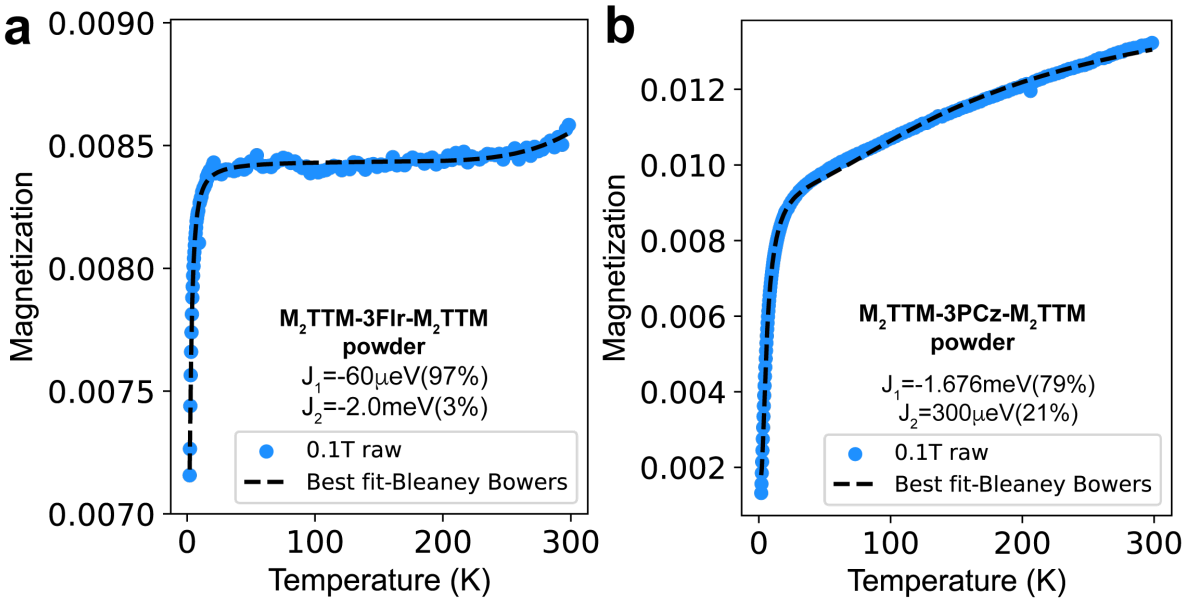

Figure 43: Bleaney-Bowers fits for magnetometry on pure diradical powders. *χT vs T plots for* ***(a)*** *M_2TTM-3Flr-M_2TTM and* ***(b)*** *M_2TTM-3PCz-M_2TTM. The data were recorded at 0.1T(blue). The Bleaney-Bowers model fits for each material is shown in black dashed lines. The parameters for the fitting and their relative weights are quoted in the figure.*

VIII. Pulsed Electron Spin Resonance

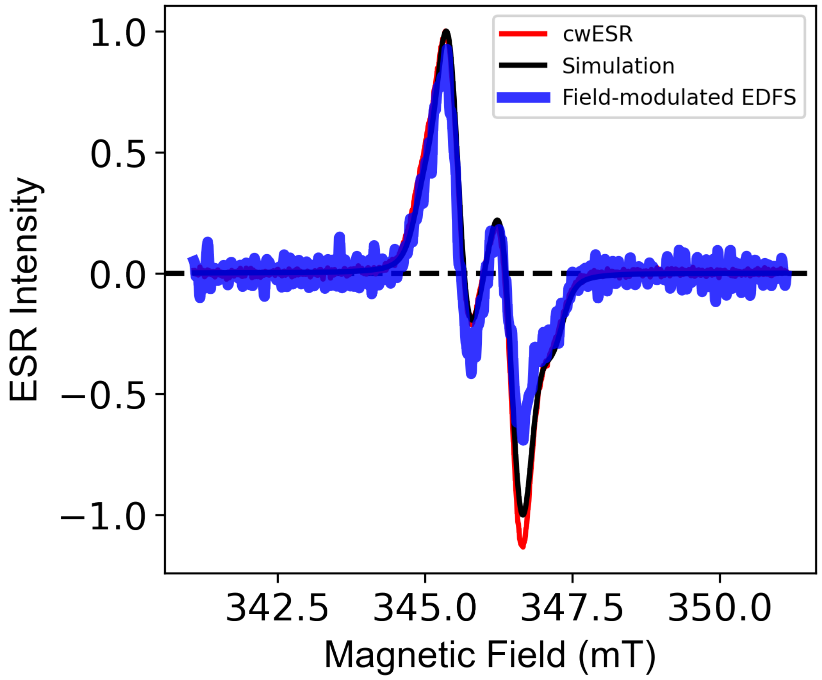

Figure 44: Overlay of pseudo field-modulated EDFS over the cwESR spectrum and the simulation of the cwESR spectrum. *All measurements agree with one another with no peak shifts across techniques. The measurements are performed at 300K in the dark on 0.1% polystyrene films containing M_2TTM-3Flr-M_2TTM.*

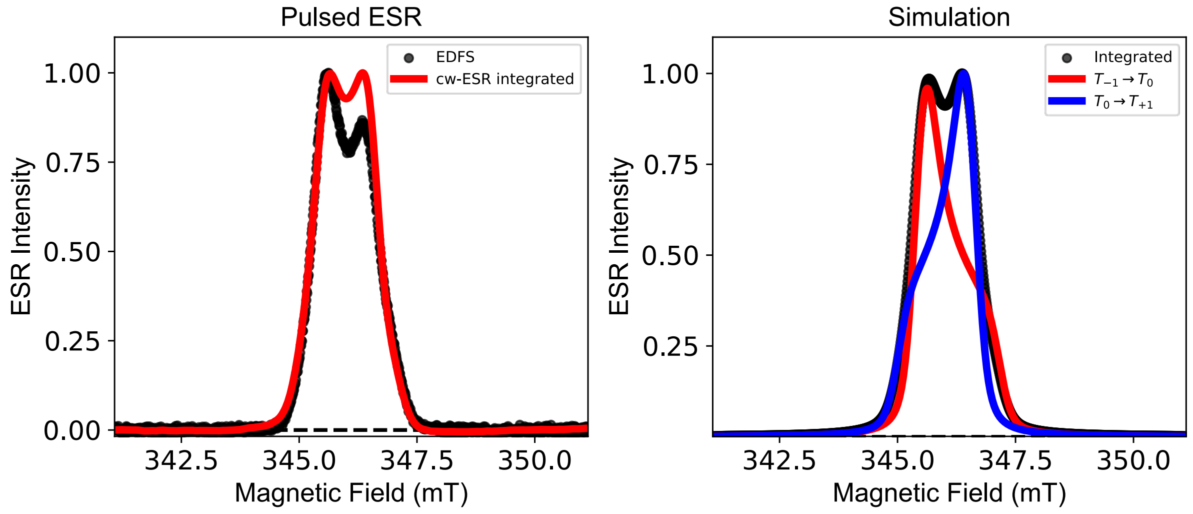

Figure 45: Spectral Agreement: (left) *Overlay of the EDFS spectrum (black) and the numerically integrated cwESR spectrum (red).* ***(right)*** *Simulation components corresponding to the $T_{-1} \rightarrow T_0$ transition (red) and $T_0 \rightarrow T_{+1}$ (blue) are overlaid on the integrated cwESR spectrum (black). The measurements are performed at 300K in the dark on 0.1% polystyrene films containing M_2TTM-3Flr-M_2TTM.*

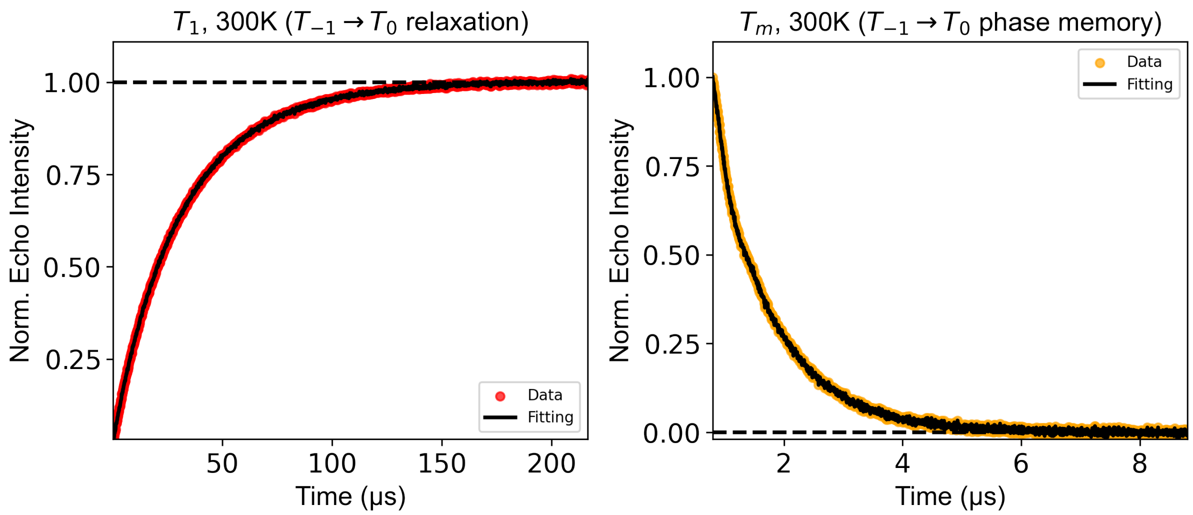

Figure 46: Decoherence of the T-1 → T0 transition. (left) *The T_1 experiment data (red) and the fitted growth curve (black).* ***(right)*** *The T_m experiment data (yellow) and the fitted decay curve (black). The black dashed lines are the biexponential fits. From the best-fit curves we obtain T_1 = 31.7 ± 0.1 μs and T_m = 950 ± 3 ns. The measurements are performed at 300K in the dark on 0.1% polystyrene films containing M_2TTM-3Flr-M_2TTM.*

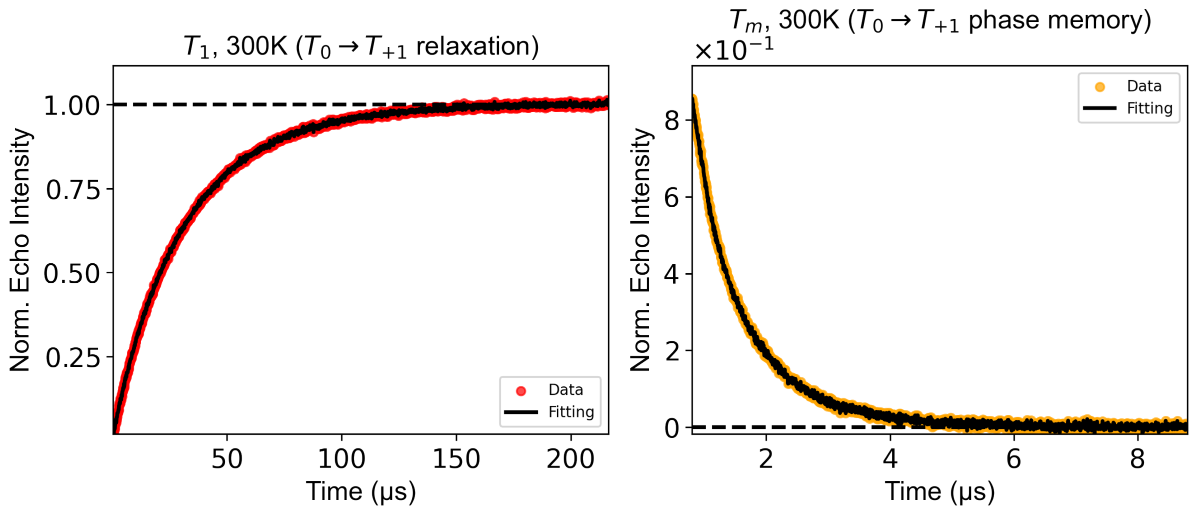

Figure 47: Decoherence of the T0 → T+1 transition. (left) *The T_1 experiment data (red) and the fitted growth curve (black).* ***(right)*** *The T_m experiment data (yellow) and the fitted decay curve (black). The black dashed lines are the biexponential fits. From the best-fit curves we obtain T_1 = 31.8 ± 0.1 μs and T_m = 827 ± 4 ns. The measurements are performed at 300K in the dark on 0.1% polystyrene films containing M_2TTM-3Flr-M_2TTM.*

IX. Transient Electron Paramagnetic Resonance

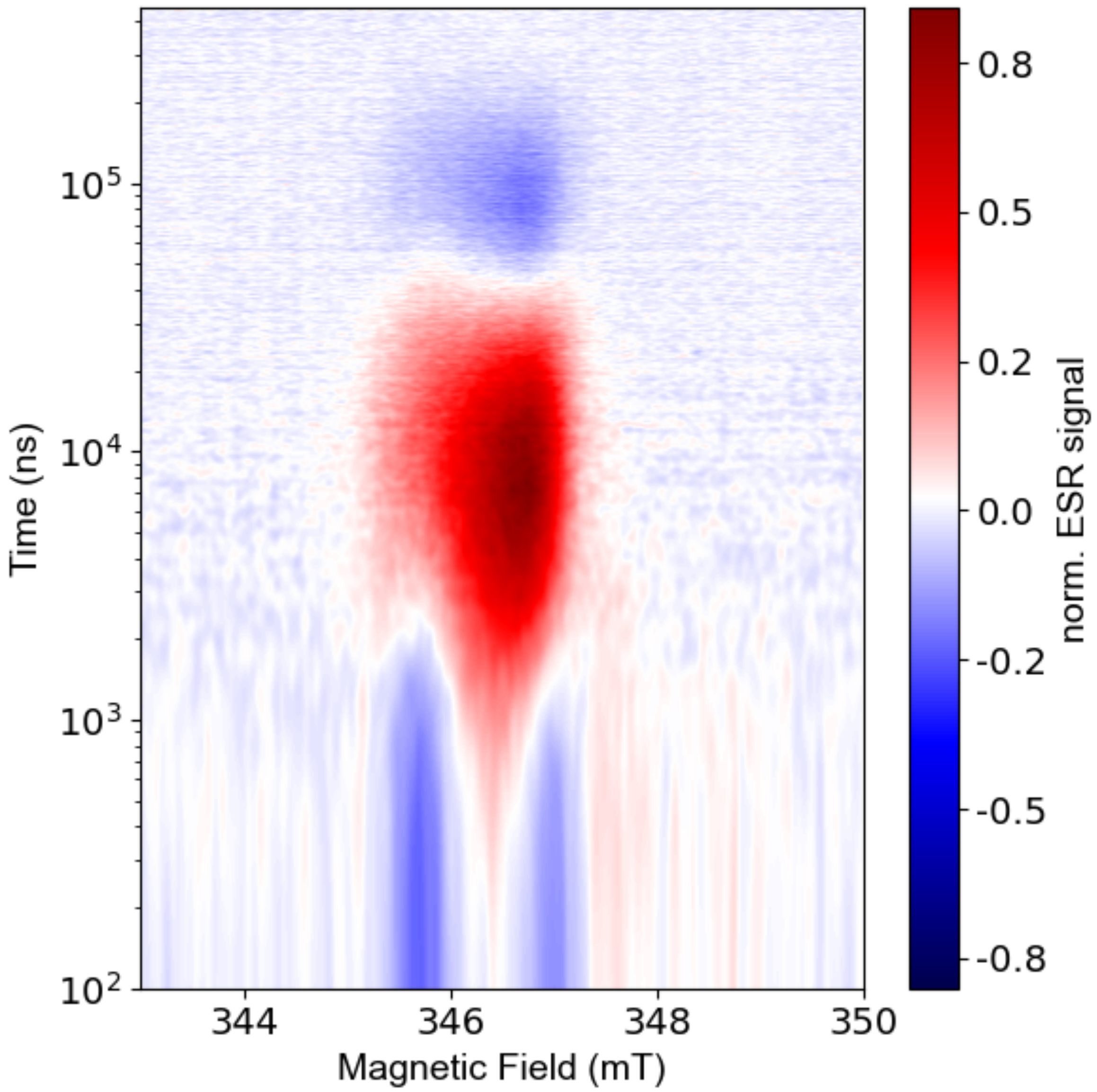

Figure 48: TrESR heatmap of M2TTM-3FIr-M2TTM (0.1%): Polystyrene films. *A 532 nm excitation of 5 ns was used with repetition rate of 100 Hz.*

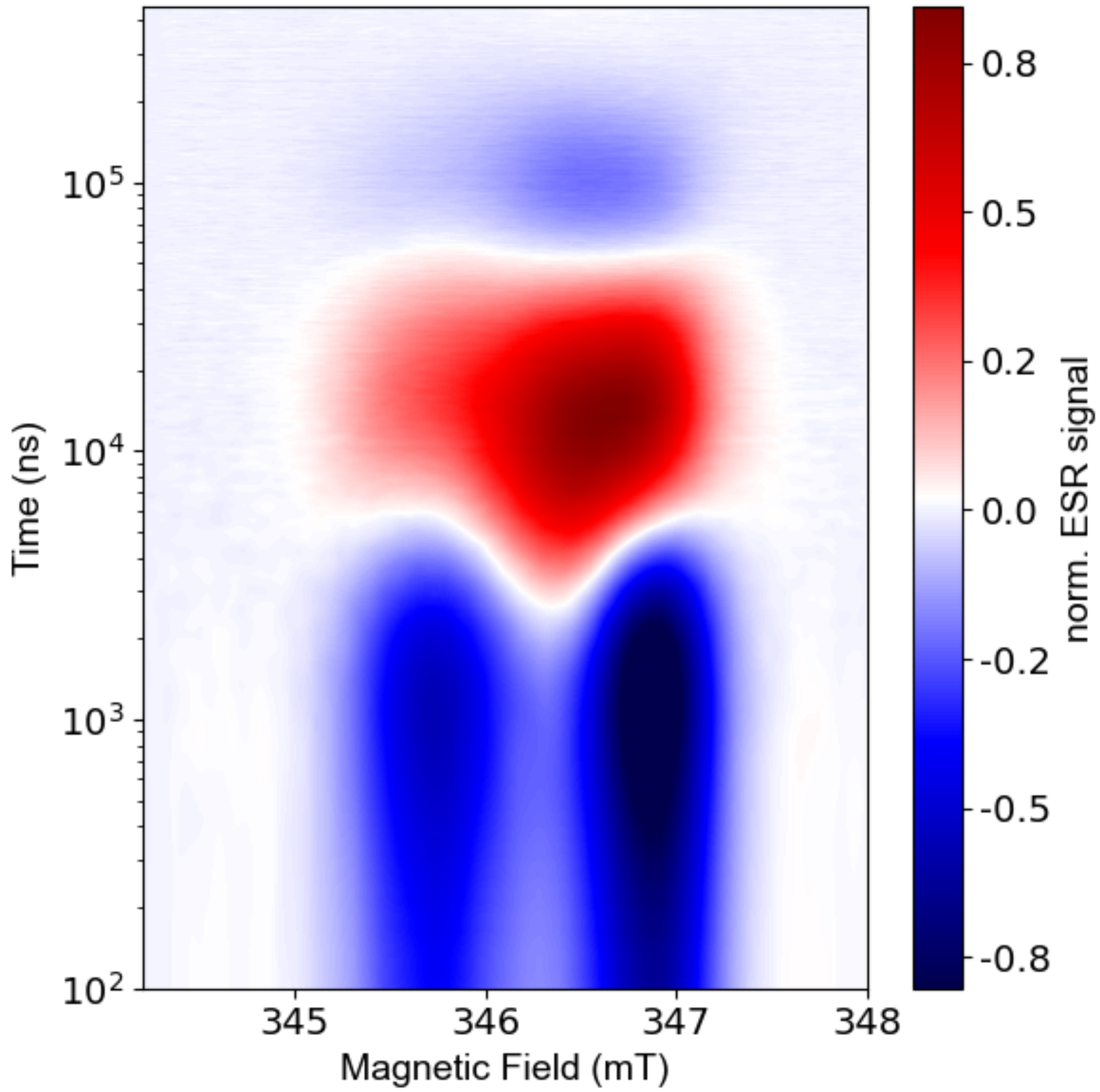

Figure 49: TrESR heatmap of frozen 50 mM solutions of M2TTM-3FIr-M2TTM in Toluene.
A 532 nm excitation of 5 ns was used with repetition rate of 100 Hz.

<u>CW ESR spectrum simulation parameters:</u>

g = 2.0029, 2.0025 (axial g tensor)

D = 30.3 MHz, E = 1.57 MHz

Linewidth peak to peak (Voigtian), Gaussian component ~0.3mT and Lorentzian ~ 0.11 mT

Boltzmann population

<u>Transient ESR spectrum beyond 50 μs simulation parameters:</u>

g = 2.0029, 2.0025 (axial g tensor)

D = 30.3 MHz, E = 1.57 MHz

Linewidth (Voigtian), Gaussian component ~0.55mT and Lorentzian ~ 0.05 mT

Non-Boltzmann population $p(T_+) > p(T_0) = p(T_-)$.

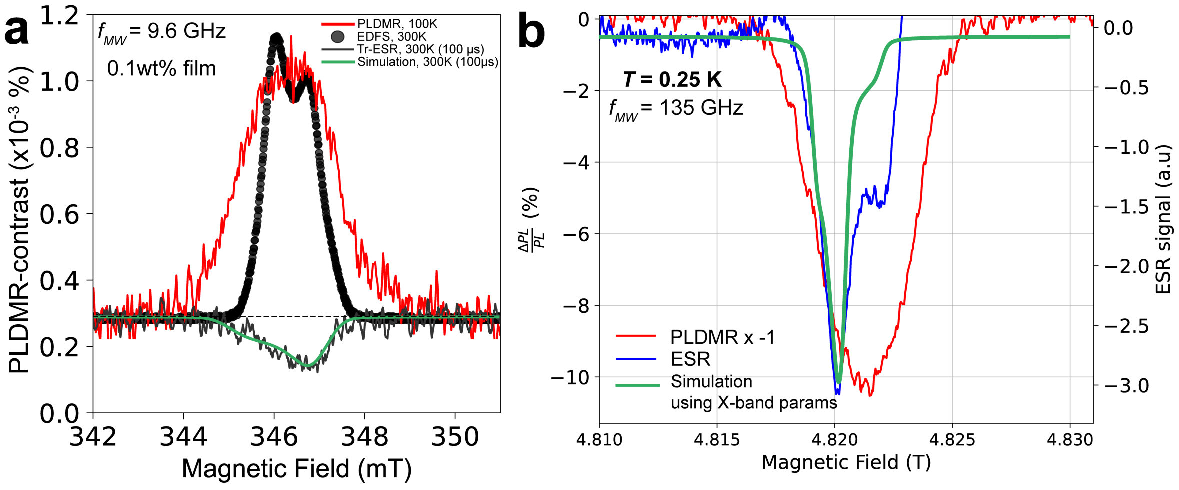

Figure 50: Different microwave resonance spectroscopies overlaid: **(a)** X-band CW ODMR (100K), CW EPR (300K) and pulsed EDFS (300K); **(b)** PLDMR and EPR at 0.25K measured at 135GHz microwave excitation. The simulation of the ESR spectrum at 135GHz using X-band parameters is shown in green.

X. Quantum Chemical Modelling

X.1 The monoradical.

The doublet ground-state geometry of the M_2TTM-3Flr monoradical was optimized at the density functional theory (DFT) level in the unrestricted formalism with the M06-2X functional(*11*), combined with the 6-31G(d,p) basis set. In the optimized structure the fluorene-TTM dihedral angle is ~37º (Figure 51).

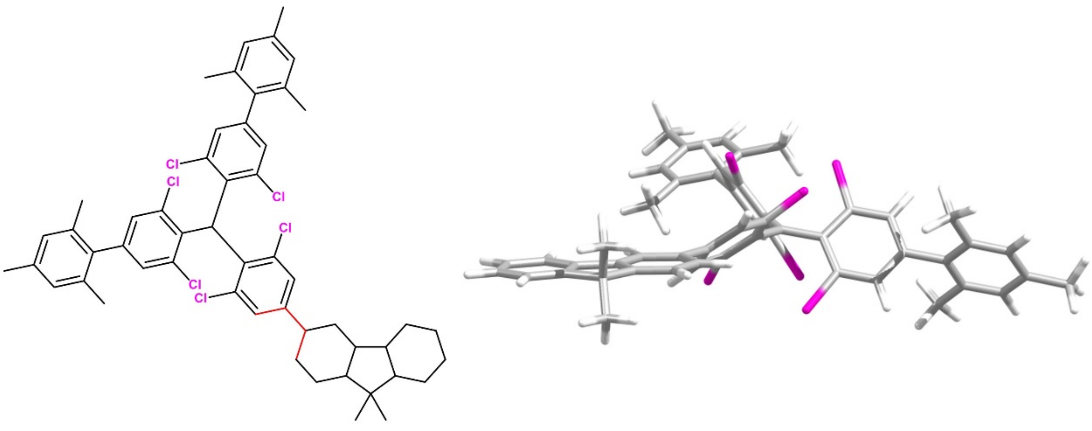

M_2TTM-3Flr

Figure 51. Chemical representation and optimized ground-state geometry of the M_2TTM-3Flr monoradical, where the fluorene-TTM dihedral angle is highlighted in red.

Time dependent (TD) DFT calculations were carried out by using the *screening* range-separation hybrid (SRSH) approach, with the LC-ωhPBE/6-31G(d,p) level of theory, where the range-separation parameter ω was optimally tuned at 0.100 $Bohr^{-1}$ and the dielectric constant ε was set at 2.5. Low-lying D_1 and D_2 excited states of the M_2TTM-3Flr monoradical are predicted at 2.60 and 2.78 eV with oscillator strengths of 0.039 and 0.021, respectively. Interestingly, the presence of the fluorene breaks the degeneracy between D_1 and D_2 as found in C_3 substituted TTM derivatives(*12*). Actually, a rigid scan along the fluorene-TTM dihedral angle (Figure 52) reveals that, while the excitation energy of D_2 remains constant irrespective of the conformation, the D_1 energy is largely affected by the dihedral angle value, displaying a large red shift when going towards planarity. Close to 90º, where the π-conjugation between the fluorene and TTM is null, D_1 and D_2 are quasi-degenerate, similar to the case of the TTM fragment. This indicates the presence of fluorene-TTM mixing in D_1 but not in D_2. In fact, D_1 is largely stabilized by the mixing with configurations where the fluorene contributes, in particular with charge-transfer (CT) excitation from the fluorene HOMO to the TTM SUMO. Additionally, the scan predicts a minimum of D_1, close to 17º with a relaxation energy of 0.32 eV. This is confirmed by a full geometry optimization of the D_1 state that shows a relaxation energy of 0.34 eV and an optimal dihedral angle of 16º. Thus, relaxation at the excited-state level is expected to be accompanied by a change in the torsion angle towards a more planar structure. We expect these conformational relaxation effects to drive the red shift experimentally observed when going from the mono- to the diradical.

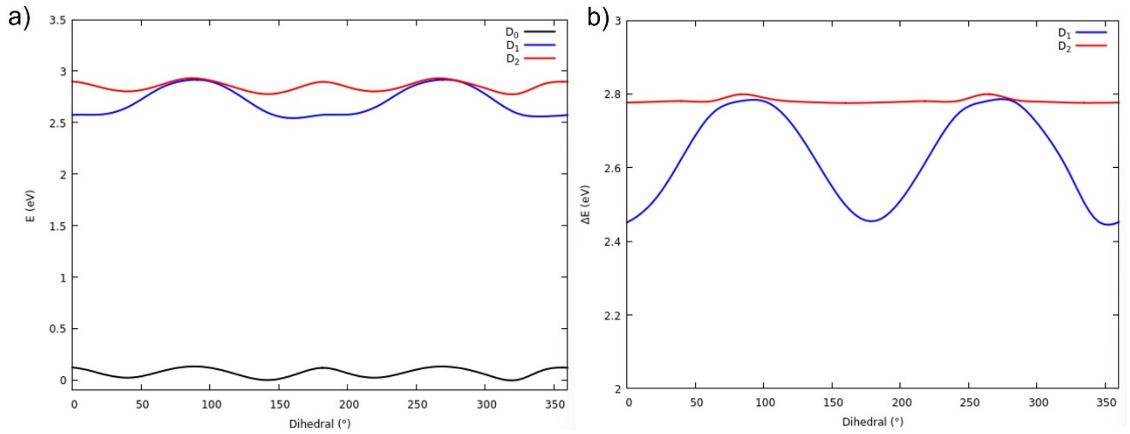

Figure 52: Rigid scan along the fluorene-TTM dihedral angle for the M2TTM-3Flr monoradical. (a) *Absolute energies including the ground state D_0.* ***(b)*** *Vertical excitation energies.*

X.2 The diradical.

In the optimized triplet and singlet (broken-symmetry) ground-state geometries, the two dihedrals at ~37° yielded quasi isoenergetic (+,+) (with near to C_2 symmetry) and (+,-) configuration (with C_1 symmetry) (Figure 53). For the M_2TTM-3Flr-M_2TTM

diradical there are no significant differences between the optimized singlet and triplet geometries at the ground state, with the singlet-triplet gap smaller than 1 meV.

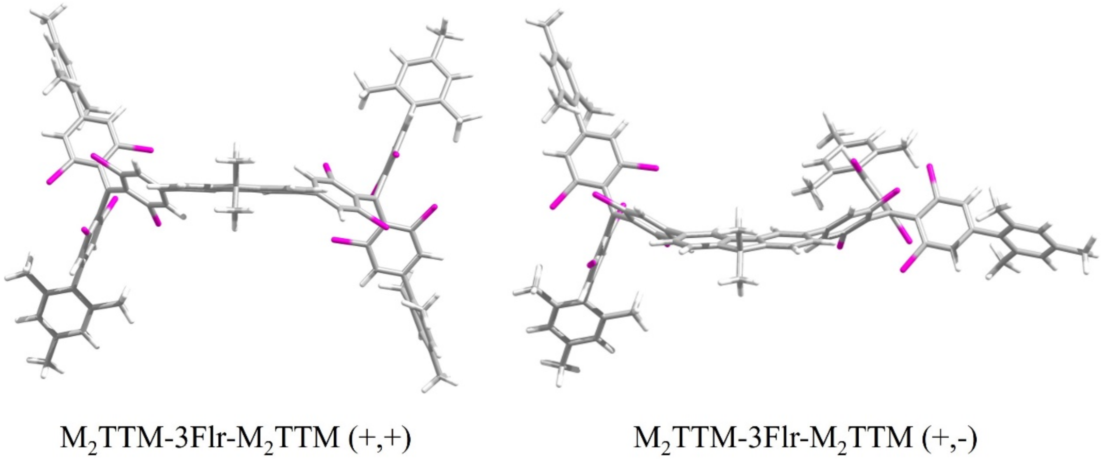

Figure 53. Chemical representations and optimized ground-state (+,+) and (+,-) geometries of the M_2TTM-3Flr-M_2TTM diradical.

We next performed Tamm-Dancoff approximation-based (TDA) TDDFT calculations on the TTM-3Flr-TTM diradical (where mesityl groups were omitted to speed up the calculations) at the OT-SRSH LC-ωhPBE/cc-pVDZ level of theory (ω = 0.104 Bohr^{-1}) as a function of the dielectric constant ε. For each value of ε (1, 2, 2.37, 3, 4) we computed the triplet and singlet vertical excitation energies, i.e., at the Frank-Condon region, distinguishing between the zwitterionic (ZI) and CT pair states, as shown in Figure 54. While the ZI states (in red and blue) lie at higher energy than the ^{1}CT states in gas-phase, for a sufficiently large dielectric constant (ε > 2) the ZI states become the lowest lying excited states. In fact, as the ZI state involves a charge transfer from the SOMO of one TTM to the SUMO of the other, it is very sensitive to dielectric screening effects. At ε = 2 vertical excitations from the ground state generates hybrid singlet states with mixed CT and ZI character. For all the investigated values of the dielectric constant, the singlet ^{1}CT states and triplet ^{3}CT state are very close in energy (within 30 meV) and, as soon as the dielectric field effect is turned on at ε > 1, their energy values slightly change. In addition, the calculated (vertical) exchange energy evolves between 30 and 240 meV in a narrow range of dielectric fields (2 < ε < 3).

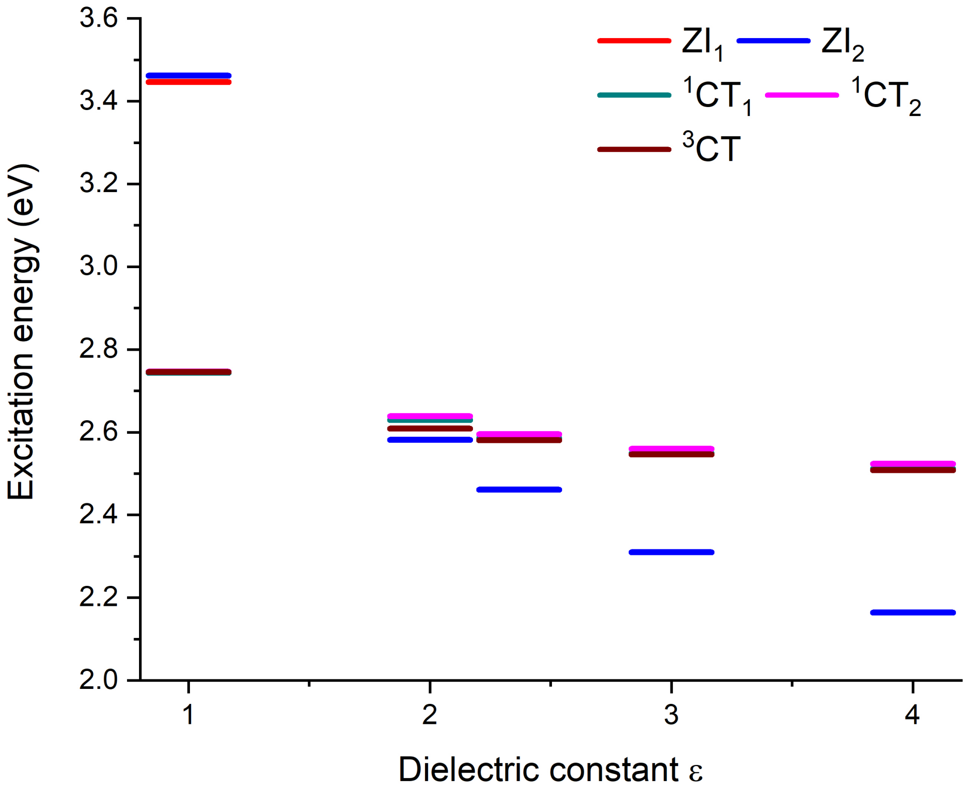

Figure 54: Vertical excitation energies from the Frank-Condon region of triplet (^{3}CT), singlet (^{1}CT) and zwitterionic (ZI) states as a function of the dielectric constant. The TDA TDDFT calculations were performed at the OT SRSH LC-ωhPBE/cc-pVDZ level of theory.

Figure 55-63 show the natural transition orbitals (NTOs) of the relevant transitions as computed in gas-phase (at ε=1), at ε=2, and at ε=2.37, typical of toluene. Vertical excitation energies and the oscillator strength (f) of a given transition are reported as well, along with the ZI contribution (in %).

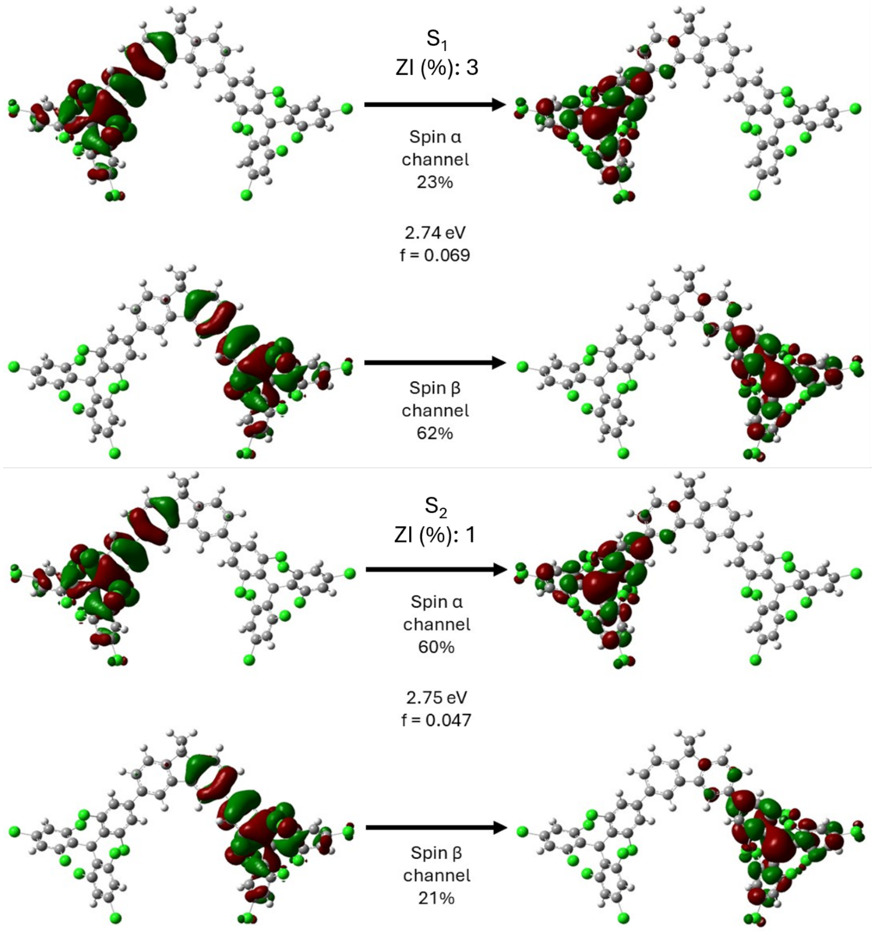

Figure 55: Hole-particle natural transition orbitals of S1 and S2 ($^1CT_{1/2}$) as computed at the Frank-Condon region in gas-phase.

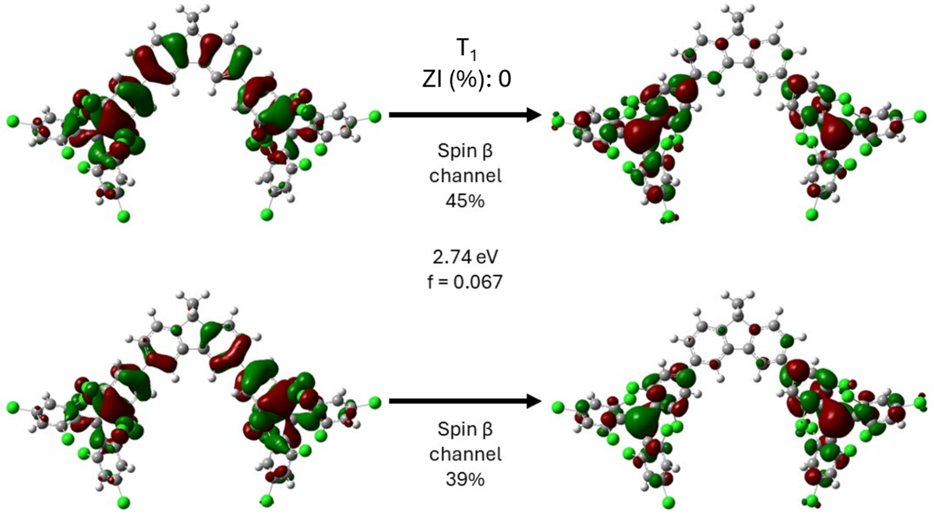

Figure 56: Hole-particle natural transition orbitals of T1 (^{3}CT) as computed at the Frank-Condon region in gas-phase.

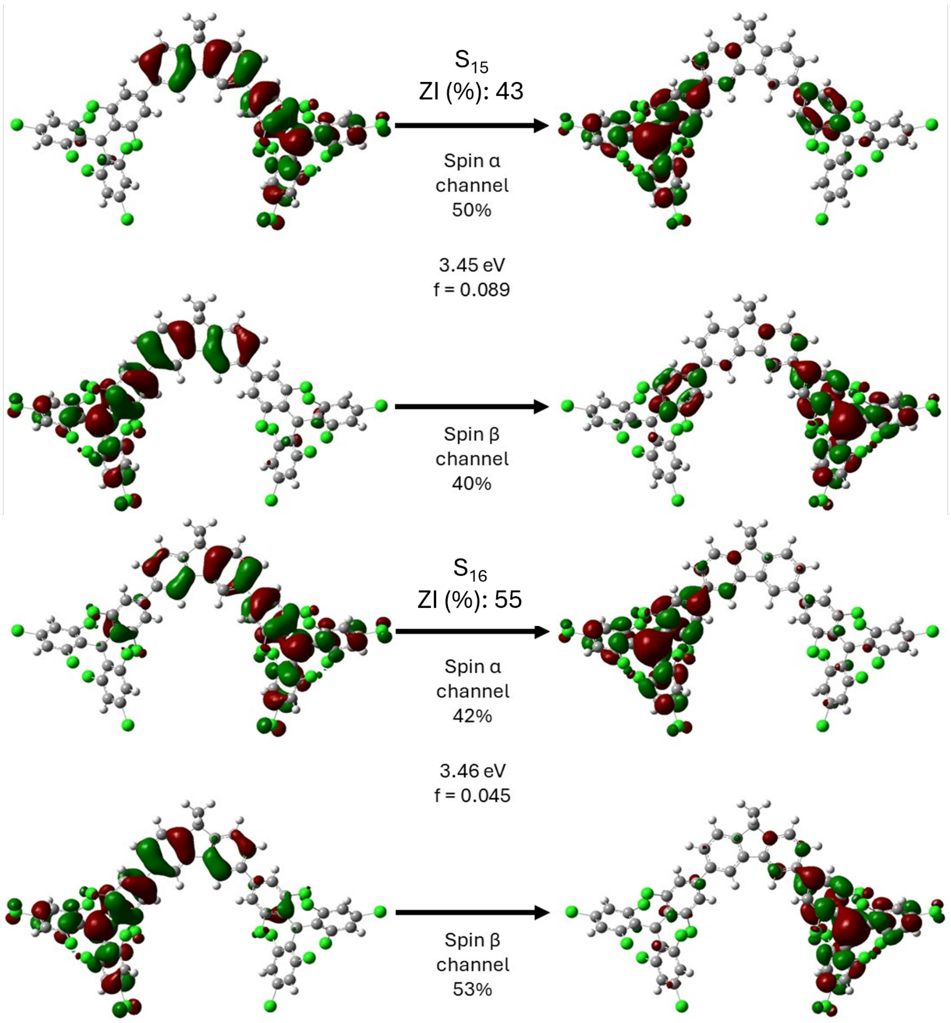

Figure 57: Hole-particle natural transition orbitals of S15 and S16 ($ZI_{1/2}$) as computed at the Frank-Condon region in gas-phase.

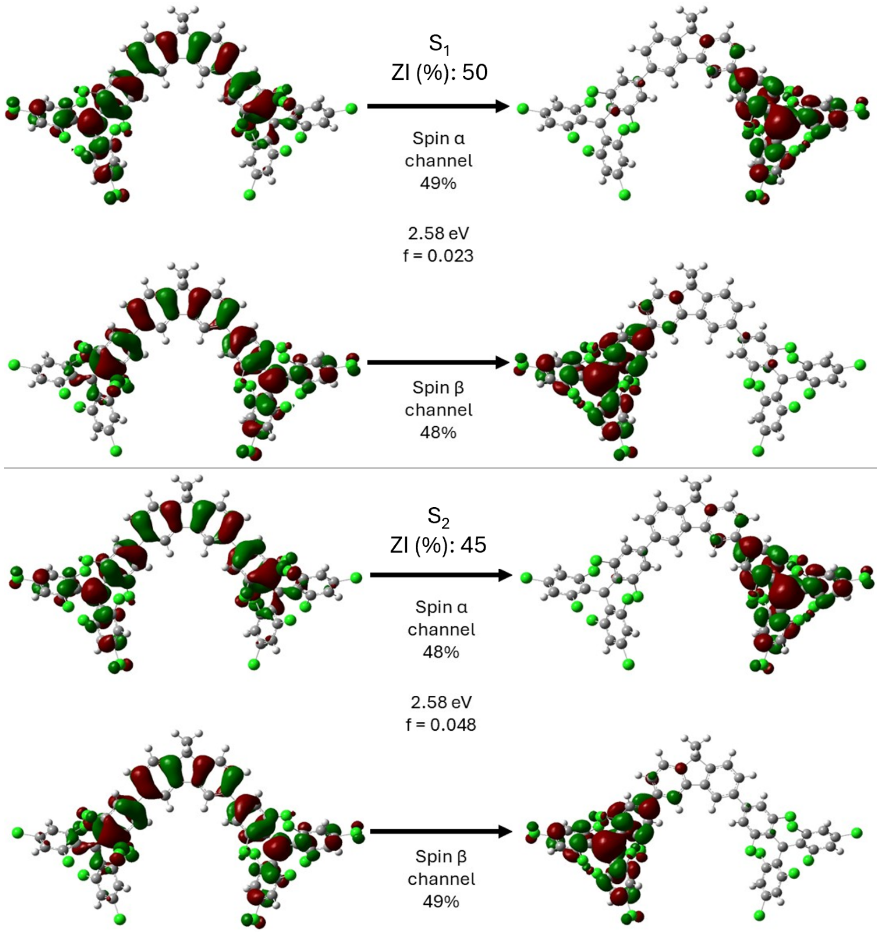

Figure 58: Hole-particle natural transition orbitals of S1 and S2 ($ZI_{1/2}$) as computed at the Frank-Condon region in ε = 2.

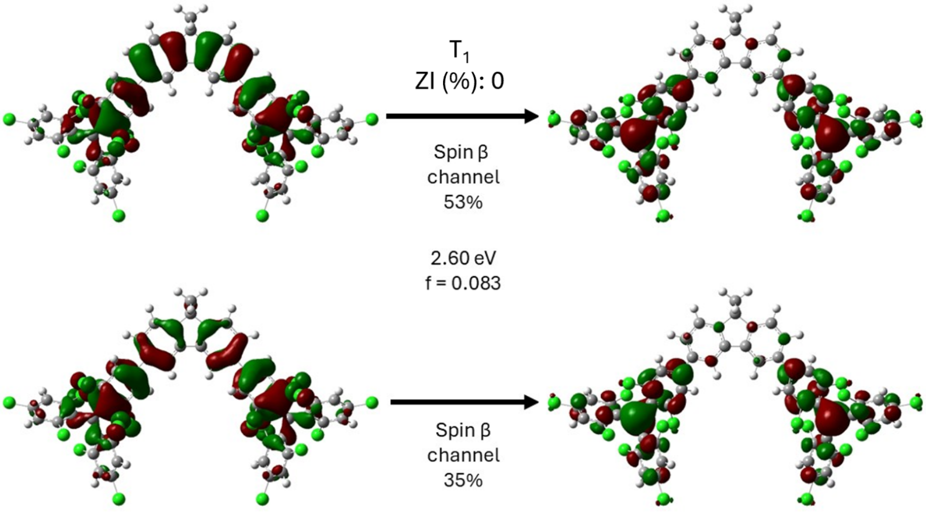

Figure 59: Hole-particle natural transition orbitals of T1 (^{3}CT) as computed at the Frank-Condon region in ε = 2.

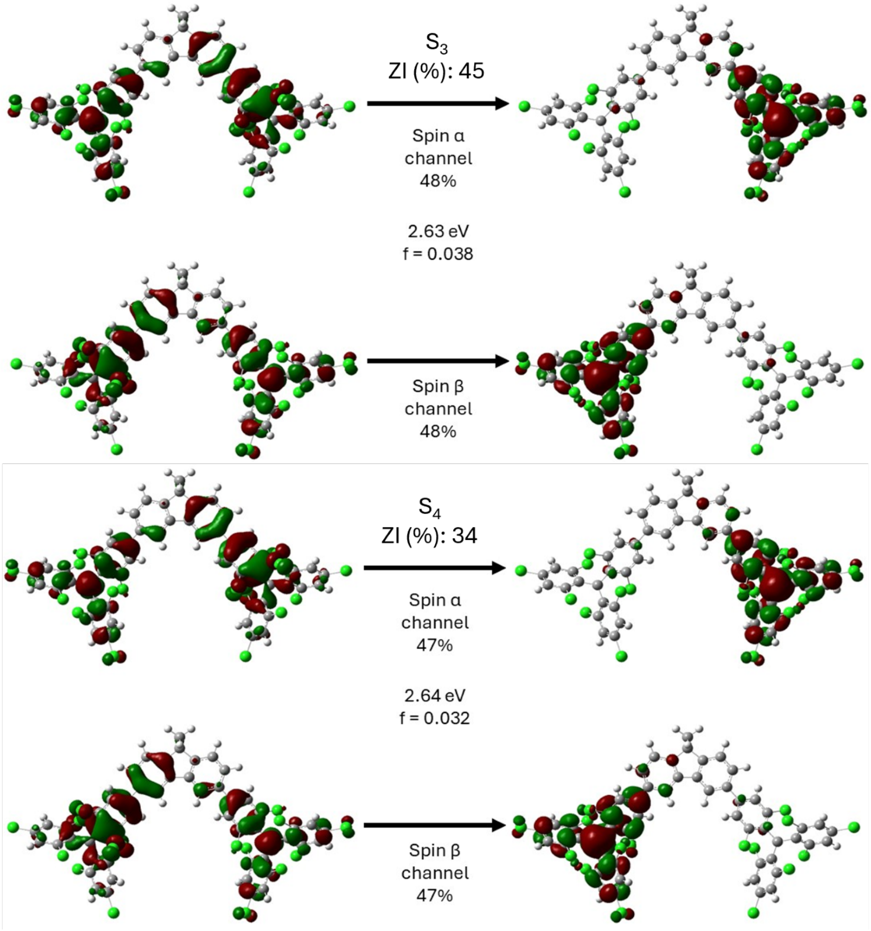

Figure 60: Hole-particle natural transition orbitals of S3 and S4 ($^{1}CT_{1/2}$) as computed at the Frank-Condon region in ε = 2.

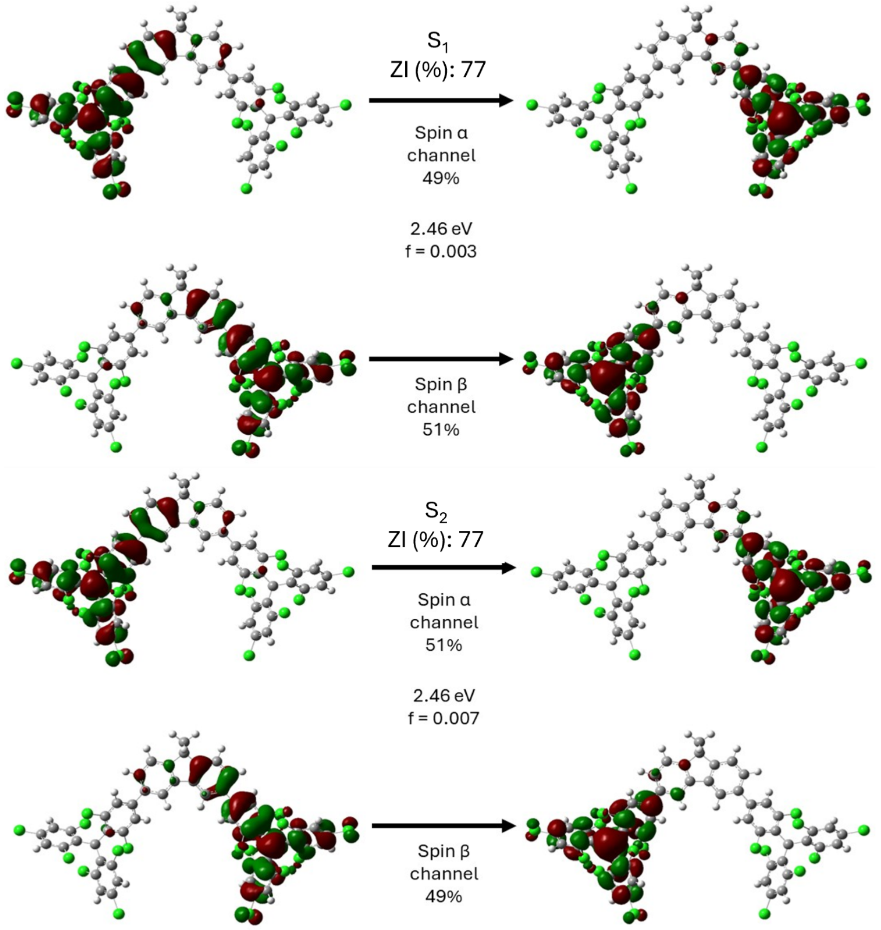

Figure 61: Hole-particle natural transition orbitals of S1 and S2 (ZI$_{1/2}$) as computed at the Frank-Condon region in ε = 2.37.

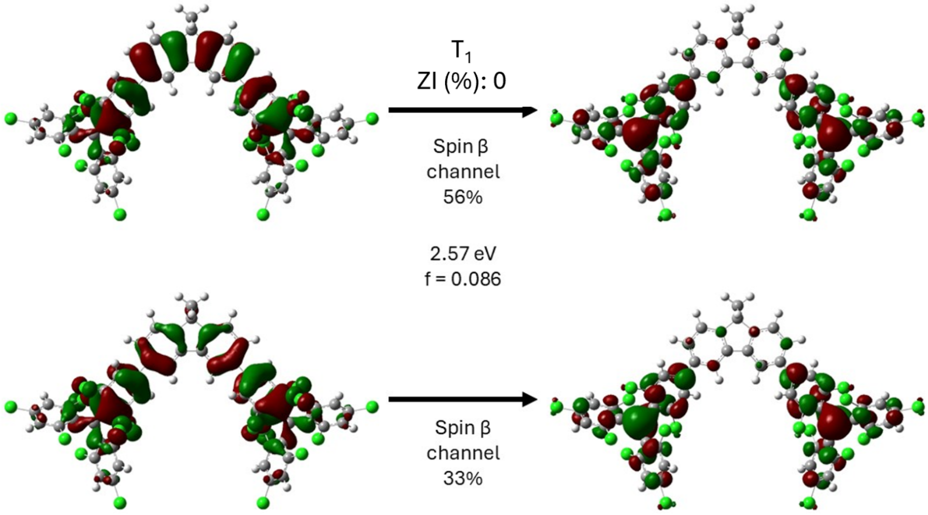

Figure 62: Hole-particle natural transition orbitals of T1 (^{3}CT) as computed at the Frank-Condon region in ε = 2.37.

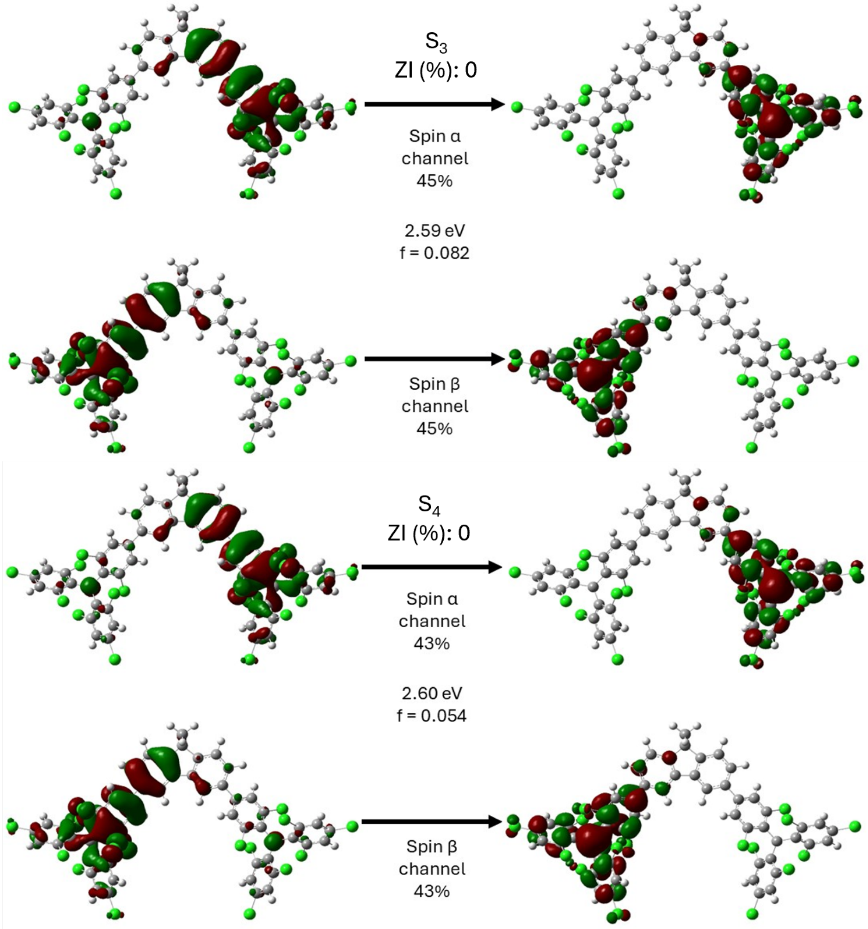

Figure 63: Hole-particle natural transition orbitals of S3 and S4 ($^1CT_{1/2}$) as computed at the Frank-Condon region in ε = 2.37.

X.3 Charge-transfer character.

A Löwdin population analysis was performed on the hole and electron densities for the singlet and triplet states of the diradical. The absolute values of the integrated hole and electron density over the two TTM units range from 0.65 to 0.80 in the singlet excited state, indicative of a rather pure ZI excitation (though we consider that the small admixture of excitations involving the fluorene central unit must contribute to the non-zero oscillator strength/radiative decay rate of the singlet). The triplet excited states have a more complex nature involving strong mixing between TTM-localized excitations and fluorene-to-TTM CT character, though the fluorene contribution is sensitive to both molecular conformation and dielectric relaxation.

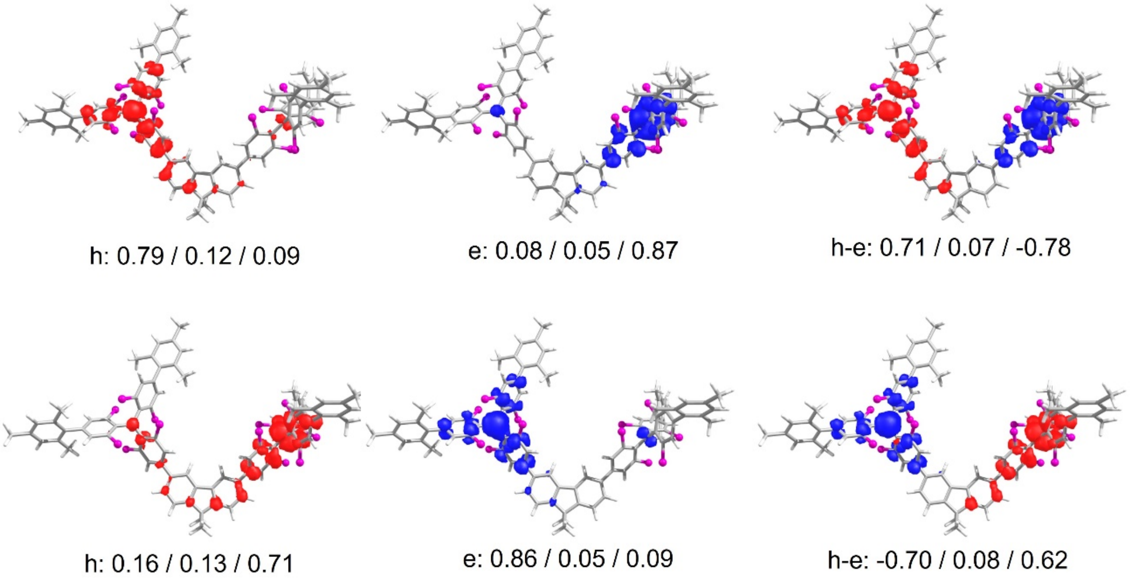

Figure 64: Hole (left), electron (middle) and charge density difference (right) for the two ZI excited states (top and bottom). *Positive charge densities are in red and negative charge densities in blue. Values of the total hole, electron and hole-electron difference are provided for TTM_1, fluorene and TTM_2 fragments.*

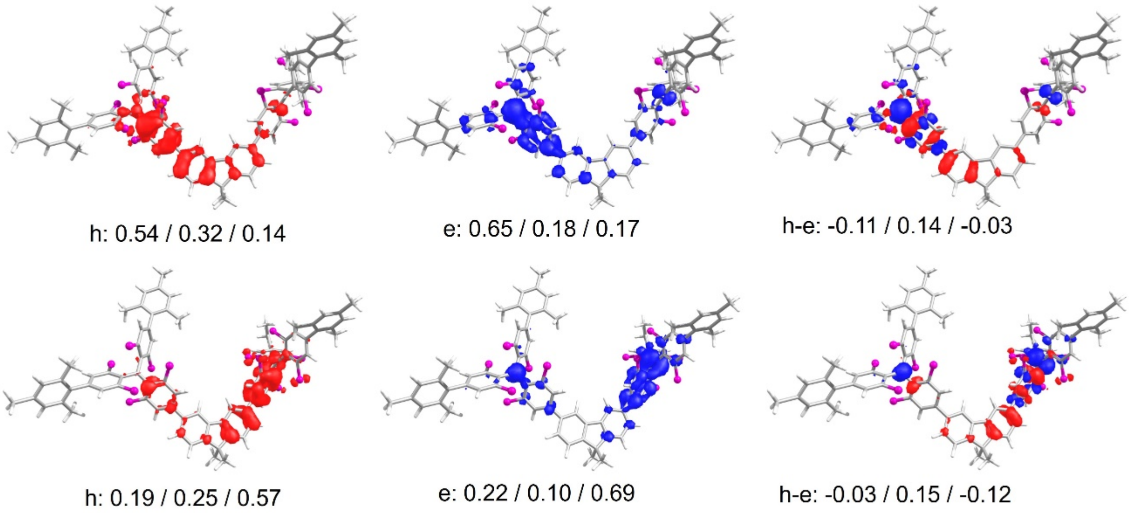

Figure 65: Hole (left), electron (middle) and charge density difference (right) for the triplet excited states (top and bottom). *Positive charge densities are in red and negative charge densities in blue. Values of the total hole, electron and hole-electron difference are provided for TTM_1, fluorene and TTM_2 fragments.*

X.4 Dielectric and conformational relaxation.

We next performed excited-state optimizations of the T1 (^{3}CT) and S1 (ZI) states to explore the conformational relaxation effects at ε = 2 and 2.37. Upon S1 (ZI) optimization, the dihedral angle connecting the fluorene to the TTM (on the right side) changes from ~37° to ~25°, along with a general shrink of C-C bonds connecting the

different molecular moieties on the right side of the diradical. On the other hand, upon T1 (^{3}CT) optimization, the dihedral angle connecting the fluorene to the TTM changes from ~37° to ~18°, again accompanied by a contraction of the C-C bonds as in the optimized S1 case. In terms of relative total energies with respect the optimized ground-state geometries, the relaxed T1 structure is higher in energy of 0.22 eV, while the relaxed S1 of 0.26 eV. In Figure 66 and Figure 70 are shown the energy diagrams for ε = 2 and 2.37, where all the values (in eV) are adiabatic, referring thus to the optimized ground-state geometries.

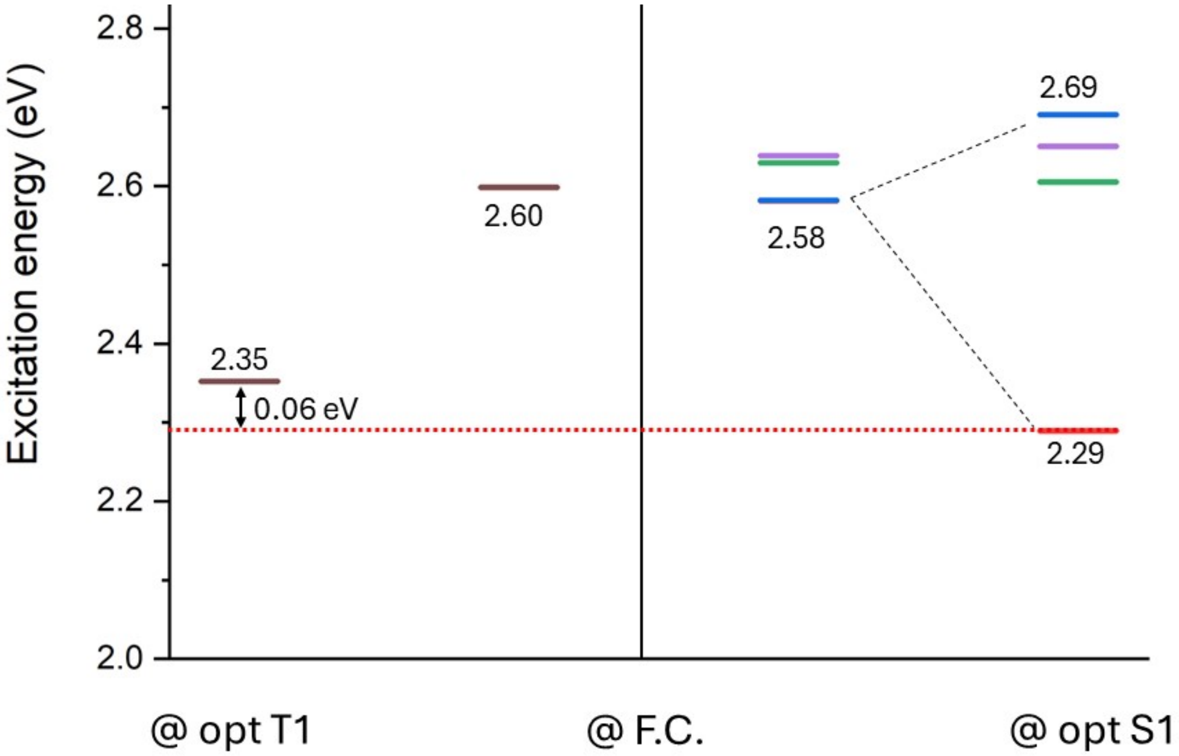

***Figure 66: Energy diagram at ε = 2.** On the left side of the vertical black line, the triplet excited-state energies computed at the Frank-Condon region and at the optimized T1, while on the right side the singlet excited-state energies computed at the Frank-Condon region and at the optimized S1. Colors: red ZI_1, blue ZI_2, dark cyan $^1CT_1$, violet $^1CT_2$, brown ^3CT.*

Figure 67-70 show the natural transition orbitals (NTOs) of the relevant transitions as computed at ε=2 for the relaxed S1 and T1 structures. Adiabatic excitation energies and the oscillator strength (f) of a given transition are reported as well.

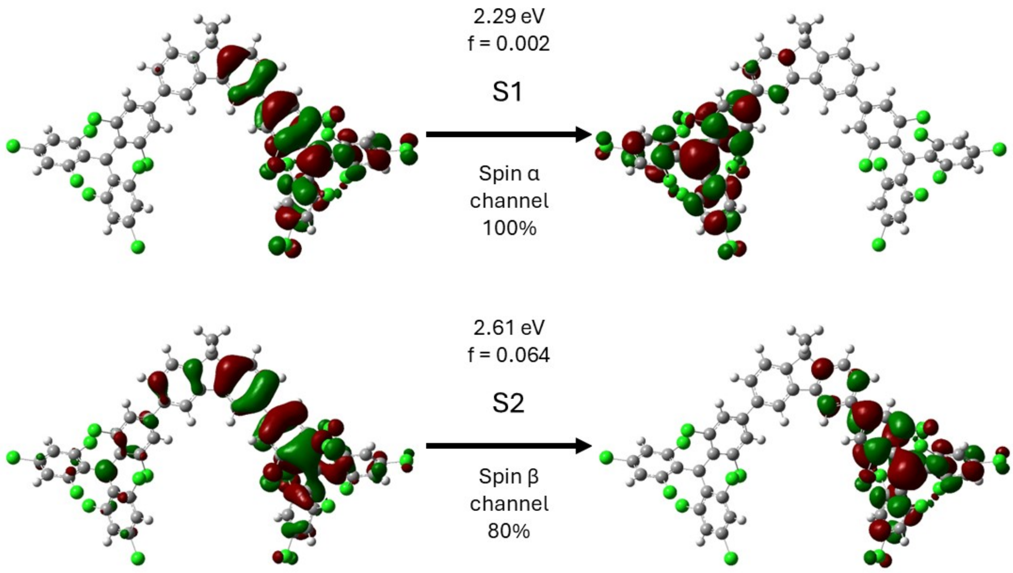

Figure 67: Hole-particle natural transition orbitals of S1 (ZI$_1$) and S2 (^{1}CT$_1$) as computed at the optimized S1 structure in ε = 2.

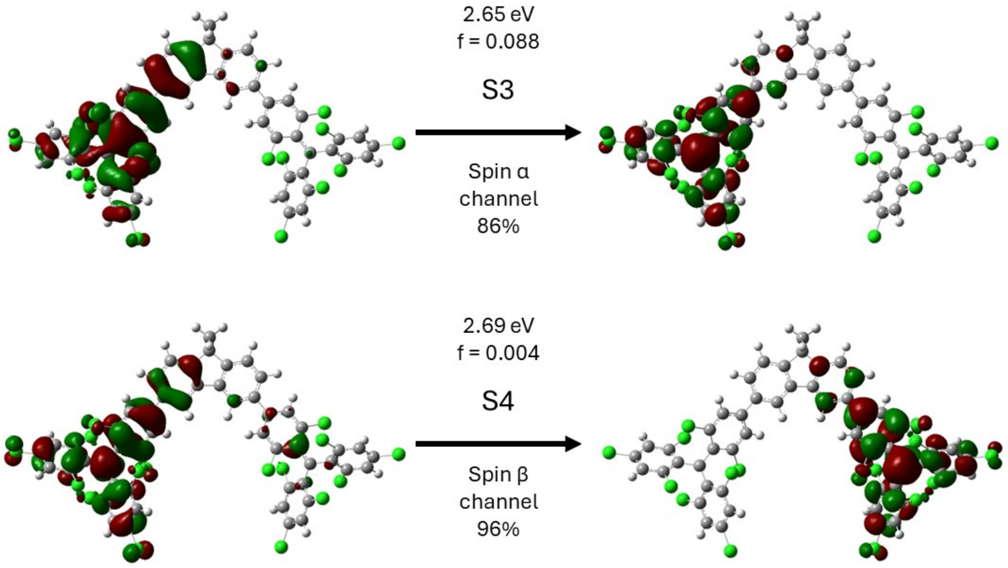

Figure 68: Hole-particle natural transition orbitals of S3 (^{1}CT$_2$) and S4 (ZI$_2$) as computed at the optimized S1 structure in ε = 2.

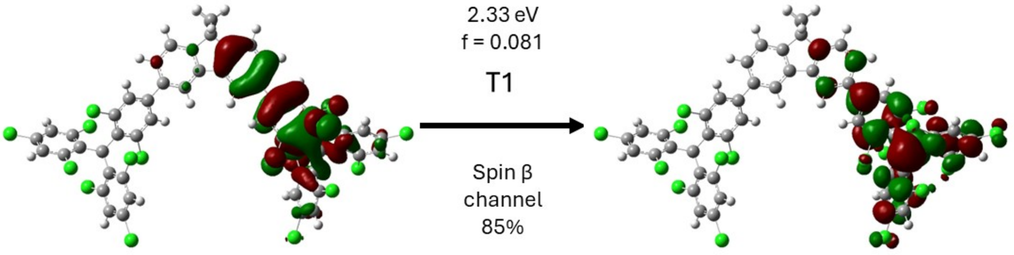

Figure 69: Hole-particle natural transition orbitals of T1 (^{3}CT) as computed at the optimized T1 structure in ε = 2.

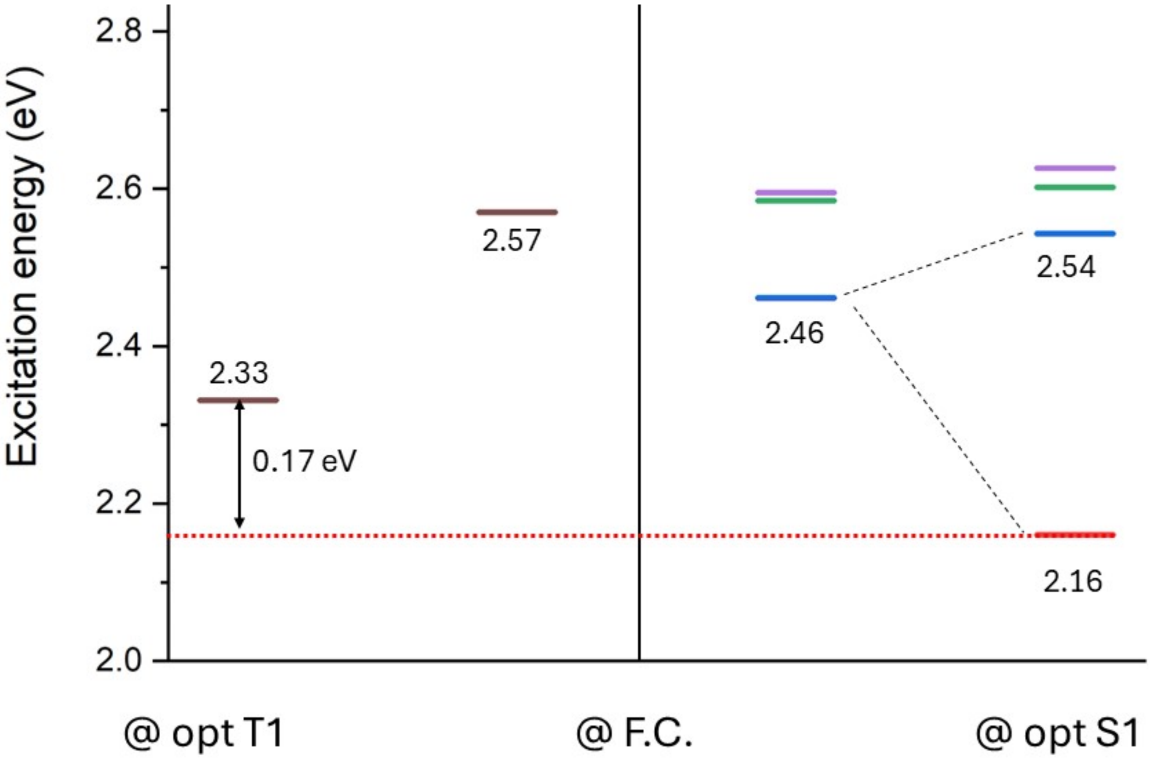

Figure 70: Energy diagram at ε = 2.37. On the left side of the vertical black line, the triplet excited-state energies computed at the Frank-Condon region and at the optimized T1, while on the right side the singlet excited-state energies computed at the Frank-Condon region and at the optimized S1. Colors: red ZI_1, blue ZI_2, dark cyan $^1CT_1$, violet $^1CT_2$, brown ^3CT.

Figure 71-73 show the natural transition orbitals (NTOs) of the relevant transitions as computed at ε=2.37 for the relaxed S1 and T1 structures. Adiabatic excitation energies and the oscillator strength (f) of a given transition are reported as well.

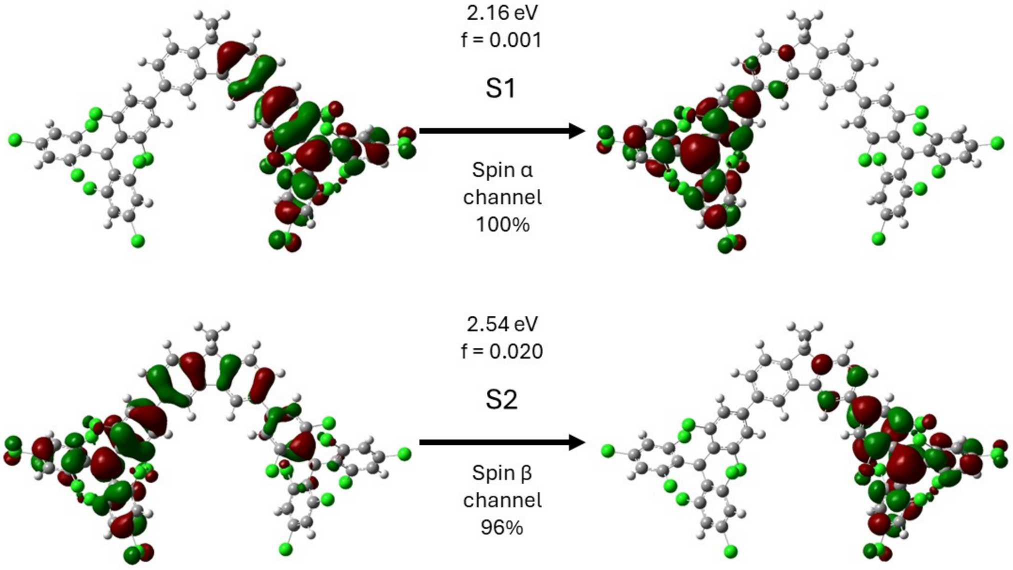

Figure 71: Hole-particle natural transition orbitals of S1 (ZI_1) and S2 (ZI_2) as computed at the optimized S1 structure in ε = 2.37.

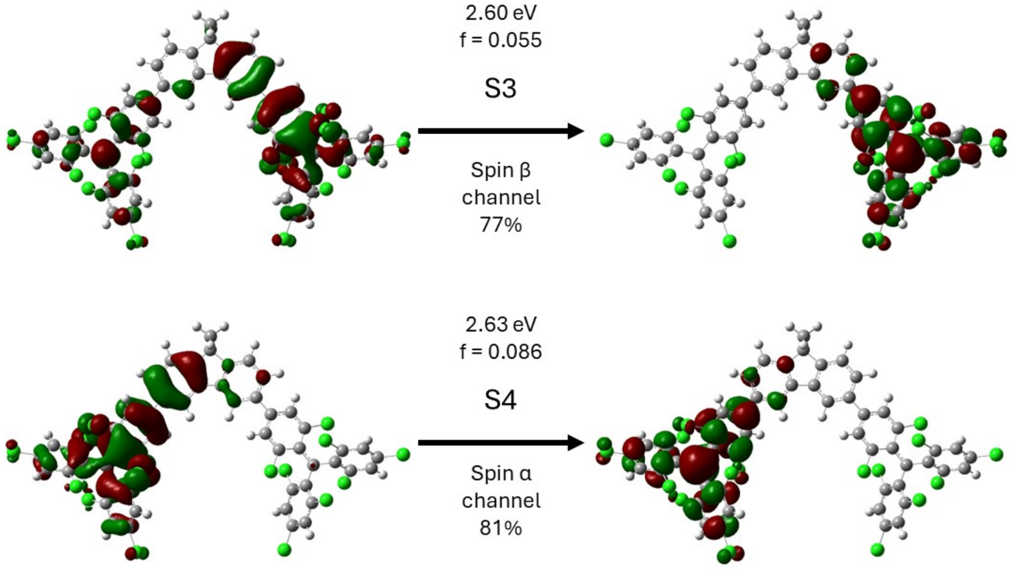

Figure 72: Hole-particle natural transition orbitals of S3 ($^1CT_1$) and S4 ($^1CT_2$) as computed at the optimized S1 structure in ε = 2.37.

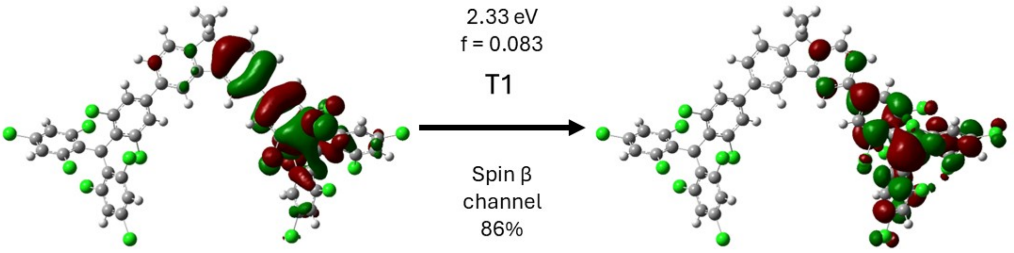

Figure 73: Hole-particle natural transition orbitals of T1 (^{3}CT) as computed at the optimized T1 structure in ε = 2.37.

X.5 Radiative lifetimes.

Radiative lifetimes τ were calculated following to the equation:

$$\frac{1}{\tau} = k_{rad} = \frac{E_\alpha^3 \left|\mu_{wa,k=0}\right|^2}{3\varepsilon_0 \pi \hbar^4 c^3} \cdot \frac{n\left(n^2+2\right)^2}{9}$$

where the vertical excitation energies E_α and transition dipole moments $\mu_{wa,k=0}$ were obtained both at the Franck-Condon region and at the relaxed T1 and S1 excited states as computed for ε = 2 and 2.37, and n is the refractive index of toluene (*n* = 1.497).

	E_α (eV)	$\mu_{wa,k=0}$ (Debye)	τ (ns)
S1 @ F.C.	2.58	1.54	50
S2 @ F.C.	2.58	2.22	24
S1 @ opt S1	2.03	0.44	1246
S2 @ opt S1	2.34	2.68	22
T1 @ F.C.	2.60	2.91	14
T1 @ opt T1	2.13	3.16	21

Table 4: Vertical excitation energies, transition dipole moments, and calculated radiative lifetimes for T1 and S1 at different optimized geometries at ε = 2.

	E_α (eV)	$\mu_{wa,k=0}$ (Debye)	τ (ns)
S1 @ F.C.	2.46	0.56	442
S2 @ F.C.	2.46	0.85	188
S1 @ opt S1	1.90	0.39	1950
S2 @ opt S1	2.29	1.53	72
T1 @ F.C.	2.57	2.97	14
T1 @ opt T1	2.12	3.22	21

Table 5: Vertical excitation energies, transition dipole moments, and calculated radiative lifetimes for T1 and S1 at different optimized geometries at ε = 2.37.

At the Frank-Condon region, the emissive radiative lifetimes of T1 are computed to be 14 ns for both values of the dielectric constant, while those of S1 are 50 ns and 442 ns, at ε = 2 and 2.37 respectively. At ε = 2 the S1 and S2 state show a mixed ZI-CT character and have both a larger transition dipole moment and a finite oscillator strength (f = 0.023 and 0.048, see Figure 58). On the other hand, at ε = 2.37 the S1 and S2 state are already a quasi-pure ZI states, showing a smaller transition dipole moment and a negligible oscillator strength (f = 0.003 and 0.007, see Figure 57). Upon S1 optimization, the relaxed ZI state becomes pure for both values of the dielectric constant, thus further reducing its transition dipole moment and oscillator strength (see Figure 67 and S.71). Consequently, the radiative lifetimes exceed 1 μs, a timescale too long if compared to the experimental value of ~100 ns.

At this point, we reasoned that the full conformational relaxation at the excited-state level might be hindered at the solid-state, and we explored the case where the low-frequency molecular degrees of freedom (i.e., torsion angles) were kept frozen at their ground-state values, while bond lengths and angles were allowed to relax at the excited state. For such partially relaxed T1 and S1 geometries:

	E_α (eV)	$\mu_{wa,k=0}$ (Debye)	τ (ns)
S1 @ frozen F.C.	2.46	0.82	202
S2 @ frozen F.C.	2.52	2.37	23
T1 @ frozen F.C.	2.53	1.34	14

Table 6: Vertical excitation energies, transition dipole moments, and calculated radiative lifetimes for T1 and S1 at partially relaxed geometries at ε = 2.

	E_α (eV)	$\mu_{wa,k=0}$ (Debye)	τ (ns)
S1 @ frozen F.C.	2.32	0.47	734
S2 @ frozen F.C.	2.42	0.89	182
T1 @ frozen F.C.	2.45	1.20	18

Table 7: Vertical excitation energies, transition dipole moments, and calculated radiative lifetimes for T1 and S1 at partially relaxed geometries at ε = 2.37.

X.6 Excited-state absorption spectra.

TDA TDDFT calculations on the fully optimized S1 and T1 structures were performed at the OT-SRSH LC-ωhPBE/cc-pVDZ level of theory, by setting ε = 2.37. Excitation energies and oscillator strengths between excited states were obtained with the Multiwfn tool(*13*).

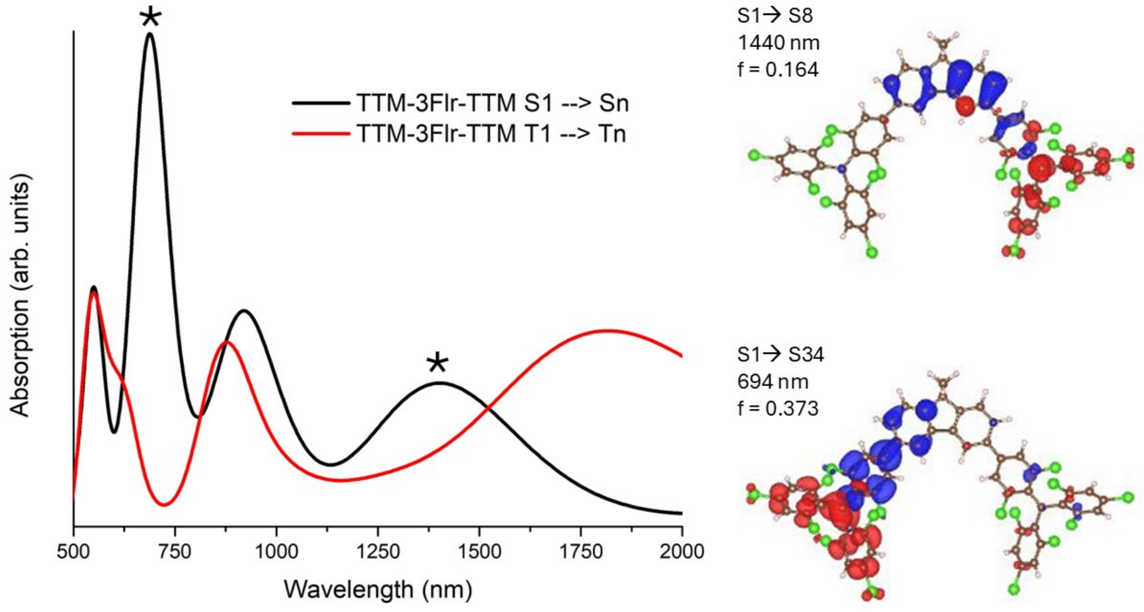

Figure 74: Excited-state absorption spectra (S1 → Sn and T1 → Tn) of TTM-3FIr-TTM diradical, along with transition density maps at 1440 nm and 694 nm.

XI. Analytical treatment of the diradical

We perform an analysis of transition dipole moments based on the treatment of doublet emitters by Abdurahman and Hele *et al* (*14*) which allows us to extend this theoretical framework set out for prior doublet emitters for diradical emitters.

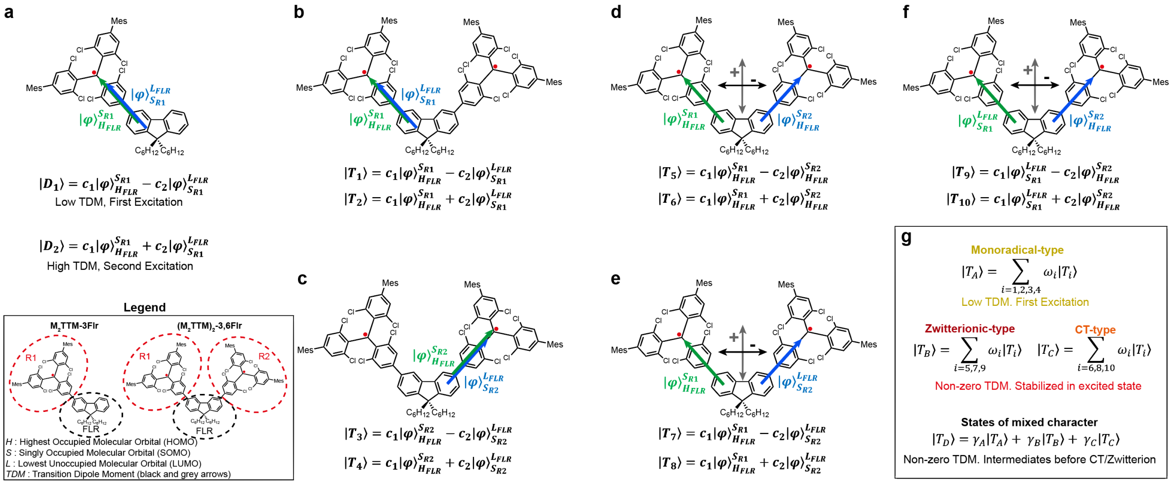

Figure 75: Analysing transitions using excitation dipoles in the alternant Fluorene bridged monoradical and diradical. (a) *Excited state wavefunctions D_1 and D_2 can be constructed by the in-phase and anti-phase combinations of the HOMO→SOMO from fluorene to radical (green arrow) and the SOMO→LUMO from radical to fluorene (blue arrow).* ***(b-c)*** *Triplet excited states constructed from in phase and anti-phase combinations of the local monoradical-like excitations centered on the left-hand radical (R1) or on the right-hand radical (R2). Here the other radical remains un-excited but its spin counted in the net spin of the excited state.* ***(d-f)*** *In phase and anti-phase combinations of non-local and symmetry broken excitations. Excitations are on both radical centres. In (d-f) the antisymmetric combination (black double-sided arrow) and the symmetric combination (grey double-sided arrow) are orthogonal.* ***(g)*** *These triplet wavefunctions we described in (b-f) can be used to construct more realistic electronic excited states. Some special cases are the monoradical type which have low oscillator strength to the ground state and is likely to be the first excited state, whose excitation dipole is localised on the individual M_2TTM-3Flr units and is an equal linear combination of both the fragments. The other special cases are from the combinations of the non-local excitation dipoles that can lead to the CT-type (grey arrows in d-f) or the charge-separated zwitterionic (black arrows in d-f) states. Both of these states have broken electronic symmetry and are lower in energy than the initial monoradical type excitation (derived from simple summation of energies maintaining the sign of the combination) and can be transiently populated, these states have higher transition dipole moment, when compared to the monoradical excitation, back to the ground state and can be bright. The final set of states are arbitrary non-zero combinations of all states which are complex and are probably transition intermediates between the extreme cases described before. Thus the diradical is brighter due to the larger number of combinations with a non-vanishing excitation dipole that adds oscillator strength to CT-type and zwitterionic states. The role of alternant symmetry on simple monoradical type doublet excitons has been explored before Abdurahman and Hele et al* (*14*) *and is the basis for this analysis.*

XII. Effect of concentration

We find that upon increasing the concentration to 1wt% of diradical in polystyrene the PLQE drops sharply. In the 1wt% films the PL spectrum red-shifts to a maxima of 700nm and becomes completely unstructured with no obvious temperature dependence in any wavelength associated with the triplet or singlet of the system.

Concentration	PLQE(%)
**1.0**	23
**0.75**	31
**0.5**	30
**0.25**	72
**0.125**	89
**<0.1**	92

Table 8: Dependence of PLQE on the concentration of the M2TTM-3FIr-M2TTM diradical in polystyrene.

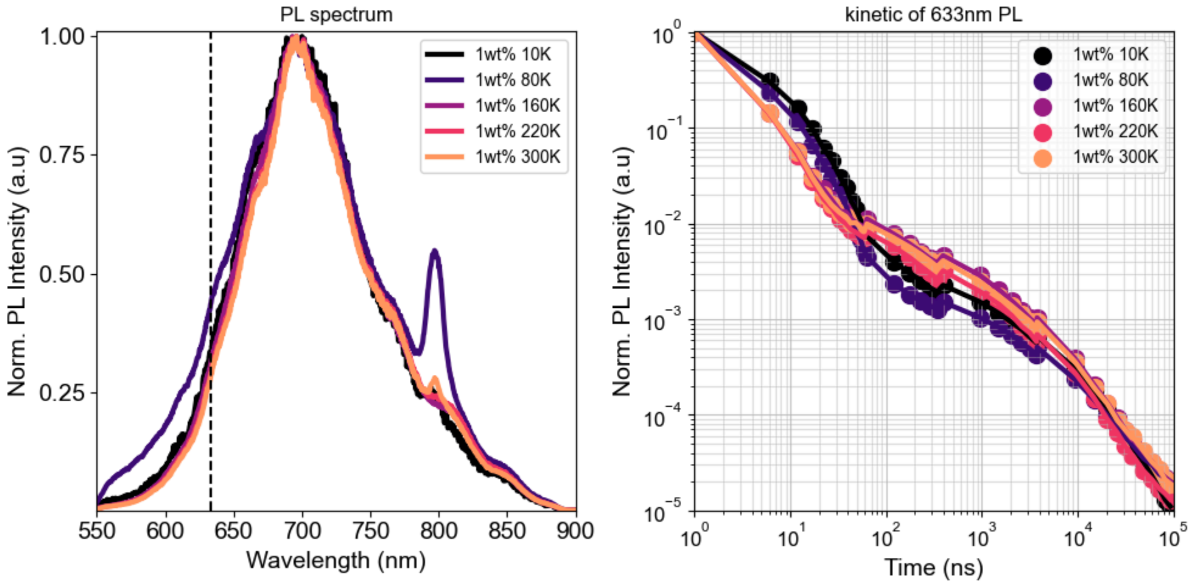

Figure 76: PL spectra at varying temperatures for a 1.0wt% M2TTM-3FIr-M2TTM doped polystyrene film. *The kinetics of the 640nm region has been shown in the right-hand panel with no obvious temperature dependence. The 800 nm sharp feature is due to residual fundamental scatter from the source laser excitation into the NOPA.*

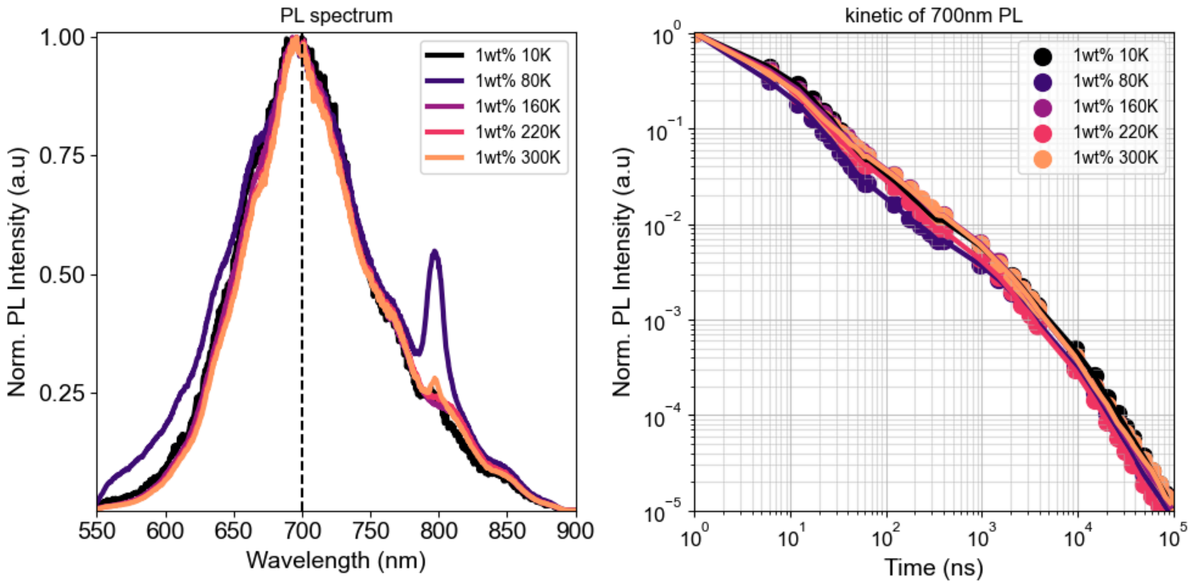

Figure 77: PL spectra at varying temperatures for a 1.0wt% M2TTM-3FIr-M2TTM doped polystyrene film. *The kinetics of the 700nm region has been shown in the right-hand panel with no obvious temperature dependence.*

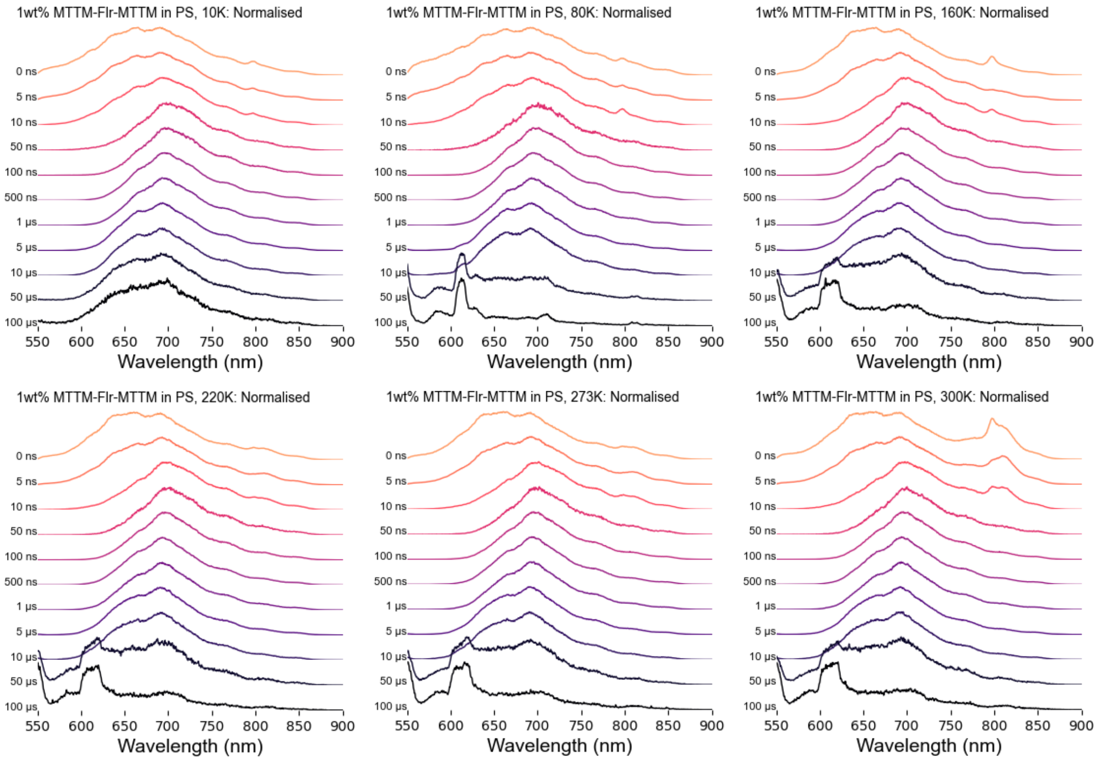

Figure 78: Temperature dependent time resolved PL spectra of 1wt% M2TTM-3FIr-M2TTM down to 10K shows that the spectral dynamics is unchanged at nearly every temperature. *Some delayed blue PL is observed but there is no thermal activation for this. A reasonable explanation is the formation of aggregated closed shell species that results in this unstructured, low yield emission. This aggregate, and its closed shell character is evidenced by the lack of an ESR signal at this concentration.*

XIII. 10nM diradical doped crystals

1,3,5-Trichlorobenzene ($PhCl_3$) was purchased from Sigma Aldrich, Product catalog number T54607-100G. 30g of $PhCl_3$ was dissolved in 25 mL of Toluene in a 100 mL conical flask by continuously stirring the solution at 50°C. To maintain 10^{-8} M concentration of diradical with respect to the $PhCl_3$ lattice we require 2.905 μg of M_2TTM-3FIr-M_2TTM. To achieve this we prepare a 10^{-5} M toluene solution of the diradical, from which we draw 165 μL and add it to the previously prepared solution of $PhCl_3$ and the solvent was allowed to slowly evaporate producing long needle-like single crystals that contained 10nM of the diradical. In order to further clean the surface we polish the crystals by washing with hexane (dissolves the diradical more preferentially than the $PhCl_3$ host) 5 times. Shown in Figure 79 below are the crystals.

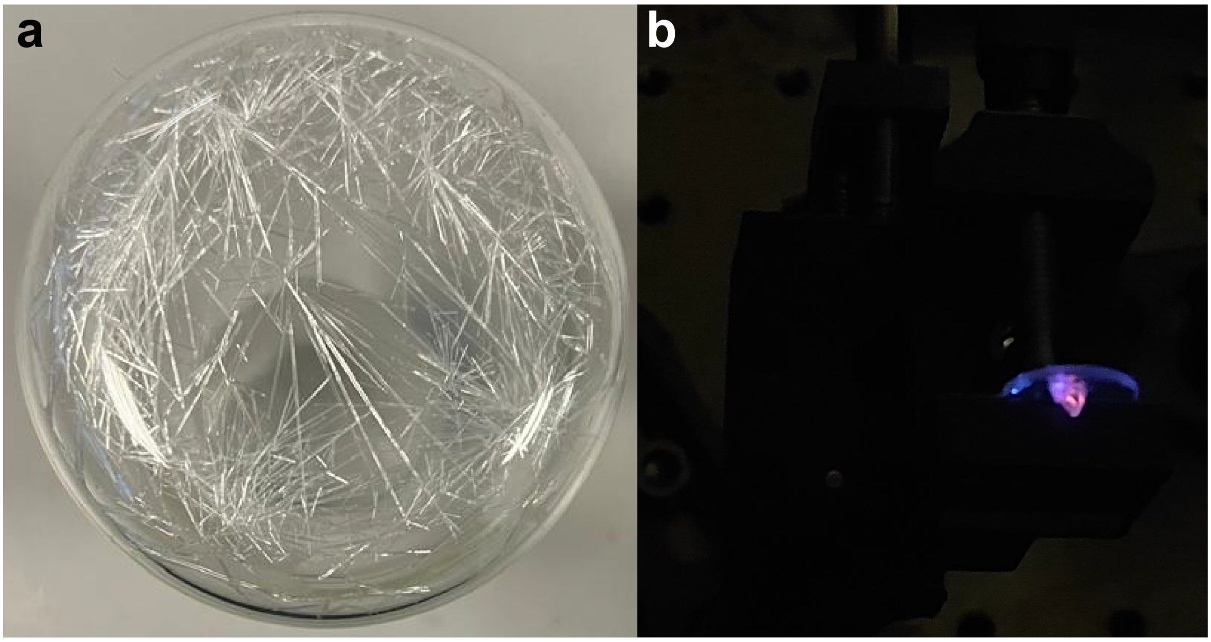

Figure 79: 10nM M2TTM-3FIr-M2TTM doped PhCl3 crystals. ***(a)*** *Picture of the needle-shaped long crystals grown via solvent evaporation.* ***(b)*** *Emission from multiple crystals bunched and mounted on a quartz substrate.*

We confirmed the optical properties of the diradical even under such dilutions in a lattice using transient photoluminescence studies as shown in Figure 80 below.

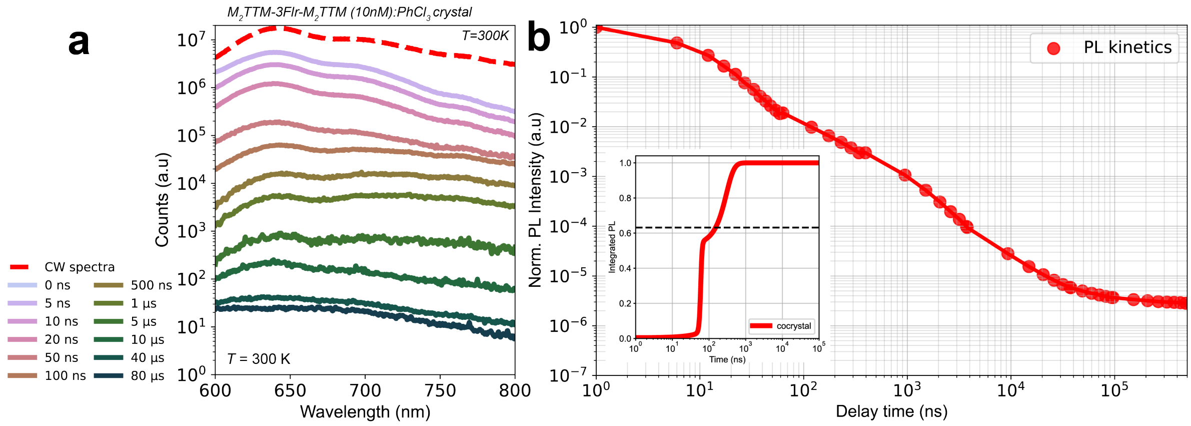

Figure 80: Transient Photoluminescence in crystals: ***(a)*** *Spectral slice of the TRPL at 300 K.* ***(b)*** *Kinetics of the full PL.*

References

1. P. Murto, R. Chowdhury, S. Gorgon, E. Guo, W. Zeng, B. Li, Y. Sun, H. Francis, R. H. Friend, H. Bronstein, Mesitylated trityl radicals, a platform for doublet emission: symmetry breaking, charge-transfer states and conjugated polymers. *Nat Commun* 14, 4147 (2023).

2. P. ’Ghosh, A. ’Alvertis, A. ’Gillett, S. ’Dong, A. ’Sneyd, H.-H. ’Cho, E. ’Evans, B. ’Monserrat, F. ’Li, C. ’Schnedermann, R. ’Friend, A. ’Rao, “Suppressing non-radiative losses in organic semiconductors caused by high-frequency molecular vibrations”

(Cambridge, 2023); https://assets.researchsquare.com/files/rs-2084680/v1/b4b24ec2-92a0-4722-b121-ea587bdb33f8.pdf?c=1686074345.

3. K. Brunner, A. van Dijken, H. Börner, J. J. A. M. Bastiaansen, N. M. M. Kiggen, B. M. W. Langeveld, Carbazole Compounds as Host Materials for Triplet Emitters in Organic Light-Emitting Diodes: Tuning the HOMO Level without Influencing the Triplet Energy in Small Molecules. *J Am Chem Soc* 126, 6035–6042 (2004).

4. Y. Song, W. Xu, D. Zhu, Synthesis and properties of cyclic ethylene-bridged 3,6-fluorene dimer and its linear analogues. *Tetrahedron Lett* 51, 4894–4897 (2010).

5. D. Reger, P. Haines, K. Y. Amsharov, J. A. Schmidt, T. Ullrich, S. Bönisch, F. Hampel, A. Görling, J. Nelson, K. E. Jelfs, D. M. Guldi, N. Jux, A Family of Superhelicenes: Easily Tunable, Chiral Nanographenes by Merging Helicity with Planar π Systems. *Angewandte Chemie International Edition* 60, 18073–18081 (2021).

6. C.-H. Lee, Y.-Y. Lai, S.-W. Cheng, Y.-J. Cheng, Synthesis and Supramolecular Assembly of Pentacyclic Dithienofluorene and Diselenophenofluorene Derivatives. *Org Lett* 16, 936–939 (2014).

7. C. Wamser, J. Otvos, M. Calvin, “Magnetic-field effects on photosensitized electron-transfer reactions” (Berkeley, CA (United States), 1981); https://doi.org/10.2172/6620086.

8. P. K. Poddutoori, Y. E. Kandrashkin, C. O. Obondi, F. D’Souza, A. van der Est, Triplet electron transfer and spin polarization in a palladium porphyrin–fullerene conjugate. *Physical Chemistry Chemical Physics* 20, 28223–28231 (2018).

9. A. Mizuno, R. Matsuoka, S. Kimura, K. Ochiai, T. Kusamoto, Spin-Correlated Luminescence of a Carbazole-Containing Diradical Emitter: Single-Molecule Magnetoluminescence and Thermally Activated Emission. *J Am Chem Soc* 146, 18470–18483 (2024).

10. A. Rybalko, S. Rubets, E. Rudavskii, V. Tikhiy, S. Tarapov, R. Golovashchenko, V. Derkach, Resonance absorption of microwaves in <math display="inline"> <mrow> <mi>He</mi> <mspace width="0.3em"/> <mi>II</mi> </mrow> </math> : Evidence for roton emission. *Phys Rev B* 76, 140503 (2007).

11. M. J. Frisch, G. W. Trucks, H. B. Schlegel, G. E. Scuseria, M. A. Robb, J. R. Cheeseman, G. Scalmani, V. Barone, G. A. Petersson, H. Nakatsuji, X. Li, M. Caricato, A. V Marenich, J. Bloino, B. G. Janesko, R. Gomperts, B. Mennucci, H. P. Hratchian, J. V Ortiz, A. F. Izmaylov, J. L. Sonnenberg, D. Williams-Young, F. Ding, F. Lipparini, F. Egidi, J. Goings, B. Peng, A. Petrone, T. Henderson, D. Ranasinghe, V. G. Zakrzewski, J. Gao, N. Rega, G. Zheng, W. Liang, M. Hada, M. Ehara, K. Toyota, R. Fukuda, J. Hasegawa, M. Ishida, T. Nakajima, Y. Honda, O. Kitao, H. Nakai, T. Vreven, K. Throssell, J. A. Montgomery Jr., J. E. Peralta, F. Ogliaro, M. J. Bearpark, J. J. Heyd, E. N. Brothers, K. N. Kudin, V. N. Staroverov, T. A. Keith, R. Kobayashi, J. Normand, K. Raghavachari, A. P. Rendell, J. C. Burant, S. S. Iyengar, J. Tomasi, M. Cossi, J. M. Millam, M. Klene, C. Adamo, R. Cammi, J. W. Ochterski, R. L. Martin, K. Morokuma, O. Farkas, J. B. Foresman, D. J. Fox, Gaussian˜16 Revision C.01. [Preprint] (2016).

12. N. Gonzalez-Pato, D. Blasi, D. M. Nikolaidou, F. Bertocchi, J. Cerdá, F. Terenziani, N. Ventosa, J. Aragó, A. Lapini, J. Veciana, I. Ratera, Nanothermometer Based on Polychlorinated Trityl Radicals Showing Two-Photon Excitation and Emission in the

Biological Transparency Window: Temperature Monitoring of Biological Tissues. *Small Methods* 8 (2024).

13. T. Lu, F. Chen, Multiwfn: A multifunctional wavefunction analyzer. *J Comput Chem* 33, 580–592 (2012).

14. A. Abdurahman, T. J. H. Hele, Q. Gu, J. Zhang, Q. Peng, M. Zhang, R. H. Friend, F. Li, E. W. Evans, Understanding the luminescent nature of organic radicals for efficient doublet emitters and pure-red light-emitting diodes. *Nat Mater* 19, 1224–1229 (2020).
